# Targeted panel sequencing of pharmacogenes and oncodrivers in colorectal cancer patients reveals genes with prognostic significance

**DOI:** 10.1186/s40246-024-00644-2

**Published:** 2024-07-19

**Authors:** Lucie Heczko, Václav Liška, Ondřej Vyčítal, Ondřej Fiala, Simona Šůsová, Viktor Hlaváč, Pavel Souček

**Affiliations:** 1 Biomedical Center, Faculty of Medicine in Pilsen, Charles University, alej Svobody 1655/76, Pilsen, 323 00 Czech Republic; 2 Toxicogenomics Unit, National Institute of Public Health, Prague, Czech Republic; 3 Department of Surgery, Faculty of Medicine and University Hospital in Pilsen, Charles University, Pilsen, Czech Republic; 4 Department of Oncology and Radiotherapeutics, Faculty of Medicine and University Hospital in Pilsen, Charles University, Pilsen, Czech Republic

**Keywords:** Colorectal, Carcinoma, Pharmacogene, Oncodriver, Sequencing, Prognosis, Drug, Resistance

## Abstract

**Background:**

Colorectal cancer is still the second leading cause of cancer-related deaths and thus biomarkers allowing prediction of the resistance of patients to therapy and estimating their prognosis are needed. We designed a panel of 558 genes with pharmacogenomics records related to 5-fluorouracil resistance, genes important for sensitivity to other frequently used drugs, major oncodrivers, and actionable genes. We performed a target enrichment sequencing of DNA from tumors and matched blood samples of patients, and compared the results with patient prognosis stratified by systemic adjuvant chemotherapy.

**Results:**

The median number of detected variants per tumor sample was 18.5 with 4 classified as having a high predicted functional effect and 14.5 moderate effect. *APC, TP53*, and *KRAS* were the most frequent mutated genes (64%, 59%, and 42% of mutated samples, respectively) followed by *FAT4* (23%), *FBXW7*, and *PIK3CA* (16% for both). Patients with advanced stage III had more frequently *APC*, *TP53*, or *KRAS* mutations than those in stages I or II. *KRAS* mutation counts followed an increasing trend with grade (G1 < G2 < G3). The response to adjuvant therapy was worse in carriers of frameshift mutations in *APC* or 12D variant in *KRAS*, but none of these oncodrivers had prognostic value. Carriage of somatic mutations in any of the genes *ABCA13, ANK2, COL7A1, NAV3*, or* UNC80* had prognostic relevance for worse overall survival (OS) of all patients. In contrast, mutations in *FLG, GLI3*, or *UNC80* were prognostic in the same direction for patients untreated, and mutations in *COL6A3, LRP1B, NAV3, RYR1, RYR3, TCHH*, or *TENM4* for patients treated with adjuvant therapy. The first association was externally validated. From all germline variants with high or moderate predicted functional effects (median 326 per patient), > 5% frequency and positive Manhattan plot based on 3-year RFS, rs72753407 in *NFACS*, rs34621071 in *ERBB4*, and rs2444274 in *RIF1* were significantly associated with RFS, OS or both.

**Conclusions:**

The present study identified several putative somatic and germline genetic events with prognostic potential for colorectal cancer that should undergo functional characterization.

**Supplementary Information:**

The online version contains supplementary material available at 10.1186/s40246-024-00644-2.

## Background

Colorectal cancer (CRC) is the third most common cancer worldwide and the second leading cause of oncology-related deaths with an estimated 1.9 million newly diagnosed patients and 935 thousand deaths per year [1]. The all-stages 5-year survival for both sexes is approximately 65% [2]. There is, therefore, an urgent need for improving preventive measures of all kinds, including reliable biomarkers that would accurately predict the resistance of patients to therapy, potentially extending the patients’ survival. Sporadic CRC, a disease without apparent family history or inherited mutations increasing CRC risk, occurs in about 65% of all cases [3]. On the opposite, hereditary CRC like Lynch syndrome or familial polyposis coli are caused by rare inherited variants in high-penetrance susceptibility genes like *MLH1* or *APC*. Part of the tumors bear also genetic alterations that are either common genetic polymorphisms with low penetrance or their combinations, eventually inherited changes that have not been discovered yet [3]. Therefore, tumors often develop in genetically susceptible individuals by co-inheritance of multiple low-risk variants. CRC is thus a highly heterogeneous malignancy with enormous genetic differences between individuals making the treatment of patients a challenge for current medicine.

CRC treatment comprises surgical tumor removal and eventually systemic adjuvant chemotherapy, which depends on tumor staging and risk factors. Patients with stage I disease do not require adjuvant chemotherapy, while stage II patients usually receive chemotherapy only if they are considered high-risk, mostly based on number of evaluated lymph nodes, grade, tumor size, lymphovascular or perineural propagation, mismatch repair status, oncomarkers, ileus, etc. Patients of stage III receive systemic adjuvant and stage IV palliative chemotherapy. Most of the adjuvant regimens constitute chemotherapy based on 5-fluorouracil (5-FU) (de Gramonte regimen or capecitabine) for stage II, or combination regimens with oxaliplatin (FOLFOX or CAPOX) [4]. 5-FU is an anti-cancer drug widely used since 1957 [5] in the treatment of various gastrointestinal cancers. It is an analog of uracil with a structure similar to pyrimidine molecules of DNA and RNA with a fluorine atom at the C-5 position. Due to structural similarity, 5-FU interferes with nucleoside metabolism and can also be incorporated into RNA and DNA [6], leading to cytotoxicity and cell death. However, the main anticancer mode of action is inhibition of the enzyme thymidylate synthase, which is essential in nucleotide synthesis (reviewed by [7]). The overall response rates for 5-FU-based chemotherapy for advanced CRC are around 15% [8, 9]. When combined with other anticancer drugs such as oxaliplatin, response rates improve to 40–50% [10, 11]. Despite progress in targeted therapy, 5-FU remains cornerstone of chemotherapy of CRC and other cancers. Although it has been widely used for almost 60 years, some of the mechanisms underlying its toxicity and resistance remain unclear and need further investigation.

We thus designed a panel of genes with pharmacogenomics records related to 5-FU and oxaliplatin resistance based on PharmGKB, DGIdb, DrugBank, GDSC (Genomics of Drug Sensitivity in Cancer), and COSMIC (Catalogue Of Somatic Mutations In Cancer) databases. Moreover, we included genes important for sensitivity to other drugs frequently used in CRC patients and major oncodrivers according to the latest tier 1 and 2 CGC and actionable genes in COSMIC. The panel was enriched with principal genes for CRC progression identified in recent whole exome sequencing studies [12, 13] and finally consisted of 558 genes for which the molecular probes have been designed in NimbleDesign. We performed a target enrichment sequencing of DNA from tumors and matched blood samples of CRC patients, and compared the results with patient prognosis stratified by adjuvant chemotherapy.

## Methods

### Patients

Paired samples of tumor tissue and blood were collected from 83 patients who were diagnosed with sporadic primary CRC tumors at various stages. All patients underwent surgery at the Department of Surgery of the University Hospital in Pilsen between 2015 and 2019. The clinical data were obtained from medical records and contained information about the age at diagnosis, sex, disease stage, tumor grade and location, surgery including resection margins, oncological treatment, recurrence or progression after surgery, and date of last control or death. Table 1 contains a summary demographic and clinical data of patients.

The overall survival (OS) was defined as the time elapsed between resection of a primary tumor and death from any cause or patient censoring. The recurrence-free survival (RFS) was defined as the time elapsed between the resection and recurrence of the tumor; death or last control in remission were censored events.

### DNA isolation and quantification

DNA from fresh-frozen tissue samples of primary tumors was isolated using the DNeasy Blood and Tissue Kit (Qiagen, Hilden, Germany) according to the manufacturer’s instructions. DNA was eluted into 200 µL of AE buffer, divided into triplicates, and stored at -20 °C until further use. DNA from the whole blood samples collected during the surgery was isolated using BioSprint 15 DNA Blood Kit (Qiagen) combined with an automatized KingFisher mL Purification System (ThermoFisher Scientific, Waltham, MA, USA) using magnetic particles. We modified the manufacturer’s protocol to isolate DNA from 1 mL of human whole blood instead of referenced 100–300 µL. In the modified protocol, we first pipette 90 µL of protease, add 1 mL of whole blood, and vortex sample for 15 s. Then we incubate the sample at 70 °C for 10 min, add 0.9 mL of isopropanol, and shortly spin for 1 min at 1,000 rpm. The obtained lysate is then applied into each column of the KingFisher mL Purification System, other buffers are applied in their respective positions, and the machine is initialized. In the end, isolated DNA is eluted into 300 µL of AE buffer and stored at -20 °C until further use. For DNA quantification, we use Qubit 3.0 Fluorometer and dsDNA Broad Range Assay Kit (both ThermoFisher Scientific).

### Target capture panel in silico analysis, design, and synthesis

We searched several databases, e.g., PharmGKB (www.pharmgkb.org), DGIdb (http://dgidb.genome.wustl.edu/), DrugBank (www.drugbank.ca), GDSC (https://www.cancerrxgene.org/), and COSMIC v81 (http://cancer.sanger.ac.uk/cosmic) for interactions between human genome and sensitivity to 5-FU and oxaliplatin. Through a three-phase search, we prioritized 264 genes. Firstly, mutations in all screened cell line models (*n* = 968) in the GDSC have been crosschecked together with sensitivity data for 5-FU and oxaliplatin (IC_50_ and area under curve, AUC). The most frequently mutated genes in either the most sensitive or resistant cell line models (each category of “resistant” or “sensitive” cell lines had 20 cell lines with 10 of CRC origin) have been selected. Genes with more than 5% mutations of the total observed in half or more cell lines in each category (to exclude multiple single gene mutations in a single cell line) have been considered as general marks of sensitivity/resistance to drugs and passed to the second phase (*n* = 745 for sensitive and *n* = 101 for resistant). In the second phase, genes were checked by the HGNC server (http://www.genenames.org/) for compliance with HuGo nomenclature and merged (*n* = 302 genes). In the third phase, the COSMIC v81 tool was used for the identification of genes somatically mutated in human CRC (*n* = 715 samples) with more than 5% frequency (*n* = 780 genes) as well as genes without mutations in these samples (*n* = 2767 genes). After gene nomenclature check, both databases (cell line and human tumor mutations) have been crosschecked, and the final list of genes fulfilling these criteria: i/ mutated in 50% or more of sensitive or resistant cell lines, ii/ mutated in human CRC at more than 5% frequency, iii/ absent in the list of not mutated genes in CRC has been produced (*n* = 264 genes). These genes have been analyzed for molecular function, cellular complement, biological process, and pathway context by the Panther database (http://pantherdb.org/). Binding (GO:0005488), catalytic activity (GO:0003824), structural molecule activity (GO:0005198), receptor activity (GO:0004872), and transporter activity (GO:0005215) comprised more than 85% of cellular functions affected by mutations in both 5-FU sensitive/resistant cell lines and CRC tumors. The list of genes was enriched with an additional 294 genes listed among CGC tier 1 and 2 and “Actionable” in COSMIC v81, together with principal genes for CRC progression or from the latest whole exome sequencing studies [12, 13]. The final list of genes for gene variability consists of 558 genes.

The panel was designed in NimbleDesign (Nimblegen, Roche, Basel, Switzerland). All possible transcript variants and RefSeq, Ensembl, and UCSC databases were used to select chromosomal coordinates in genome build hg19. Probes were selected in moderate stringency (preferred close matches 3, maximum close matches 20) and manufactured using NimbleGen SeqCap EZ Choice format (Roche). For the complete list of genes, see **Supplementary Table ****S1**.

### Library preparation and whole exome sequencing

Sequencing libraries were prepared using the NEBNext Ultra II FS DNA Library Prep Kit for Illumina (New England Biolabs, Ipswich, MA, USA) according to the manufacturer’s instructions. Briefly, 100 ng of DNA was enzymatically digested, adaptors were ligated and adaptor-ligated DNA was enriched using 7–8 PCR cycles. The quality of prepared libraries was controlled using TapeStation 2200 (Agilent, Santa Clara, CA, USA) and libraries were quantified using Qubit 3.0 Fluorimeter and dsDNA High Sensitivity Assay Kit (ThermoFisher Scientific).

Samples were multiplexed in pooled libraries containing 1000 ng DNA libraries derived either from 11 samples of tumor tissue DNA or 22 samples of blood DNA and hybridized with custom probes using standard NimbleGen SeqCap EZ Library LR protocol (Roche) with the following modifications of hybridization and post-capture PCR steps. In the hybridization reaction, 13.4 µl of Kapa Universal Enhancing Oligos (Roche) were added to the bead-bound DNA Sample instead of SeqCap HE Universal and Index oligos. After performing the capture reaction, the libraries were amplified using Primer 1: 5’-AATGATACGGCGACCACCGAGATCTACAC-3’ and Primer 2: 5’-CAAGCAGAAGACGGCATACGAGAT-3’. For the amplification, Ultra II Q5 PCR master mix (NEB) was used in a total reaction volume of 100 µl. Captured sequences were amplified using 13 PCR cycles. For assessment of the quality and quantity of final libraries, TapeStation 2200 (Agilent) and Qubit 3.0 Fluorometer with dsDNA High Sensitivity Kit (ThermoFisher Scientific), respectively, were used. Samples were pooled into the final pool in a non-equimolar fashion (tumors/blood ratio 3:1) and the final pool was sequenced on the NovaSeq 6000 platform (Illumina, San Diego, CA, USA) using 150 bp pair-end sequencing on one lane of the S4 flow cell.

### Bioinformatic analysis

The pipeline used for bioinformatic processing of raw data has been described elsewhere in detail [13]. Here, we describe the procedure only briefly with relevant references. Adapter and low-quality base trimming was done by Trimmomatic. Reads were aligned to the hg38 human reference genome sequence using Burrows-Wheeler Aligner v0.7.17-r1188 (BWA, Cambridge, UK) with the BWA-maximal exact matches (MEM) algorithm [14]. Base recalibration was done using the Genome Analysis Toolkit v.4.3.0.0 (GATK) (Broad Institute, Cambridge, UK) according to GATK Best Practices [15]. Duplicate reads were identified by MarkDuplicates (Picard). Identification of somatic variants and short indels was performed in paired tumor-normal samples using Mutect2 (GATK). Detected variants were filtered using FilterMutectCalls (GATK) and only variants passing all filters (i.e., somatic variants with filter status PASS) were considered. Variants were filtered on min. variant allele frequency (VAF) 5% and supported by min. three reads. Germline variants were called using HaplotypeCaller and variant recalibration was done by VariantRecalibrator (both GATK).

Annotation was performed in Variant Effect Predictor (VEP) v.108 [16], which assigned one of the following values to each variant: LOW, MODIFIER, MODERATE (missense, in-frame deletions, and insertions), or HIGH (nonsense, frameshift, splice site, transcription start site) functional effect. Variants with HIGH and MODERATE predicted effects were evaluated for clinical associations. Visualization was performed in Maftools [17] or ComplexHeatmap [18] (both R/Bioconductor).

CNVs were detected with CNVkit v0.9.9 [19] and VarDict tool v1.8.3 [20]. Tumor purity was estimated using PureCN v.2.0.2 (R/Bioconductor). Significant calls were assessed based on the average read depth a log2 ratio values and B-allele frequencies (BAF) of individual segments. Assuming a theoretical clonal fraction (tumor purity) of 70%, a deletion should have log2 ratio < -0.278 and BAF between 0.325 and 0.675; a duplication should have a log2 ratio > 0.233 and BAF between 0.442 and 0.558. All called segments that contained less than three bins or did not show a statistically significant difference of log2 ratios compared to reference values (*p* < 0.05 by the Student’s t-test) were excluded.

Microsatellite instability was detected using MSI-sensor2 v0.1 (https://github.com/niu-lab/msisensor2) based on the published 20% threshold [21]. For the detection of indels in homopolymer regions per Mb as a surrogate marker of mismatch repair deficiency (MMR-D) [22], the homopolymer regions were identified by Vcfpolyx (part of Jvarkit, https://github.com/lindenb/jvarkit) and were defined as genomic regions with more than four repeat bases. Samples with > 1.5 indels in homopolymer regions per Mb were considered MMR-D [23].

### External validation

The validation set was downloaded from the USCS Xena Browser [24]. GDA TCGA datasets COAD and READ were merged and only variants in candidate genes with predicted HIGH or MODERATE effect (see Material and Methods section Bioinformatic analysis) were used in statistical analyses. Only primary tumors with adenocarcinoma diagnosis and patients with complete survival follow-up were selected.

### Statistical analyses

Differential analyses were performed in patient subgroups stratified by main clinical data (age, sex, stage, grade, tumor localization, and adjuvant chemotherapy response – remission vs. progression). Analyses of differences in the number of variants or their functional classification between groups of patients divided by the above parameters were performed using Fisher’s exact test. Differences in TMB and CNVs between patients stratified by the above data were compared using the Kruskal-Wallis test and correlations of continuous data such as patient age, CNV size, or CNV counts were assessed using Spearman’s rho test. For the associations of germline variants with survival, Plink v1.9 was used to perform chi-square allelic tests with Monte-Carlo max(T) permutation test. Patients were divided according to RFS ≤ 3 years vs. > 3 years, which is an appropriate end point for adjuvant treatment of regimens based on 5-FU [25]. Manhattan plot was generated using package qqman (R/CRAN). Survival functions for groups of patients divided by genetic data, eventually stratified by chemotherapy, were plotted using the Kaplan-Meier method, and significance was calculated by the Breslow test. All continuous variables were divided by the median. The Benjamini-Hochberg false discovery rate (B-H FDR) test was used for the correction of multiple testing [26] and adjusted p-value (p_adj_) < 0.05 was considered significant. For associations between single genes and clinical data, unadjusted p-values (p_crude_) are provided to indicate trends. A two-sided p_crude_ <0.05 was used for selection of genes for testing their combinations and associations with p_adj_ < 0.05 were subjected to external validation where possible. All statistical analyses were performed in the SPSS v16 program (SPSS Inc., Chicago, IL, USA) or R package survminer/CRAN (R version 4.3.3).

## Results

### Clinical characteristics of the patients

The main characteristics of the patients are summarized in Table 1. The median age of patients at the time of CRC diagnosis is 68 years (range 42–87) and the study group comprises slightly more men (53%) than women (47%). The unequal sex distribution is not intentional and corresponds with the reported higher incidence of CRC in men [1]. The majority of patients have stage II or III disease (40% resp. 47%) and tumors localized in the left colon (60%). One patient at stage IV was excluded from survival analyses and the rest of the analyses included the group of patients with stage III. The vast majority of surgical tumor removal procedures were evaluated as R0, i.e. tumor-free resection margins. About 62% of patients were administered 5-FU-based systemic adjuvant chemotherapy with or without oxaliplatin and the rest of the patients did not receive any chemotherapy due to either poor performance status or lack of risk factors for stage II. The median follow-up is 48 months. RFS of the patients is significantly associated with disease stage (*p* = 0.045) and the presence of regional lymph node metastases (*p* = 0.021). OS is associated only with the latter (*p* = 0.045) (**Supplementary Fig. ****S1**).

**Table 1:** Clinical characteristics of the patients

Parameters	Number of patients	Percentage
Age at diagnosis	83	100
Median ± SD (years)	68.0 ± 9.2	
Sex		
Male	44	53
Female	39	47
Tumor extent (pT)		
pT1	3	4
pT2	12	14
pT3	61	74
pT4	7	8
Regional lymph node metastasis (pN)		
pN0	43	52
pN1	25	30
pN2	15	18
Distant metastasis (pM)		
Absent	82	99
Present	1	1
Stage		
I	10	12
IIA/IIB	33	40
IIIA/IIIB/IIIC	39	47
IV	1	1
Histologic grade (G)		
G1	18	22
G2	55	67
G3	9	11
Gx	1	-
Tumor sidedness		
Right colon	33	40
Left colon	50	60
Resection margins (R)		
R0	80	96
R1	3	3
Adjuvant chemotherapy		
5-fluorouracil + leucovorin^*^	27	35
FOLFOX^#^	21	27
Not administered	30	38
Data not available	5	––
Remission after adjuvant chemotherapy		
Stable	42	87
Progression	6	13
Not applicable	35	––
Overall survival	82	95
Median 95% confidence interval (months)	48 (37.7 - 58.3)	––

^*^deGramont regimen.

^#^5-fluorouracil with oxaliplatin and leucovorin.

### Somatic profile of tumor samples

**Fig. 1:**
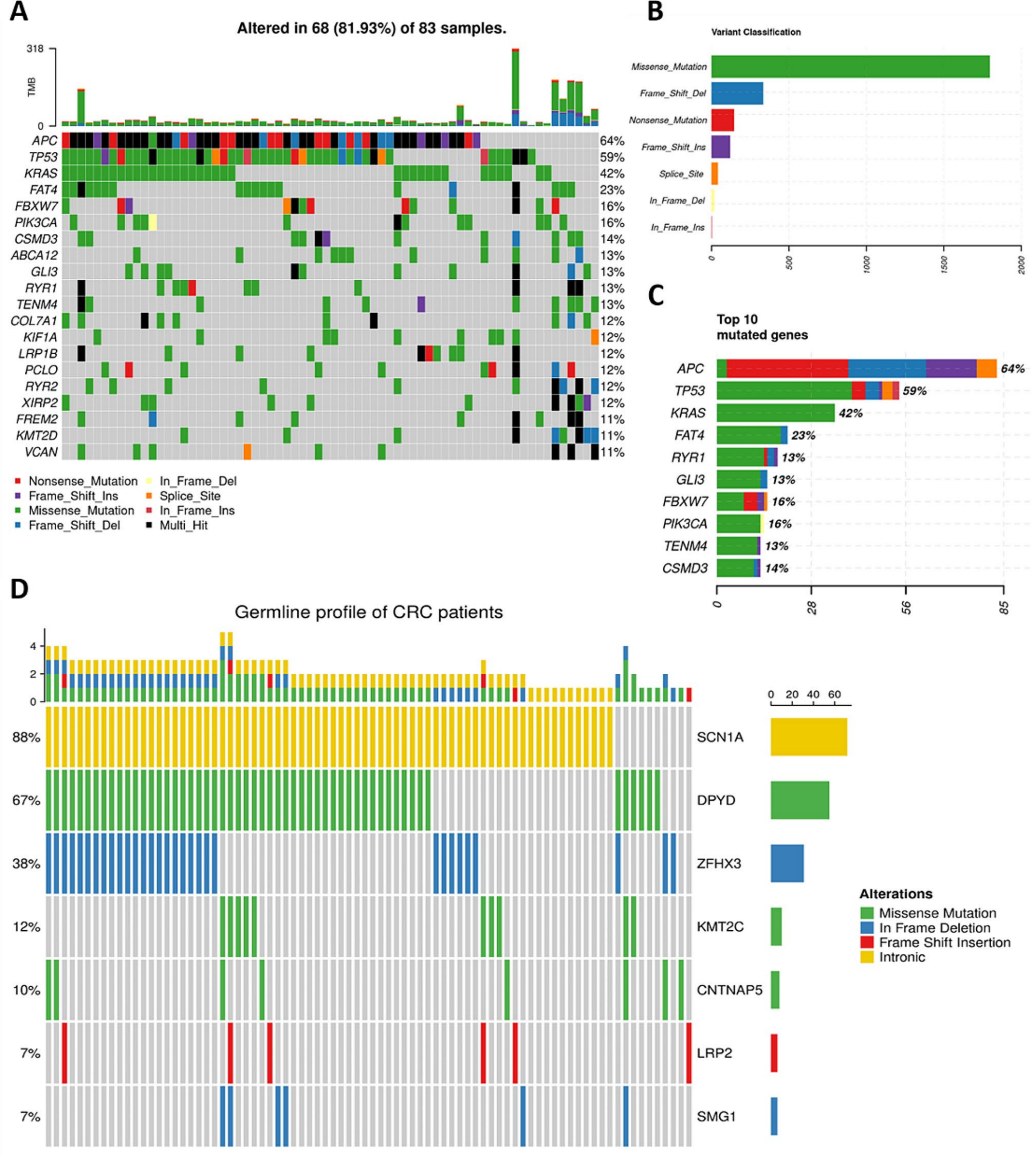
Oncoplot of somatic and germline variability of targeted gene panel in CRC patients. (**a**) Plot of top 20 somatically most mutated genes. (**b**) The classification of variants according to their functional effect (missense, frameshift deletion/insertion, nonsense, splice site, or in-frame deletion/insertion mutations). The most prevalent variants were missense. (c) Overall distribution of variants in top 10 genes with most somatic mutations. (d) Genes with germline variants listed in ClinVar or InterVar databases

The median number of detected variants per tumor sample was 18.5 (ranging from 0 to 318). The median amount of somatic variants fulfilling the functional classification HIGH (see Materials and Methods) per sample was 4 (0–79) and for MODERATE 14.5 (0–239). *APC, TP53*, and *KRAS* were the most frequently mutated genes (64%, 59%, and 42% of mutated samples, respectively). Additionally, *FAT4* (23%), *FBXW7*, and *PIK3CA* (16% for both) belong to the most mutated genes (Fig. 1a, c and **Supplementary Table ****S2**). The most common class of somatic variants was the missense mutation (Fig. 1b). The median TMB per Mb was 3.6 (0–62.3) and seven patients were classified as MSI-high and MMR-D. The median CNV size was 15.48 Mbp (0.048–36.24). The mutation summary for all samples is in **Supplementary Table****S3**.

### Germline profile of CRC patients

The median number of all detected germline variants per sample was 326 (ranging from 310 to 355) and that of variants with the HIGH predicted effect was 25 (19–30). At least 5% frequency for the sum of all variants with the HIGH effect was observed in 55 genes. Out of these, pathogenic or drug response-connected variants were called by ClinVar or InterVar for *SCN1A* (88% of patients), *DPYD* (67%), *ZFHX3* (38%) *KMT2C* (12%), *CNTNAP5* (10%), *LRP2* (7%) and *SMG1* (7%) (Fig. 1d). *SCN1A* (rs3812718), *ZFHX3* (rs372909378), *CNTNAP5* (rs17727261), *LRP2* (rs80338754), and *SMG1* (rs781029159) were unique polymorphisms, while for *KMT2C* (rs199504848 and rs763762478) and *DPYD* (97,883,329, 97,515,839, 97,699,535, 97,305,364) multiple polymorphic loci were found (**Supplementary Table****S4**).

### Clinical associations of mutational profiles

For all analyses, variants with HIGH or MODERATE predicted functional effects counted together were used. We first analyzed associations between individual gene mutation frequencies or functional classification and clinical data including survival.

Patients with a higher risk of progression - in stages III or IV (*n* = 40) have more frequent mutations in *APC* (p_crude_=0.036, Fisher’s exact test), *TP53* (p_crude_=0.040), or *KRAS* (p_crude_=0.048) than those in less advanced stages I or II. For *KRAS*, we also found an increasing trend in mutation counts with grade (G1 < G2 < G3, p_crude_=0.030). In the case of *APC*, the association with disease stage was even more pronounced for frameshift type of mutations (p_crude_=0.015) and *KRAS* specifically for the 12D mutation (p_crude_=0.012 for stage and p_crude_=0.027 for grade), all in the same direction. The response to adjuvant therapy was worse in carriers of frameshift mutations in *APC* (p_crude_=0.008) or 12D variant in *KRAS* (p_crude_=0.005) (Table 2). However, none of these associations passed the FDR adjustment for multiple testing (p_adj_>0.05).

**Table 2:** Associations of somatic variants in individual genes with clinical data

Characteristics	Wild type*	Mutant*	*p*_crude_/*p*_adj_
	*APC* any variant		
Stage I/II	18	25	0.036/0.075
Stage III/IV	8	32	
	*APC* frameshift variants		
Stage I/II	36	7	0.015/0.056
Stage III/IV	23	17	
	*TP53* any variant		
Stage I/II	21	22	0.040/0.075
Stage III/IV	10	30	
	*KRAS* any variant		
Stage I/II	29	14	0.048/0.080
Stage III/IV	18	22	
	*KRAS-12D*		
Stage I/II	41	2	0.012/0.056
Stage III/IV	30	10	
	*KRAS* any variant		
Grade 1	15	3	0.030/0.075
Grade 2	27	28	
Grade 3	4	5	
	*KRAS-12D*		
Grade 1	16	2	0.027/0.075
Grade 2	49	6	
Grade 3	5	4	
	*APC* frameshift variants		
Stable response	32	10	0.008/0.056
Progression	1	5	
	*KRAS-12D*		
Stable response	38	4	0.005/0.056
Progression	2	4	

*Numbers of patients presented

Although the survival analysis did not offer a significant relationship (p_crude_/p_adj_>0.05), the trends were very clear. Patients with shorter RFS more often have frameshift mutations in *APC* (p_crude_=0.064, **Supplementary Fig. ****S2****a**) or carry the *KRAS*-*12D* variant (p_crude_=0.057, **Supplementary Fig. ****S2****b**). The relationships with OS were less pronounced, although in the same direction (p_crude_=0.180 for *APC* and p_crude_=0.200 for *KRAS*-*12D*) (**Supplementary Fig. ****S2****c, d**). Neither the frequency nor the functional classification of somatic mutations in *TP53* had predictive or prognostic significance. When analyzing combinations based on co-mutated *APC, KRAS*, and *TP53*, the combination *TP53* co-mutated with *KRAS* codons 12 or 13 and more specifically subset of *KRAS-12D* with *TP53* co-mutated had worsened OS (p_crude_=0.024 and p_crude_=0.047, respectively, **Supplementary Fig. ****S2****e, f**), but not RFS (p_crude_=0.420 and p_crude_=0.078, respectively). None of these associations passed the FDR adjustment for multiple testing (p_adj_>0.05). Several patients had all three genes co-mutated, based on *APC* frameshift or nonsense type of mutations (*n* = 29), but this combination did not significantly modify their survival (p_crude_>0.05).

As for other genes, we found several relationships between mutation spectra and patient survival among genes mutated in at least 10% of samples (*n* = 45 genes, **Supplementary Table ****S2**). The rest of genes was not analyzed in a single gene mode due to small numbers of patients in the compared subgroups. Mutations in *ANK2* and *SACS* were associated with shorter RFS (p_crude_=0.021 and p_crude_=0.014, respectively) and those in *ABCA13, ANK2, COL7A1, NAV3*, and *UNC80* with shorter OS (p_crude_<0.001, p_crude_<0.001, p_crude_=0.002, p_crude_=0.005, and p_crude_=0.035, respectively) regardless of treatment. Interestingly, *KMT2D* showed an inverse relationship to OS, i.e. shorter survival in patients without variants (p_crude_=0.050) (**Supplementary Fig.****S3**a-h).

In untreated patients only (*n* = 29), no relationship to RFS was found, but carriage of somatic mutations in *ABCA13, ANK2, COL7A1, FLG, GLI3*, and *UNC80* was associated with OS (p_crude_<0.001, p_crude_=0.012, p_crude_=0.046, p_crude_=0.006, p_crude_=0.012, and p_crude_<0.001, respectively) (**Supplementary Fig.****S4**a-f).

No association with RFS was also found in patients treated with adjuvant regimens of chemotherapy (*n* = 47, one patient with stage IV excluded from survival analyses). On the other hand, we found many relationships with OS, namely for poor OS and carriage of mutations in *ABCA13, ANK2, COL6A3, COL7A1, LRP1B, NAV3, RYR1, RYR3, TCHH*, and *TENM4* (p_crude_=0.001, p_crude_=0.029, p_crude_=0.002, p_crude_=0.004, p_crude_=0.003, p_crude_=0.036, p_crude_=0.043, p_crude_=0.027, p_crude_=0.037, and p_crude_=0.015, respectively) (**Supplementary Fig.****S5**a-j).

**Fig. 2:**
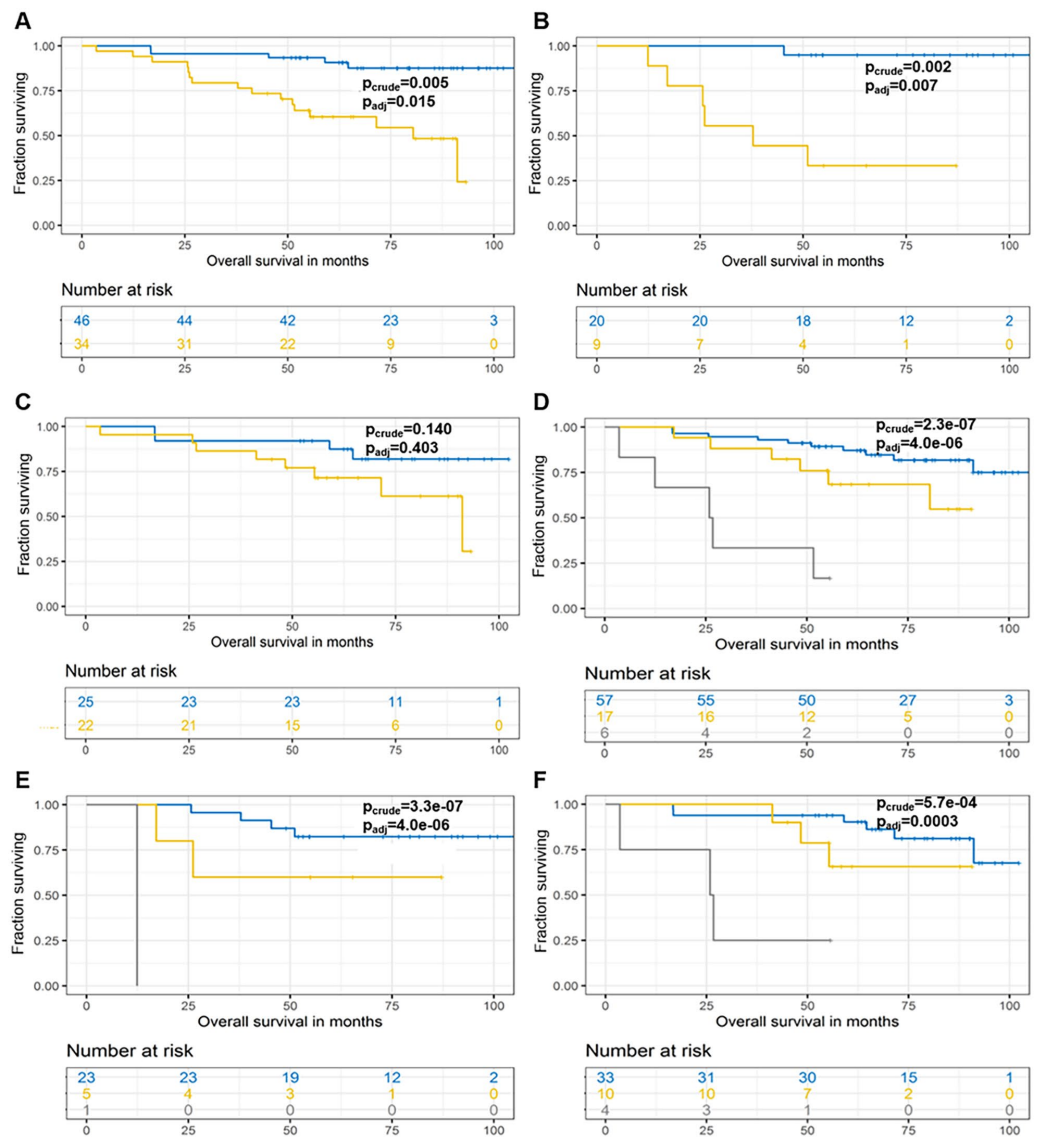
Kaplan-Meier plots of patient survival stratified by the carriage of somatic variants in *ABCA13*-*ANK2-COL7A1* or *ABCA13-ANK2-COL7A1*-*NAV3*-*UNC80*. OS analysis of somatic variants in *ABCA13, ANK2, COL7A1*, *NAV3*, or *UNC80* in all (**a**), untreated (**b**), and adjuvantly treated (**c**) patients. OS analysis of somatic variants in *ABCA13*, *ANK2*, or *COL7A1* in all (**d**), untreated (**e**), and adjuvantly treated (**f**) patients. Blue line represents patients without mutations, the yellow line patients carrying mutations in single gene, and the grey line those with mutations in more than one gene (where applicable)

From the above results, it was apparent that variants in *ABCA13, ANK2*, and *COL7A1* carry prognostic information regardless of whether adjuvant oncological treatment was administered or the patient was just discharged. Furthermore, *FLG, GLI3*, and *UNC80* appear to be prognostic in treatment-naïve patients, whereas *COL6A3, LRP1B, NAV3, RYR1, RYR3, TCHH*, and *TENM4* in those treated with adjuvant chemotherapy. We therefore grouped all genes with p_crude_<0.05 for further analysis. In these analyses, we applied the FDR adjustment for multiple testing to all results. OS of patients with mutations in *ABCA13, ANK2, COL7A1*, *NAV3*, or *UNC80* grouped was highly significantly worse than in patients who did not carry mutations in any of these five genes (p_adj_=0.015, Fig. 2a). This five-gene signature was prognostic, in the same direction, also in untreated (p_adj_=0.007, Fig. 2b) but not in adjuvantly treated (p_crude_=0.140, Fig. 2c) patients. A combination of three genes *ABCA13*, *ANK2*, or *COL7A1* had the same effect (all p_adj_<0.001, Fig. 2d-f), but combination of 10 genes (*ABCA13, ANK2, COL7A1, COL6A3, LRP1B, NAV3, RYR1, RYR3, TCHH*, and *TENM4*) was not significant (all p_crude_>0.05) indicating that associations are gene-selective irrespective of just general mutation load.

**Fig. 3:**
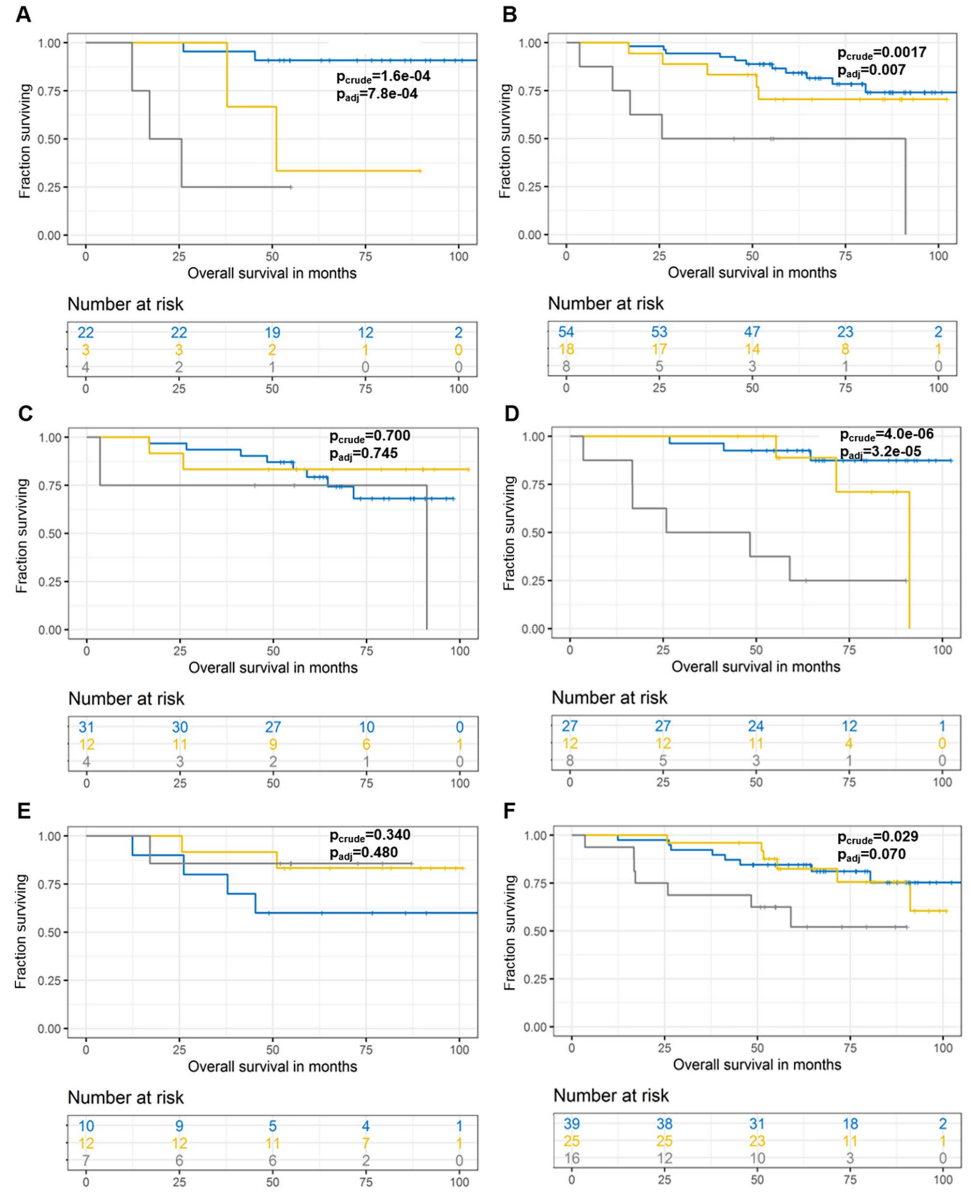
Kaplan-Meier plots of patient survival stratified by the carriage of somatic variants in *FLG-GLI3-UNC80* or *COL6A3-LRP1B-NAV3-RYR1-RYR3-TCHH*-*TENM4*. OS analysis of somatic variants in *FLG, GLI3*, or *UNC80* in untreated (**a**), all (**b**), and adjuvantly treated (**c**) patients. OS analysis of somatic variants in *COL6A3, LRP1B, NAV3, RYR1, RYR3, TCHH*, or *TENM4* in adjuvantly treated (**d**), untreated (**e**), and all (**f**) patients. Blue line represents patients without mutations, the yellow line patients carrying mutations in single gene, and the grey line those with mutations in more than one gene (where applicable)

Moreover, the carriage of mutations in any of genes from combination of *FLG, GLI3*, or *UNC80* was prognostic for worse OS in untreated patients (p_adj_<0.001, Fig. 3a), while less significantly also in all patients (p_adj_=0.007, Fig. 3b), but not at all in treated ones (p_crude_=0.700, Fig. 3c). Finally, worse OS was observed in adjuvantly treated patients with mutations in any of *COL6A3, LRP1B, NAV3, RYR1, RYR3, TCHH*, or *TENM4* and it was even worse in carriers of multiple gene mutations (p_adj_<0.001, Fig. 3d). On the contrary, this gene combination was not prognostic in terms of OS for untreated (p_crude_=0.340, Fig. 3e) and weakly significant before FDR adjustment in all patients (p_crude_=0.025/p_adj_=0.070, Fig. 3f). None of the above combinations was prognostic for RFS (p_crude_>0.05).

We further divided the gene set according to the occurrence of mutations in oncodriver pathways identified in our previous exome studies as associated with CRC progression (MYC, Hippo, Notch, RTK-RAS, PI3K, HRR, and the immunogenic signature ICB1) [19] (**Supplementary Table****S5**). Although the gene panel was less informative on the complete pathway level as opposed to the exome, the gene selection was broad enough and included the majority of principal genes from the mentioned pathways, as can be judged from the resulting associations with clinical data. Patients with mutations in the MYC, PI3K, RTK-RAS, and ICB1 pathways had more often regionally advanced stage III or generalized IV than locally advanced stages I or II (p_crude_=0.004, p_crude_=0.030, p_crude_=0.003, and p_crude_=0.033, respectively, Table 3) although these associations did not pass the FDR adjustment to multiple testing (p_crude_>0.05). Despite these associations, we did not observe a prognostic significance for any of the observed pathways. Hippo, Notch, or HRR (homologous recombination repair) pathways did not associate with any of the clinical characteristics (p_crude_>0.05).

**Table 3:** Associations of somatic variants in oncodriver pathways with clinical data

Characteristics	Wild type	Mutant	*p*_crude_/*p*_adj_
	MYC pathway		
Stage I/II	43	0	0.004/0.056
Stage III/IV	33	7	
	PI3K pathway		
Stage I/II	35	8	0.030/0.168
Stage III/IV	23	17	
	ICB1 gene set		
Stage I/II	15	28	0.003/0.056
Stage III/IV	3	37	
	RTK-RAS pathway		
Stage I/II	11	7	0.033/0.196
Stage III/IV	16	39	

*Numbers of patients presented

The MSI-high status, TMB divided by median, CNV size, or individual copy number alteration types divided by median showed no clinical associations and had no apparent prognostic role (*p* > 0.05).

**Fig. 4:**
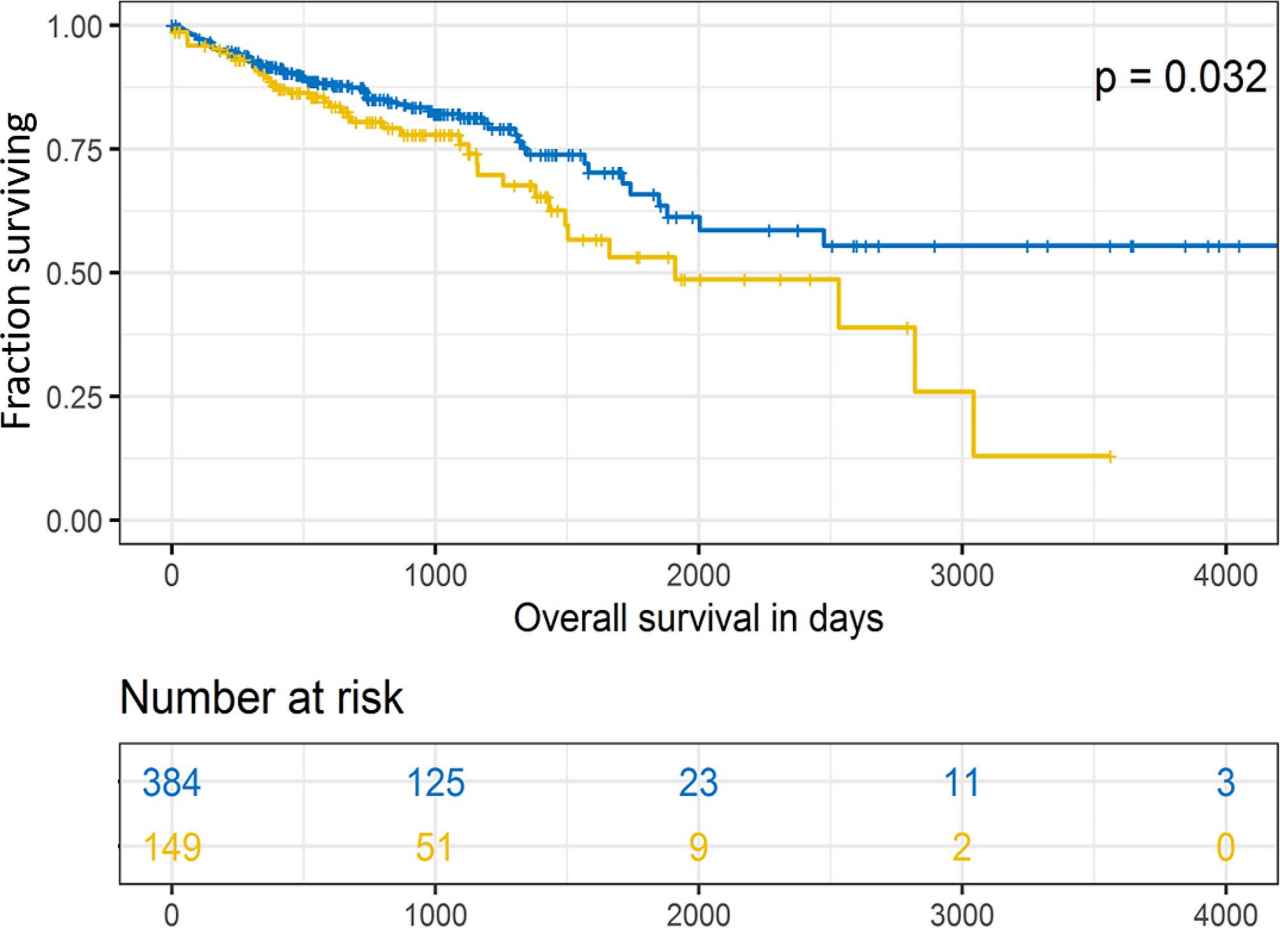
Kaplan-Meier plot of external validation of patient survival stratified by the carriage of somatic variants in *ABCA13-ANK2-COL7A1*-*NAV3*-*UNC80*. Blue line represents patients without mutations and the yellow line patients carrying mutations in any of the studied genes

We validated the observed prognostic associations of somatic variants with OS using the external dataset TCGA COAD-READ (specification in Materials and methods). We confirmed the association of *ABCA13, ANK2, COL7A1*, *NAV3*, or *UNC80* with OS in all patients regardless of whether patients were treated with adjuvant chemotherapy or not (*p* = 0.032, Fig. 4). Despite we could still see the trend of longer survival of non-mutated patients the mutation dosage did not yield significant results (**Supplementary Fig.****S6**d). Similarly, the rest of the observed associations showed a clear trend although p-values were statistically insignificant. Results of external validation are presented in **Supplementary Fig.****S6**a-l.

**Fig. 5:**
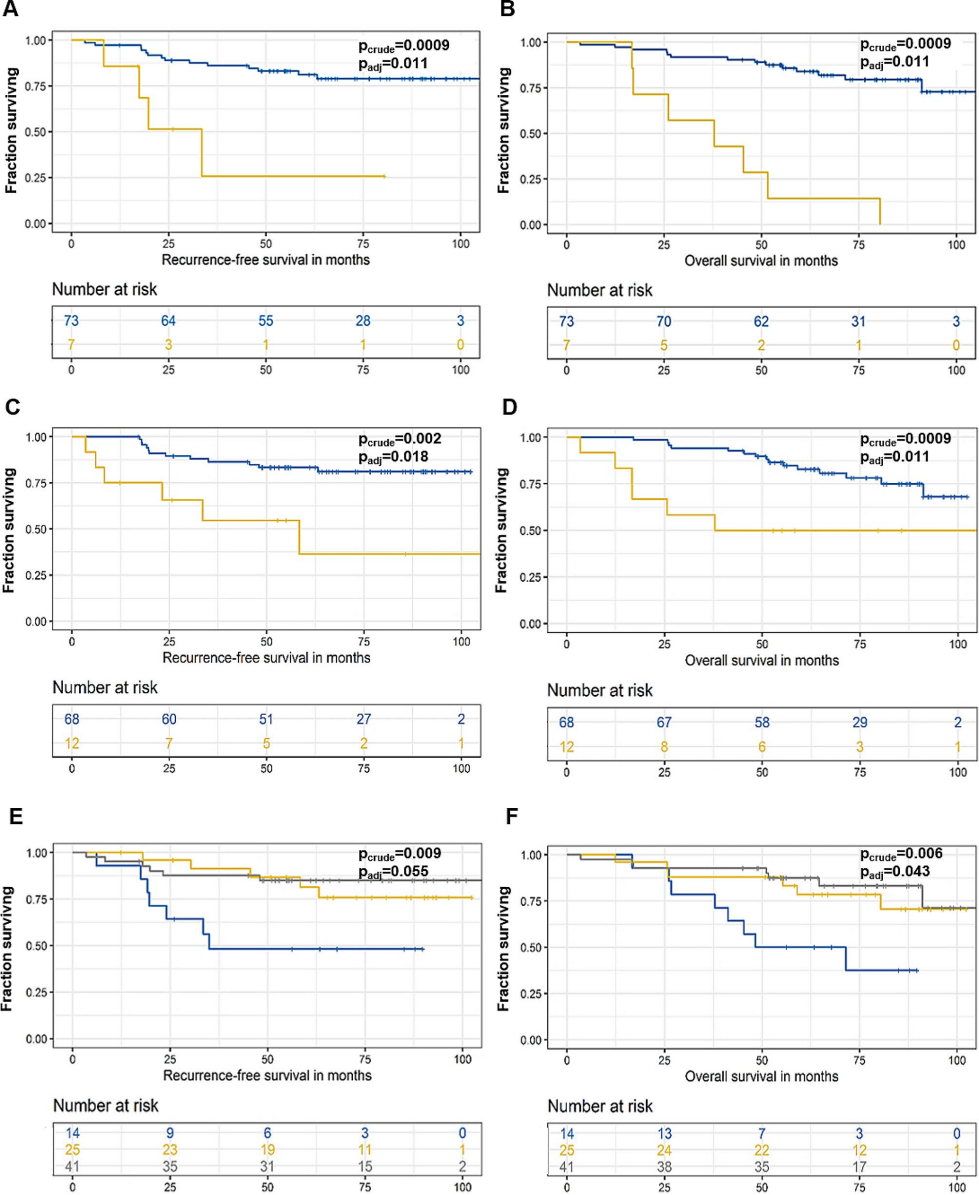
Kaplan-Meier plots of patient survival stratified by the carriage of germline variants in individual genes. RFS analysis of germline variants in *NFACS*- rs72753407 (**a**), *ERBB4*-rs34621071 (**c**), and *RIF1*-rs2444274 (**e**). OS analysis of germline variants in *NFACS*- rs72753407 (**b**), *ERBB4*-rs34621071 (**d**), and *RIF1*-rs2444274 (**f**). Blue line represents patients carrying the wild-type and yellow line carriers of heterozygous genotype. Grey line represents variant genotype carriers in (e) and (f) plots

From all germline variants, we tested those having more than 5% frequency and either record in ClinVar or InterVar databases (11 variants in 7 genes) or indication of an association with RFS divided by three years on Manhattan plot (7 variants in 4 genes, **Supplementary Fig.****S7**). Of these, carriers of heterozygous genotype rs72753407 (intron variant) in *NFACS* had significantly poorer RFS and OS (p_crude_<0.001/p_adj_=0.011 and p_crude_<0.001/p_adj_=0.011, respectively) than wild-type patients (Fig. 5a, b). Additionally, patients carrying heterozygous genotype rs34621071 (intron) in *ERBB4* had significantly worse OS and insignificant trend towards worse RFS, after FDR adjustment compared to wild-type carriers (p_crude_=0.002/p_adj_=0.018 for RFS and p_crude_<0.001/p_adj_=0.011 for OS, Fig. 5c, d). Although patients with wild-type for rs2444274 (intron) in *RIF1* had worse RFS and OS than carriers of heterozygous or variant genotypes, these associations did not pass the FDR adjustment (p_crude_=0.009/p_adj_=0.054 for RFS) or remained borderline significant (p_crude_=0.006/p_adj_=0.043 for OS) (Fig. 5e, f). The rest of variants identified by the Manhattan plot (*NFASC*-rs2595959, *RIF1*-rs16830036 and rs16830047, and *SYNE1*-rs9479265) were not significant (p_crude_>0.05).

No pathogenic or drug response-connected variants according to ClinVar or InterVar (*CNTNAP5-*rs17727261, *DPYD-*rs1801265, rs1801160, rs1801159, and rs2297595, *LRP2*-rs80338754, *KMT2C-*rs199504848 and rs763762478, *SCN1A-*rs3812718, *SMG1-*rs781029159, and *ZFHX3-*rs372909378) were associated with survival of patients (p_crude_>0.05).

## Discussion

In this study, we designed, with the help of *in silico* tools, a panel of genes with in vitro records related to sensitivity and resistance of drugs (5-FU and oxaliplatin) most frequently used for treatment of stage II or III CRC patients. Major oncodrivers, actionable genes, and genes identified in recent whole exome sequencing studies [12, 13] were also included to enrich the genetic landscape of patients. The study design enabled the assessment of the contribution of selected genes on both somatic and germline levels.

In general, our study identified specific gene sets bearing prognostic relevance for all patients and sets composed of different genes for patients stratified by systemic adjuvant chemotherapy. When considering the whole sample set, patients with somatic mutations in any of the genes *ANK2*, *ABCA13*, or *COL7A1* had highly significantly worse OS than non-carriers. In untreated patients, somatic mutations in *FLG*, *GLI3*, or *UNC80* were prognostic towards poor OS, and the same role was seen for somatic mutations in *COL6A3*, *LRP1B*, *NAV3*, *RYR1*, *RYR3*, *TCHH*, or *TENM4* in patients treated with adjuvant therapy based on 5-FU or FOLFOX regimens. Highly interestingly, none of these genes except *LRP1B* is listed among oncodrivers in CGC or the list of actionable genes in COSMIC. *LRP1B* is considered a tumor suppressor and its somatic mutability was recently associated with improved OS of CRC patients [27], higher tumor mutation burden and tumor neoantigen burden connected with benefit from PD-1 blockade in MMR-proficient rectal carcinomas [28]. Thus, our study confirms its relevance for CRC.

Taken together, our study demonstrates the power of *in silico* mining of in vitro data for gene prioritization further empowered by available human tumor data. We identified 13 previously unreported genes with prognostic relevance for CRC. The decision tree for patients with stage II CRC towards systemic adjuvant chemotherapy is still not completely resolved issue. It relies mostly on clinical signs such as pT4 disease, inadequately sampled lymph nodes, the presence of lymphovascular or perineural invasion, poorly differentiated or undifferentiated tumors, positive surgical margins, and eventually high preoperative serum level of tumor markers, mismatch repair deficiency (dMMR), or microsatellite instability-high (MSI-H) status [4, 29]. Nevertheless, there are no established genetic factors in the current therapy selection or patient prognosis estimation processes and our study suggests several candidates with already existing functional in vitro data.

Additionally, we speculated whether the prognostic role may be just a proxy to overall tumor mutational load and therefore we evaluated survival for all, untreated, or adjuvantly treated patients divided by the median count of all somatic mutations with HIGH or MODERATE predicted functional effect (defined in Materials and methods). Both this analysis and evaluation of all 13 genes mentioned above grouped together were non-significant. Thus, the observed associations cannot be simply attributed to the overall mutational rate, but rather to mutations in specific gene sets. Moreover, high observed significance after the FDR adjustment for multiple testing for a given gene set in the specific group or subgroup of patients and the lack of it in the other groups/subgroups suggest that these associations are rather causative and not just correlative or by chance results.

More importantly, the main oncodrivers in CRC, i.e., *APC*, *KRAS*, or *TP53* had no prognostic role evaluated either separately as single genes or combined. For *KRAS*, we evaluated also carriage of mutations 12D, or in codon 12 as an additional factor. For *TP53* and *APC* we divided patients according to functional status of mutations. Except for associations with clinical factors such as disease stage or grade, no prognostic value was found in contrast with data from recent whole exome sequencing of hepatic metastatic loci (12 for *KRAS*, 13 for *KRAS*-12D). Interestingly, patients with progression after adjuvant chemotherapy had more frequent *KRAS-12D* or *APC* frameshift mutations compared to patients in remission. The sample size and frequency of somatic mutations in these genes allowed also the evaluation of co-mutation effects. Neither the frequently reported for other cancers [30, 31], the *KRAS-TP53* co-mutation with dismal prognostic role, nor the other combinations, including the *APC-KRAS-TP53* triple co-mutation, showed associations with survival of patients in the present study. Last, but not least, somatic mutations in MYC, PI3K, RTK-RAS, or immune checkpoint blockade pathway genes are associated with disease stage, but again without prognostic effect. Thus, major oncodrivers did not have a prognostic role in our patient set, but drug sensitivity or resistance-connected genes did.

We also utilized main pathway analysis tools (Reactome, WikiPathways, KEGG) to investigate gene enrichment among all 13 genes with prognostic significance, but failed to identify specific pathways for eventual considerations on their targeting. Thus, together with the lack of information in the current literature about the role of specific genes in CRC progression and therapy resistance beyond the previous GDSC in vitro data, more research is necessary for obtaining exploitable functional evidence.

Intriguingly, we found prognostic associations for several germline variants prioritized using allele frequency and functional predictions. This stringent approach chosen by us has shown that the carriage of variants *NFACS*-rs72753407 or *ERBB4-*rs34621071 was significantly associated with poor RFS and OS. On the other hand, patients with wild-type genotype in *RIF1-*rs2444274 had significantly poorer RFS and OS, although the former did not pass the FDR test. None of these associations was previously reported, and in contrast with somatic data, the prognostic relevance of germline variants for cancer progression and therapy outcome remains rather neglected. In contrast with somatic variants, external validation is currently unavailable for germline data.

Several limitations of the present study cannot be concealed. Firstly, the sample size precludes robust analysis of rare events. Especially, subgroup analyses were underpowered and results that failed to replicate using external dataset need to be interpreted with extreme vigilance. Despite we confirmed the association of *ABCA13, ANK2, COL7A1*, *NAV3*, or *UNC80* with OS in all patients using the external TCGA COAD-READ dataset (Fig. 4), we failed for the rest of the associations, although survival trends remained the same (**Supplementary Fig.****S6**a, d). It is important to stress that our study was based on targeted panel deep sequencing and thus as such contained a much higher number of mutations, especially indels, than the TCGA dataset, which is based mostly on whole exome or genome sequencing. The composition of variants inevitably differed (**Supplementary Fig.****S8**) and consequently could mask essential mutations. In addition, due to some missing data and the heterogeneous nature of the TCGA datasets, it is, however, quite a difficult task to achieve. Therefore, more studies are necessary in this area. On the other hand, the present study has clear benefits in ethnical homogeneity of the patient population, unified therapy regimen, and long-term complete clinical follow-up. Moreover, 5-FU or FOLFOX regimens remain the cornerstone of systemic adjuvant chemotherapy in CRC [27] and thus, composition of our sample set is highly relevant from this point of view.

## Conclusions

We provide a proof-of-principle study using a unique design connecting *in silico* data from several databases containing in vitro functional and ex vivo human tumor datasets that may inspire further research not only on specific genes identified here for CRC but also in a more general fashion aiming exploitation of such already existing resources in future precision oncology concept.

### Electronic supplementary material

Below is the link to the electronic supplementary material.

Supplementary Material 1

Supplementary Material 2

## Data Availability

Data is provided within the manuscript or supplementary information files.
