# Supplementary material for: Targeted panel sequencing of pharmacogenes and oncodrivers in colorectal cancer patients reveals genes with prognostic significance
Source: Hum Genomics. 2024 Jul 19;18:83. doi: 10.1186/s40246-024-00644-2 (PMC11264515; doi:10.1186/s40246-024-00644-2)

Supplementary Figures

Targeted panel sequencing of pharmacogenes and oncodrivers in colorectal cancer patients reveals genes with prognostic significance

Lucie Heczko [1], Václav Liška[1,4], Ondřej Vyčítal [1,4], Ondřej Fiala [1,5], Simona Šůsová[1,2], Viktor Hlaváč [1,2*], Pavel Souček [1,2*]

[1]Biomedical Center, Faculty of Medicine in Pilsen, Charles University, Pilsen, Czech Republic;

[2]Toxicogenomics Unit, National Institute of Public Health, Prague, Czech Republic;

[3]Third Faculty of Medicine, Charles University, Prague, Czech Republic;

[4]Department of Surgery, Faculty of Medicine and University Hospital in Pilsen, Charles University, Pilsen, Czech Republic;

[5]Department of Oncology and Radiotherapeutics, Faculty of Medicine and University Hospital in Pilsen, Charles University, Pilsen, Czech Republic.

Corresponding authors: Pavel Souček, Biomedical Center, Faculty of Medicine in Pilsen, Charles University, alej Svobody Pilsen 306 05, Czech Republic; email: pavel.soucek@lfp.cuni.cz

Viktor Hlaváč, Biomedical Center, Faculty of Medicine in Pilsen, Charles University, alej Svobody Pilsen 306 05, Czech Republic; email: viktor.hlavac@lfp.cuni.cz

Supplementary Figure S1: Kaplan-Meier plots of patient survival stratified by stage and lymph node metastasis status

RFS analysis of stage (a) and lymph node metastasis (b); OS analysis of lymph node metastasis (c).

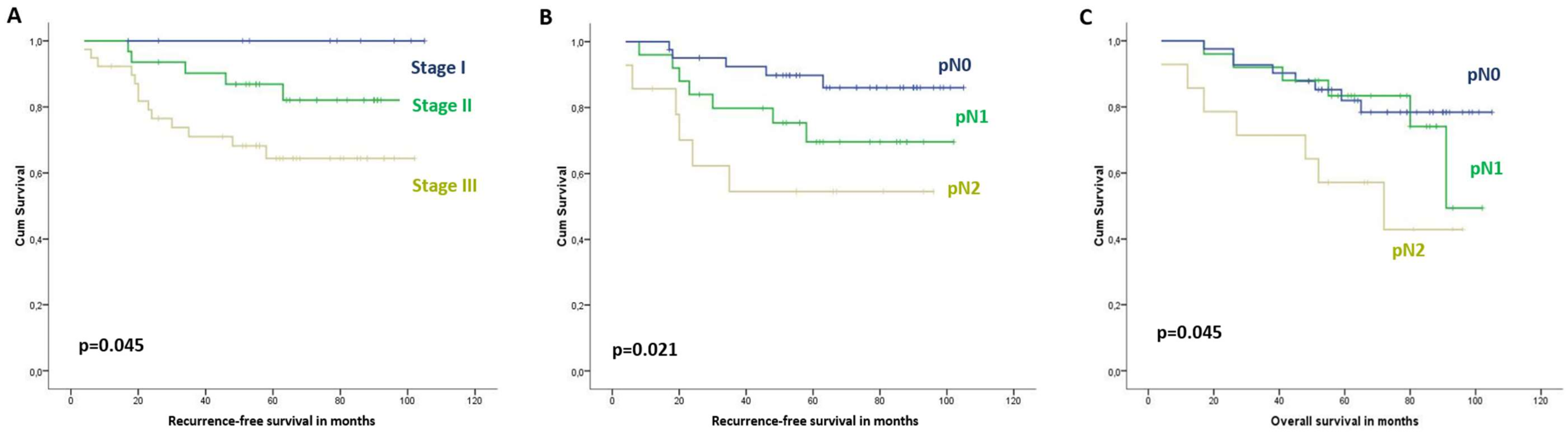

Supplementary Figure S2: Kaplan-Meier plots of patient survival stratified by *APC, TP53,* and *KRAS* somatic mutation status

RFS analysis of *APC* (a), *KRAS-12D* (b); OS analysis of *APC* (c), *KRAS-12D* (d), co-mutated *TP53* with *KRAS-12D* (e), and *TP53* with *KRAS* codon 12 or 13 variants (f). Yellow line represents patients carrying the variant, and the blue line those without for a-d.
Grey line represents patients carrying the *TP53* co-mutated with *KRAS-12D* (e), or *TP53* with *KRAS* codon 12 or 13 variants (f), blue line patients carrying no or single mutation, and yellow line patients with *TP53-KRAS* co-mutated genes with other variants than 12D or those in codons 12 or 13.

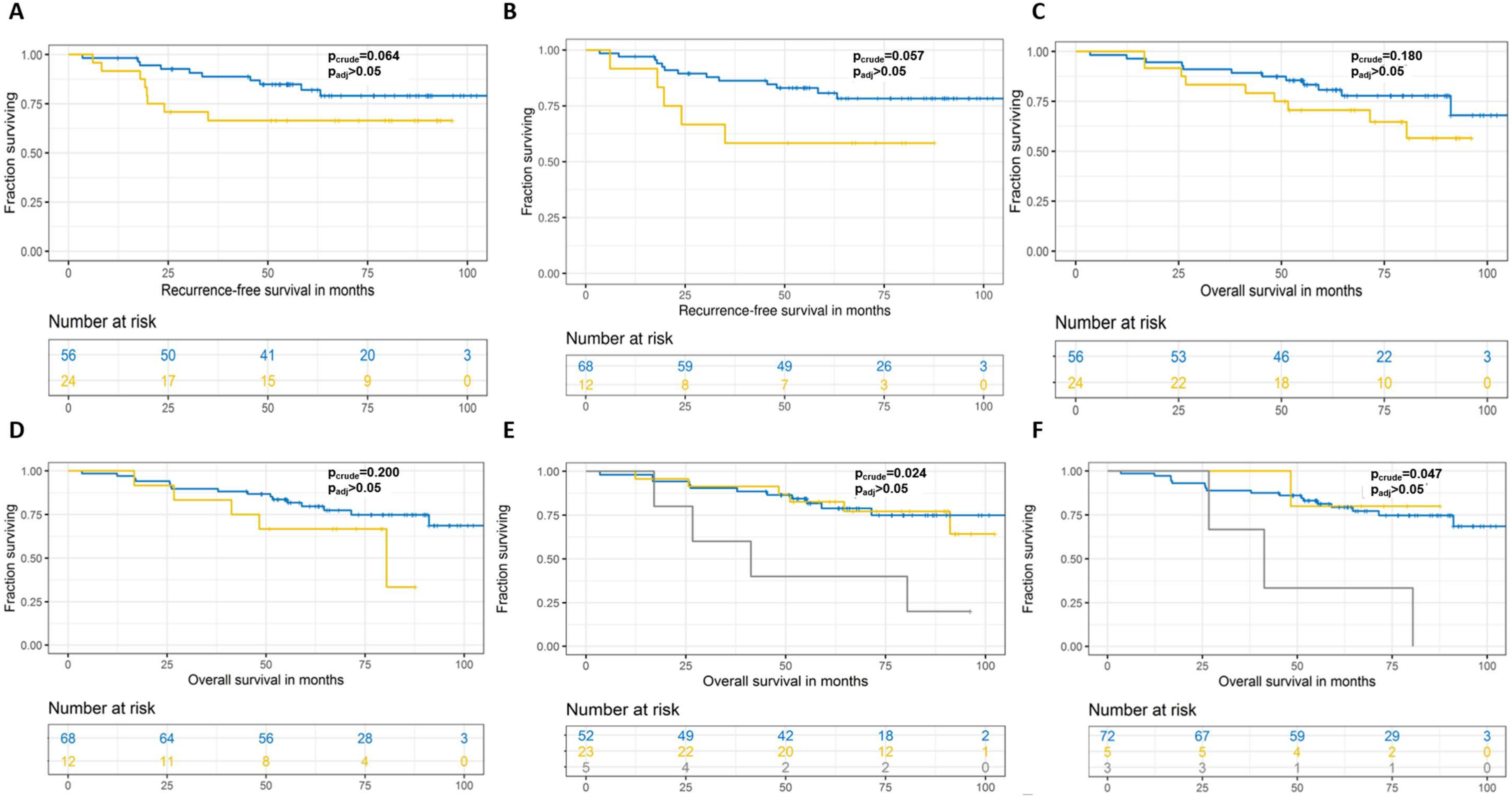

Supplementary Figure S3: Kaplan-Meier plots of patient survival stratified by the carriage of somatic variants in individual genes – all patients

RFS analysis of somatic variants in *ANK2* (a) and *SACS* (b). OS analysis of somatic variants in *ANK2* (c), *ABCA13* (d), *COL7A1* (e), *KMT2D* (f), *NAV3* (g), and *UNC80* (h).
Yellow line represents patients carrying the variant, and the blue line those without.

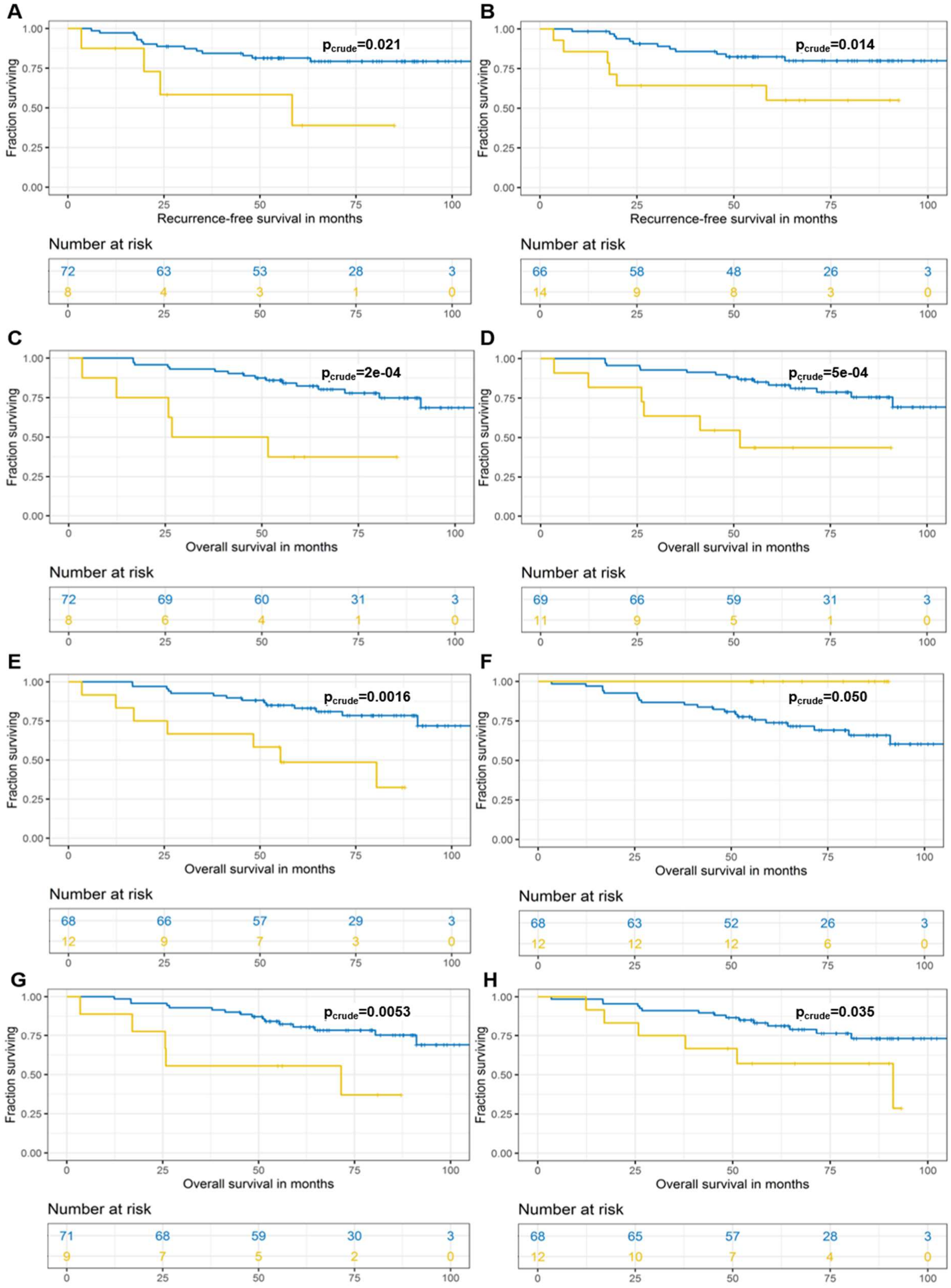

Supplementary Figure S4: Kaplan-Meier plots of patient survival stratified by the carriage of variants in individual genes – untreated patients

OS analysis of somatic variants in *ANK2* (a), *ABCA13* (b), *COL7A1* (c), *FLG* (d), *GLI3* (e), and *UNC80* (f). Yellow line represents patients carrying the variant, and the blue line those without.

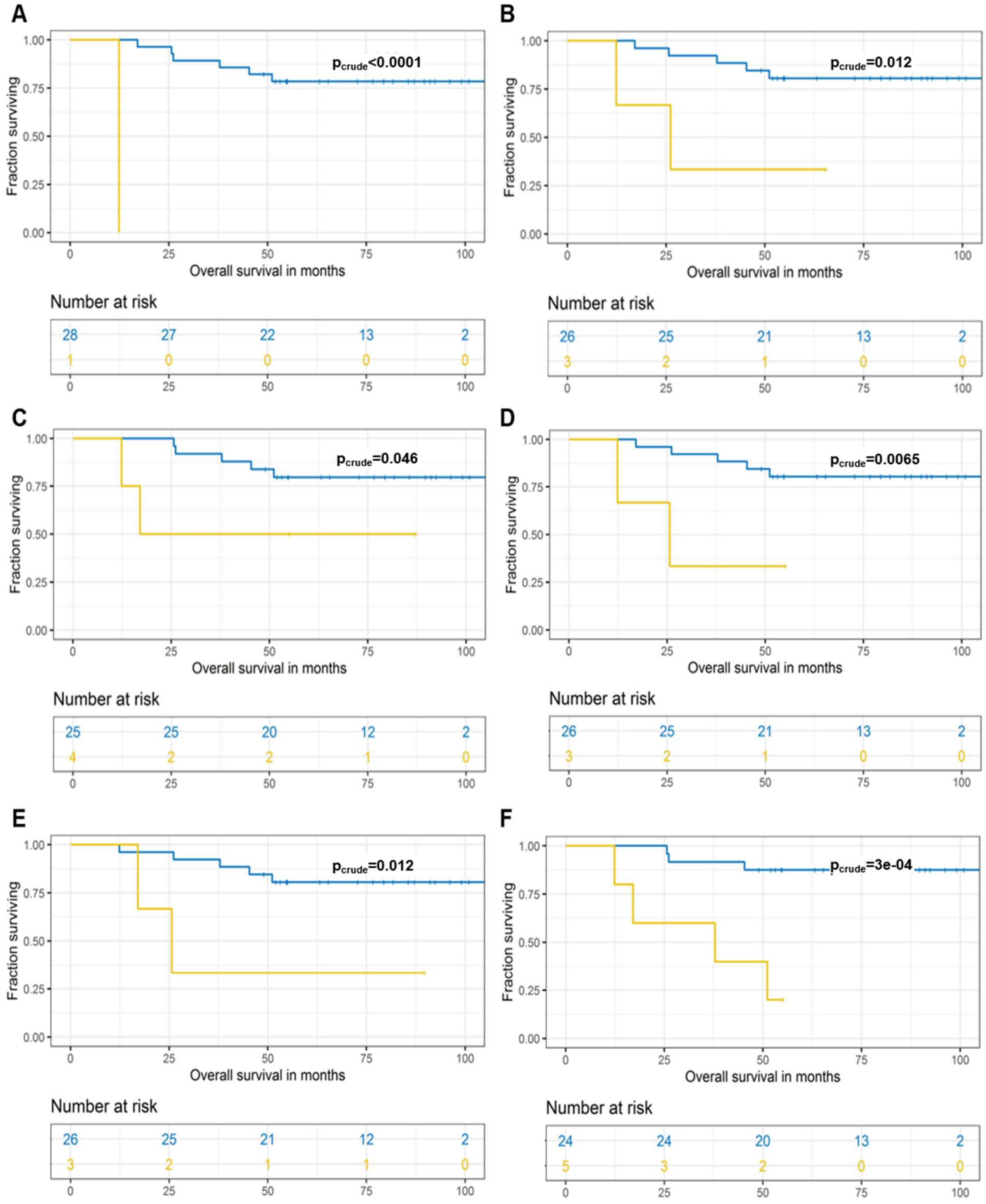

Supplementary Figure S5: Kaplan-Meier plots of patient survival stratified by the carriage of variants in individual genes – adjuvantly treated patients

OS analysis of somatic variants in *ANK2* (a), *ABCA13* (b), *COL6A3* (c), *COL7A1* (d), *LRP1B* (e, *NAV3* (f), *RYR1* (g), *RYR3* (h), *TCCH* (i), and *TENM4* (j).
Yellow line represents patients carrying the variant, and the blue line those without.

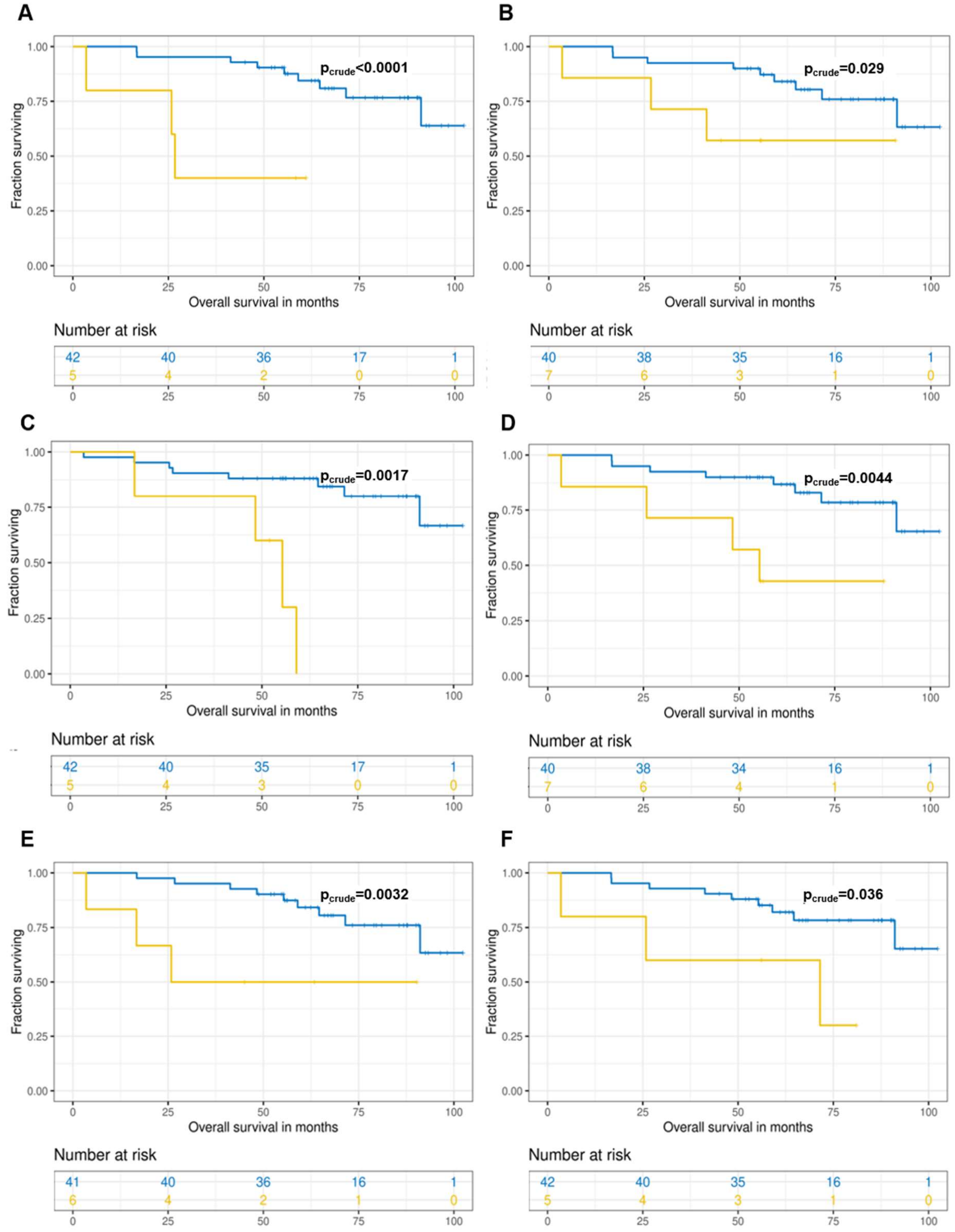

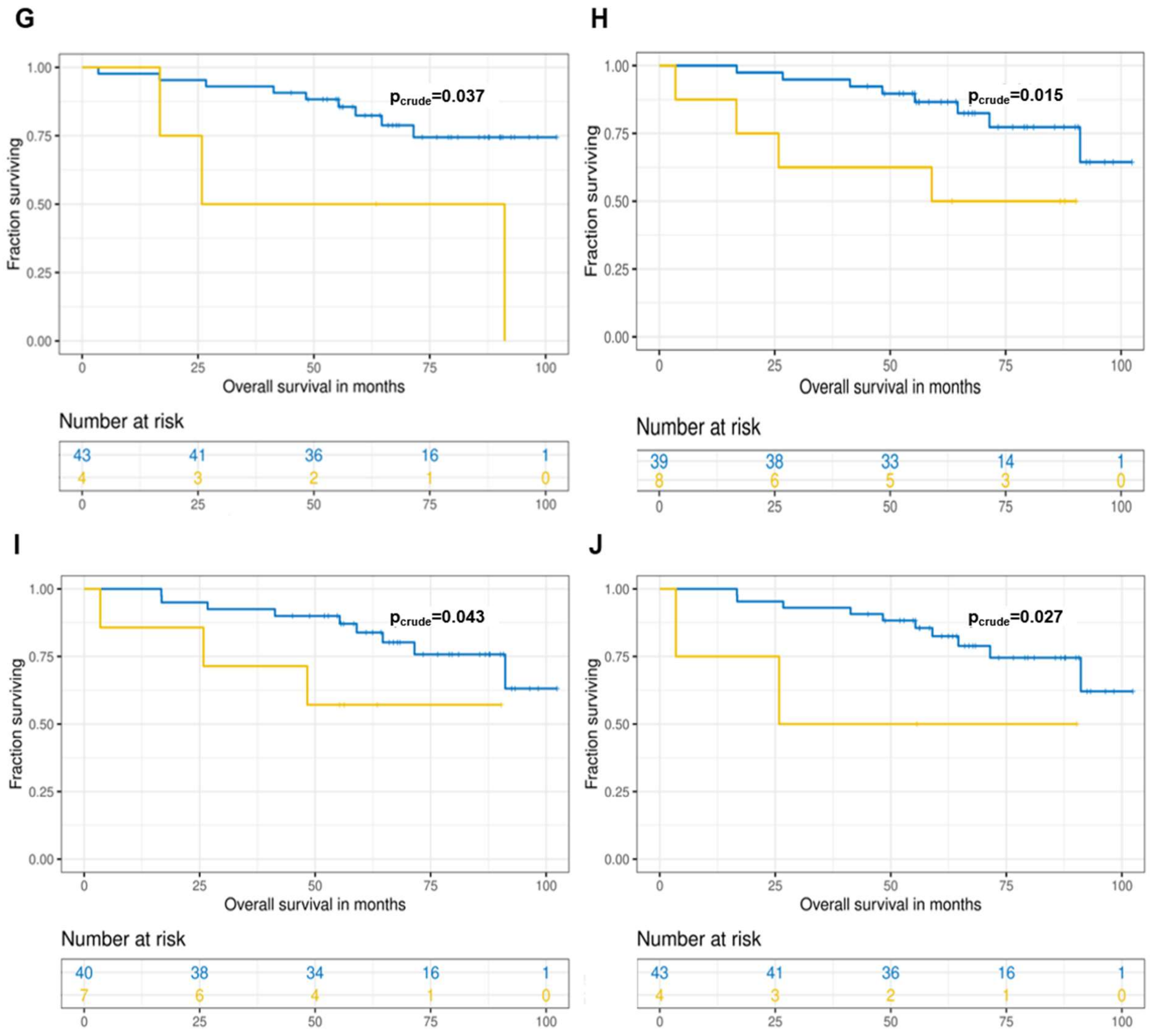

G
H
I
J
Fraction surviving
Overall survival in months
Number at risk
p_{crude}=0.037
p_{crude}=0.015
p_{crude}=0.043
p_{crude}=0.027
43 41 36 16 1
4 3 2 1 0
39 38 33 14 1
8 6 5 3 0
40 38 34 16 1
7 6 4 1 0
43 41 36 16 1
4 3 2 1 0

Supplementary Figure S6: Kaplan-Meier plots of patient survival stratified by the carriage of variants in individual genes – TCGA COAD-READ dataset (n=533)

OS analysis of somatic variants in *ANK2-ABCA13-COL7A1* in all (a), untreated (b), and adjuvantly treated (c); *ANK2-ABCA13-COL7A1-NAV3-UNC80* in all (d), untreated (e), and adjuvantly treated (f); *FLG-GLI3-UNC80* in all (g), untreated (h), and adjuvantly treated (i); *COL6A3-LRP1B-NAV3-RYR1-RYR3-TCHH-TENM4* in all (j), untreated (k), and adjuvantly treated (l) patients.
Blue line represents patients without mutations, the yellow line patients carrying mutations in single gene, and the grey line those with mutations in more than one gene (where applicable).

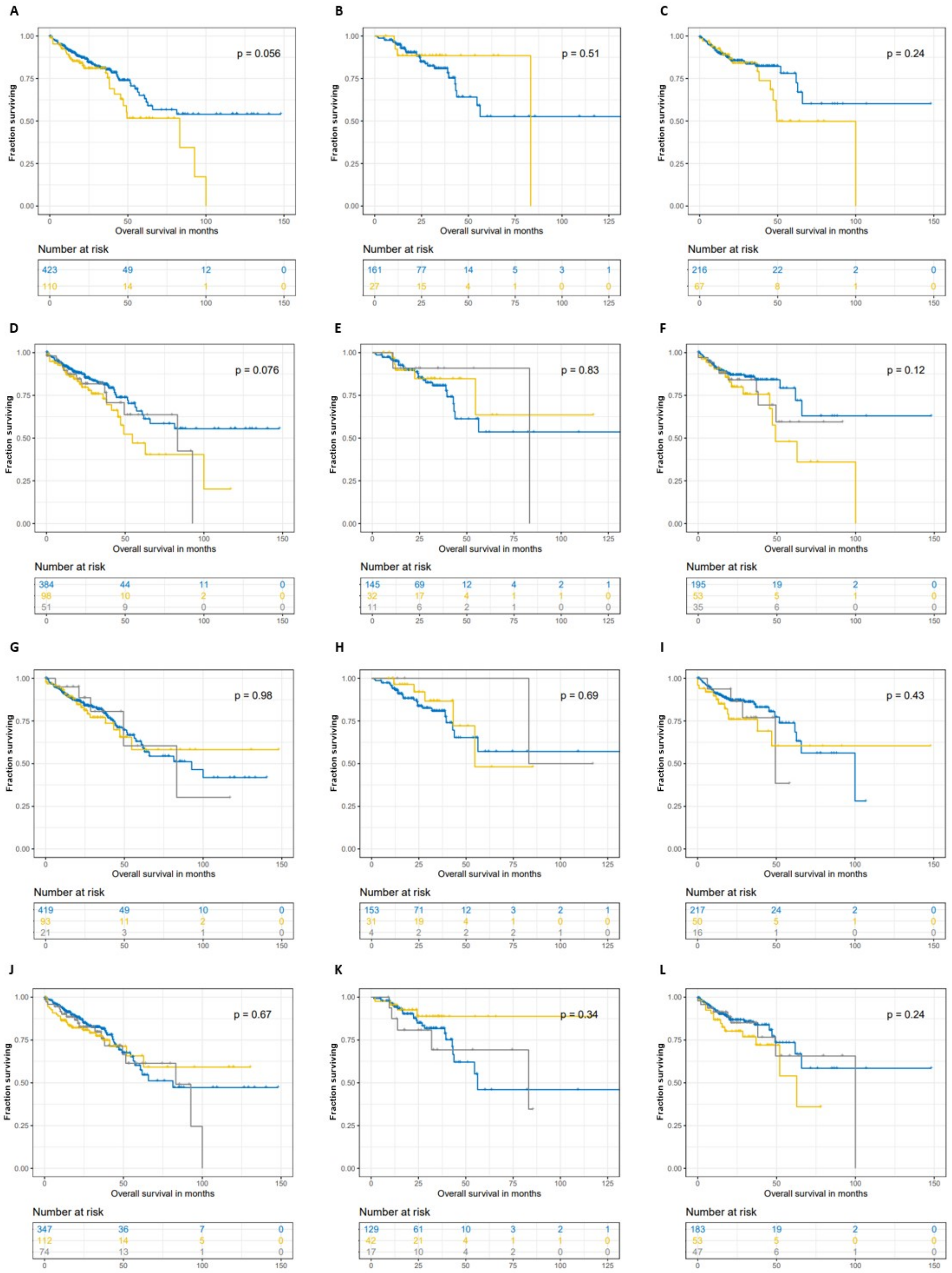

Supplementary Figure S7: Manhattan plot of germline variability in patients stratified by recurrence-free survival at 3-year time point

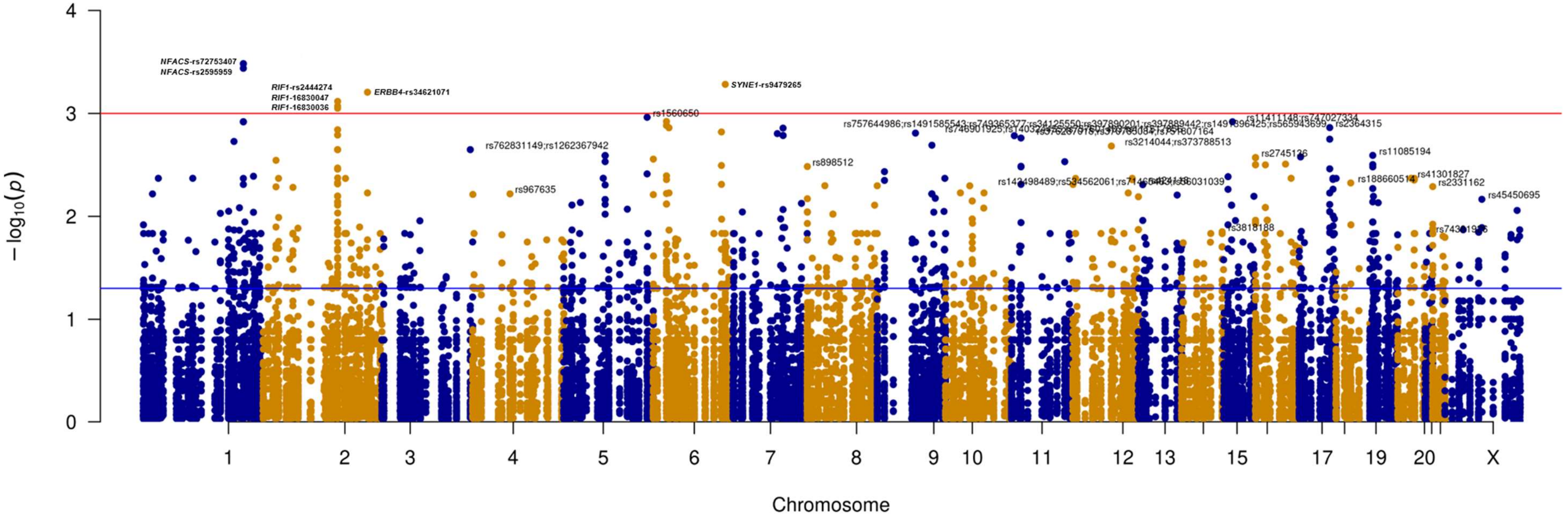

Variants rs2595959 and rs72753407 in *NFACS*, rs2444274, rs16830036 and rs16830047 in *RIF1*, rs34621071 in *ERBB4*, and rs9479265 in SYNE1 were tested for associations with survival (RFS and OS) using Kaplan-Meier method – see Fig. 5 and S8.

Supplementary Figure S8: The comparison of somatic mutations in candidate genes associated with survival between panel sequencing (a) and TCGA COAD-READ (b)

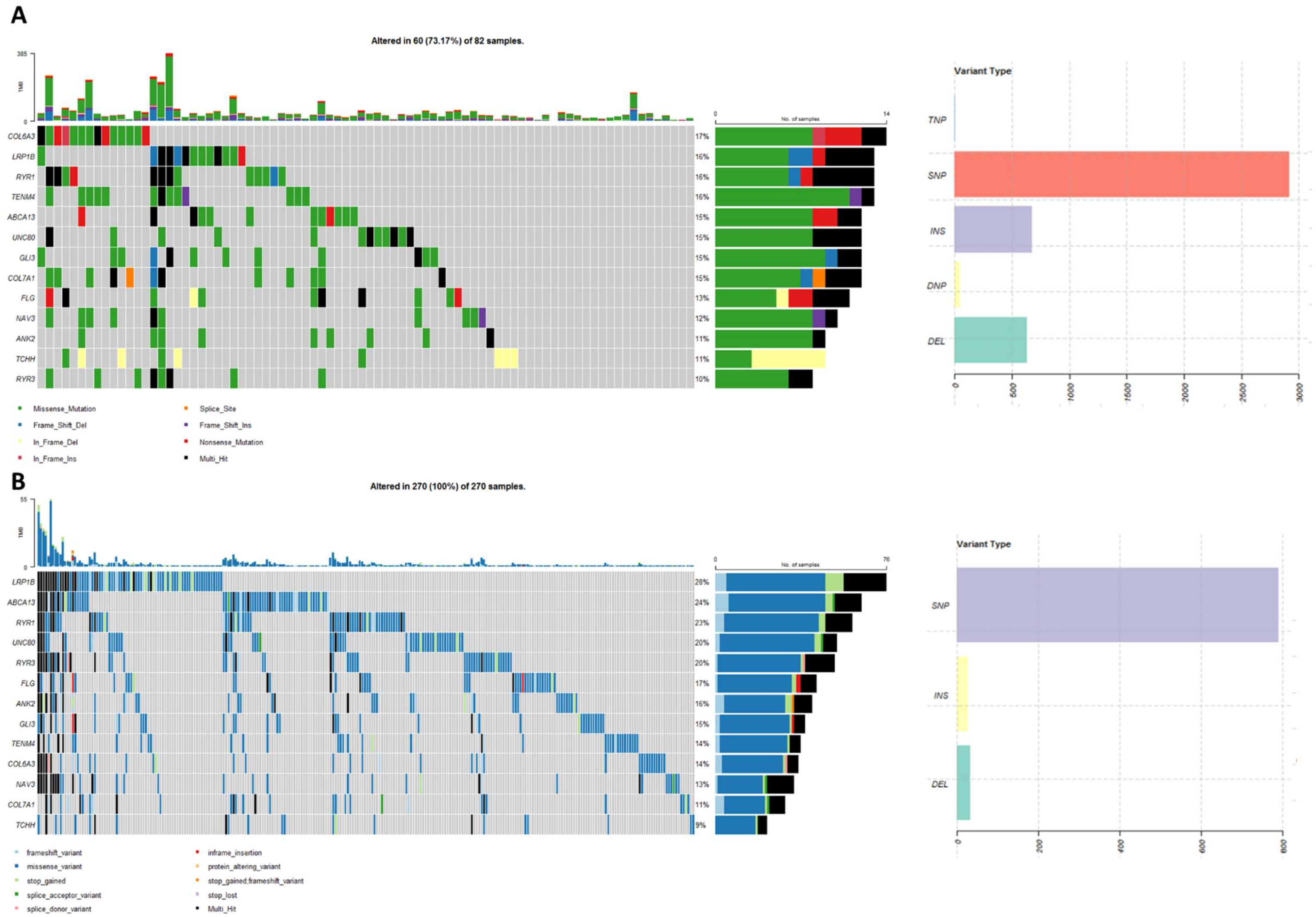

Supplement: Supplementary file 1 — Supplementary Material 1 [file 40246_2024_644_MOESM1_ESM.pdf]
