# Supplementary material for: Targeted panel sequencing of pharmacogenes and oncodrivers in colorectal cancer patients reveals genes with prognostic significance
Source: Hum Genomics. 2024 Jul 19;18:83. doi: 10.1186/s40246-024-00644-2 (PMC11264515; doi:10.1186/s40246-024-00644-2)
Supplement: Supplementary file 2 — Supplementary Material 2 [file 40246_2024_644_MOESM2_ESM.pdf]

Additional File 2 - Supplementary Tables

Targeted panel sequencing of pharmacogenes and oncodrivers in colorectal cancer patients reveals genes with prognostic significance

Lucie Heczko [1], Václav Liška[1,4], Ondřej Vyčítal [1,4], Ondřej Fiala [1,5], Simona Šůsová[1,2], Viktor Hlaváč [1,2*], Pavel Souček [1,2*]

[1]Biomedical Center, Faculty of Medicine in Pilsen, Charles University, Pilsen, Czech Republic;
[2]Toxicogenomics Unit, National Institute of Public Health, Prague, Czech Republic;
[3]Third Faculty of Medicine, Charles University, Prague, Czech Republic;
[4]Department of Surgery, Faculty of Medicine and University Hospital in Pilsen, Charles University, Pilsen, Czech Republic;

Corresponding authors: Pavel Souček, Biomedical Center, Faculty of Medicine in Pilsen, Charles University, alej Svobody Pilsen 306 05, Czech Republic; email: pavel.soucek@lfp.cuni.cz
Viktor Hlaváč, Biomedical Center, Faculty of Medicine in Pilsen, Charles University, alej Svobody Pilsen 306 05, Czech Republic; email: viktor.hlavac@lfp.cuni.cz

Supplementary Table S1: List of genes in the panel

Gene	Match type	Approved symbol	Approved name	HGNC ID	Location
A2ML1	Approved symbol	A2ML1	alpha-2-macroglobulin like 1	HGNC:23336	12p13.31
ABCA1	Approved symbol	ABCA1	ATP binding cassette subfamily A member 1	HGNC:29	9q31.1
ABCA12	Approved symbol	ABCA12	ATP binding cassette subfamily A member 12	HGNC:14637	2q35
ABCA13	Approved symbol	ABCA13	ATP binding cassette subfamily A member 13	HGNC:14638	7p12.3
ABCA2	Approved symbol	ABCA2	ATP binding cassette subfamily A member 2	HGNC:32	9q34.3
ABCA3	Approved symbol	ABCA3	ATP binding cassette subfamily A member 3	HGNC:33	16p13.3
ABCA4	Approved symbol	ABCA4	ATP binding cassette subfamily A member 4	HGNC:34	1p22.1
ABCA6	Approved symbol	ABCA6	ATP binding cassette subfamily A member 6	HGNC:36	17q24.2-q24.3
ABCA7	Approved symbol	ABCA7	ATP binding cassette subfamily A member 7	HGNC:37	19p13.3
ABCC1	Approved symbol	ABCC1	ATP binding cassette subfamily C member 1 (ABCC1 blood group)	HGNC:51	16p13.11
ABCC5	Approved symbol	ABCC5	ATP binding cassette subfamily C member 5	HGNC:56	3q27.1
ABCC8	Approved symbol	ABCC8	ATP binding cassette subfamily C member 8	HGNC:59	11p15.1
ACACB	Approved symbol	ACACB	acetyl-CoA carboxylase beta	HGNC:85	12q24.11
ACAN	Approved symbol	ACAN	aggrecan	HGNC:319	15q26.1
ACOD1	Approved symbol	ACOD1	aconitate decarboxylase 1	HGNC:33904	13q22.3
ADAMTS12	Approved symbol	ADAMTS12	ADAM metallopeptidase with thrombospondin type 1 motif 12	HGNC:14605	5p13.3-p13.2
ADAMTS16	Approved symbol	ADAMTS16	ADAM metallopeptidase with thrombospondin type 1 motif 16	HGNC:17108	5p15.32
ADAMTS18	Approved symbol	ADAMTS18	ADAM metallopeptidase with thrombospondin type 1 motif 18	HGNC:17110	16q23.1
ADAMTS20	Approved symbol	ADAMTS20	ADAM metallopeptidase with thrombospondin type 1 motif 20	HGNC:17178	12q12
ADAMTS9	Approved symbol	ADAMTS9	ADAM metallopeptidase with thrombospondin type 1 motif 9	HGNC:13202	3p14.1
ADAMTSL3	Approved symbol	ADAMTSL3	ADAMTS like 3	HGNC:14633	15q25.2
ADCY8	Approved symbol	ADCY8	adenylate cyclase 8	HGNC:239	8q24.22
ADGRB1	Approved symbol	ADGRB1	adhesion G protein-coupled receptor B1	HGNC:943	8q24.3
ADGRB3	Approved symbol	ADGRB3	adhesion G protein-coupled receptor B3	HGNC:945	6q12-q13
ADGRG4	Approved symbol	ADGRG4	adhesion G protein-coupled receptor G4	HGNC:18992	Xq26.3
ADGRL2	Approved symbol	ADGRL2	adhesion G protein-coupled receptor L2	HGNC:18582	1p31.1
ADGRL3	Approved symbol	ADGRL3	adhesion G protein-coupled receptor L3	HGNC:20974	4q13.1
ADGRV1	Approved symbol	ADGRV1	adhesion G protein-coupled receptor V1	HGNC:17416	5q14.3
AFDN	Approved symbol	AFDN	afadin, adherens junction formation factor	HGNC:7137	6q27
AFF2	Approved symbol	AFF2	ALF transcription elongation factor 2	HGNC:3776	Xq28
AHNAK	Approved symbol	AHNAK	AHNAK nucleoprotein	HGNC:347	11q12.3
AHNAK2	Approved symbol	AHNAK2	AHNAK nucleoprotein 2	HGNC:20125	14q32.33
AKAP12	Approved symbol	AKAP12	A-kinase anchoring protein 12	HGNC:370	6q25.1
AKAP13	Approved symbol	AKAP13	A-kinase anchoring protein 13	HGNC:371	15q25.3
AKAP9	Approved symbol	AKAP9	A-kinase anchoring protein 9	HGNC:379	7q21.2
ALK	Approved symbol	ALK	ALK receptor tyrosine kinase	HGNC:427	2p23.2-p23.1

ANK1	Approved symbol	ANK1	ankyrin 1	HGNC:492	8p11.21
ANK2	Approved symbol	ANK2	ankyrin 2	HGNC:493	4q25-q26
ANK3	Approved symbol	ANK3	ankyrin 3	HGNC:494	10q21.2
ANKRD11	Approved symbol	ANKRD11	ankyrin repeat domain containing 11	HGNC:21316	16q24.3
ANKRD30A	Approved symbol	ANKRD30A	ankyrin repeat domain 30A	HGNC:17234	10p11.21
APBA1	Approved symbol	APBA1	amyloid beta precursor protein binding family A member 1	HGNC:578	9q21.12
APC	Approved symbol	APC	APC regulator of WNT signaling pathway	HGNC:583	5q22.2
APOB	Approved symbol	APOB	apolipoprotein B	HGNC:603	2p24.1
ARHGEF17	Approved symbol	ARHGEF17	Rho guanine nucleotide exchange factor 17	HGNC:21726	11q13.4
ARID1A	Approved symbol	ARID1A	AT-rich interaction domain 1A	HGNC:11110	1p36.11
ARID2	Approved symbol	ARID2	AT-rich interaction domain 2	HGNC:18037	12q12
ASPM	Approved symbol	ASPM	assembly factor for spindle microtubules	HGNC:19048	1q31.3
ASTN2	Approved symbol	ASTN2	astrotactin 2	HGNC:17021	9q33.1
ASXL1	Approved symbol	ASXL1	ASXL transcriptional regulator 1	HGNC:18318	20q11.21
ASXL3	Approved symbol	ASXL3	ASXL transcriptional regulator 3	HGNC:29357	18q12.1
ATM	Approved symbol	ATM	ATM serine/threonine kinase	HGNC:795	11q22.3
ATP10A	Approved symbol	ATP10A	ATPase phospholipid transporting 10A (putative)	HGNC:13542	15q12
ATP2B3	Approved symbol	ATP2B3	ATPase plasma membrane Ca2+ transporting 3	HGNC:816	Xq28
ATP8A2	Approved symbol	ATP8A2	ATPase phospholipid transporting 8A2	HGNC:13533	13q12.13
ATRX	Approved symbol	ATRX	ATRX chromatin remodeler	HGNC:886	Xq21.1
B4GALNT2	Approved symbol	B4GALNT2	beta-1,4-N-acetyl-galactosaminyltransferase 2 (SID blood group)	HGNC:24136	17q21.32
BCL9	Approved symbol	BCL9	BCL9 transcription coactivator	HGNC:1008	1q21.2
BCOR	Approved symbol	BCOR	BCL6 corepressor	HGNC:20893	Xp11.4
BCORL1	Approved symbol	BCORL1	BCL6 corepressor like 1	HGNC:25657	Xq26.1
BIRC6	Approved symbol	BIRC6	baculoviral IAP repeat containing 6	HGNC:13516	2p22.3
KIAA1109	Previous symbol	BLTP1	bridge-like lipid transfer protein family member 1	HGNC:26953	4q27
BMPR2	Approved symbol	BMPR2	bone morphogenetic protein receptor type 2	HGNC:1078	2q33.1-q33.2
BOD1L1	Approved symbol	BOD1L1	biorientation of chromosomes in cell division 1 like 1	HGNC:31792	4p15.33
BRAF	Approved symbol	BRAF	B-Raf proto-oncogene, serine/threonine kinase	HGNC:1097	7q34
BRCA2	Approved symbol	BRCA2	BRCA2 DNA repair associated	HGNC:1101	13q13.1
BRD4	Approved symbol	BRD4	bromodomain containing 4	HGNC:13575	19p13.12
BRINP3	Approved symbol	BRINP3	BMP/retinoic acid inducible neural specific 3	HGNC:22393	1q31.1
BSN	Approved symbol	BSN	bassoon presynaptic cytomatrix protein	HGNC:1117	3p21.31
C1D	Approved symbol	C1D	C1D nuclear receptor corepressor	HGNC:29911	2p14
C3	Approved symbol	C3	complement C3	HGNC:1318	19p13.3
CACNA1A	Approved symbol	CACNA1A	calcium voltage-gated channel subunit alpha1 A	HGNC:1388	19p13.13
CACNA1B	Approved symbol	CACNA1B	calcium voltage-gated channel subunit alpha1 B	HGNC:1389	9q34.3
CACNA1E	Approved symbol	CACNA1E	calcium voltage-gated channel subunit alpha1 E	HGNC:1392	1q25.3
CACNA1G	Approved symbol	CACNA1G	calcium voltage-gated channel subunit alpha1 G	HGNC:1394	17q21.33

CACNA1H	Approved symbol	CACNA1H	calcium voltage-gated channel subunit alpha1 H	HGNC:1395	16p13.3
CAD	Approved symbol	CAD	carbamoyl-phosphate synthetase 2, aspartate transcarbamylase, and dihydroorotase	HGNC:1424	2p23.3
CAMSAP1	Approved symbol	CAMSAP1	calmodulin regulated spectrin associated protein 1	HGNC:19946	9q34.3
CARD11	Approved symbol	CARD11	caspase recruitment domain family member 11	HGNC:16393	7p22.2
CASZ1	Approved symbol	CASZ1	castor zinc finger 1	HGNC:26002	1p36.22
CD36	Approved symbol	CD36	CD36 molecule (CD36 blood group)	HGNC:1663	7q21.11
CDC42BPA	Approved symbol	CDC42BPA	CDC42 binding protein kinase alpha	HGNC:1737	1q42.13
CDH10	Approved symbol	CDH10	cadherin 10	HGNC:1749	5p14.2-p14.1
CDH18	Approved symbol	CDH18	cadherin 18	HGNC:1757	5p14.3
CDH2	Approved symbol	CDH2	cadherin 2	HGNC:1759	18q12.1
CDH23	Approved symbol	CDH23	cadherin related 23	HGNC:13733	10q22.1
CDH8	Approved symbol	CDH8	cadherin 8	HGNC:1767	16q21
CDH9	Approved symbol	CDH9	cadherin 9	HGNC:1768	5p14.1
CELSR1	Approved symbol	CELSR1	cadherin EGF LAG seven-pass G-type receptor 1	HGNC:1850	22q13.31
CELSR2	Approved symbol	CELSR2	cadherin EGF LAG seven-pass G-type receptor 2	HGNC:3231	1p13.3
CELSR3	Approved symbol	CELSR3	cadherin EGF LAG seven-pass G-type receptor 3	HGNC:3230	3p21.31
CEP250	Approved symbol	CEP250	centrosomal protein 250	HGNC:1859	20q11.22
CEP350	Approved symbol	CEP350	centrosomal protein 350	HGNC:24238	1q25.2
CMYA5	Approved symbol	CMYA5	cardiomyopathy associated 5	HGNC:14305	5q14.1
CNKSR3	Approved symbol	CNKSR3	CNKSR family member 3	HGNC:23034	6q25.2
CNOT1	Approved symbol	CNOT1	CCR4-NOT transcription complex subunit 1	HGNC:7877	16q21
CNTN6	Approved symbol	CNTN6	contactin 6	HGNC:2176	3p26.3
CNTNAP4	Approved symbol	CNTNAP4	contactin associated protein family member 4	HGNC:18747	16q23.1
CNTNAP5	Approved symbol	CNTNAP5	contactin associated protein family member 5	HGNC:18748	2q14.3
CNTRL	Approved symbol	CNTRL	centriolin	HGNC:1858	9q33.2
COL11A1	Approved symbol	COL11A1	collagen type XI alpha 1 chain	HGNC:2186	1p21.1
COL12A1	Approved symbol	COL12A1	collagen type XII alpha 1 chain	HGNC:2188	6q13-q14.1
COL22A1	Approved symbol	COL22A1	collagen type XXII alpha 1 chain	HGNC:22989	8q24.23-q24.3
COL27A1	Approved symbol	COL27A1	collagen type XXVII alpha 1 chain	HGNC:22986	9q32
COL4A1	Approved symbol	COL4A1	collagen type IV alpha 1 chain	HGNC:2202	13q34
COL4A2	Approved symbol	COL4A2	collagen type IV alpha 2 chain	HGNC:2203	13q34
COL5A1	Approved symbol	COL5A1	collagen type V alpha 1 chain	HGNC:2209	9q34.3
COL5A2	Approved symbol	COL5A2	collagen type V alpha 2 chain	HGNC:2210	2q32.2
COL6A3	Approved symbol	COL6A3	collagen type VI alpha 3 chain	HGNC:2213	2q37.3
COL6A6	Approved symbol	COL6A6	collagen type VI alpha 6 chain	HGNC:27023	3q22.1
COL7A1	Approved symbol	COL7A1	collagen type VII alpha 1 chain	HGNC:2214	3p21.31
CPAMD8	Approved symbol	CPAMD8	C3 and PZP like alpha-2-macroglobulin domain containing 8	HGNC:23228	19p13.11
C5orf42	Previous symbol	CPLANE1	ciliogenesis and planar polarity effector complex subunit 1	HGNC:25801	5p13.2
CR1	Approved symbol	CR1	complement C3b/C4b receptor 1 (Knops blood group)	HGNC:2334	1q32.2

CREBBP	Approved symbol	CREBBP	CREB binding protein	HGNC:2348	16p13.3
CRMP1	Approved symbol	CRMP1	collapsin response mediator protein 1	HGNC:2365	4p16.2
CSMD1	Approved symbol	CSMD1	CUB and Sushi multiple domains 1	HGNC:14026	8p23.2
CSMD2	Approved symbol	CSMD2	CUB and Sushi multiple domains 2	HGNC:19290	1p35.1
CSMD3	Approved symbol	CSMD3	CUB and Sushi multiple domains 3	HGNC:19291	8q23.3
CSPG4	Approved symbol	CSPG4	chondroitin sulfate proteoglycan 4	HGNC:2466	15q24.2
CTNNB1	Approved symbol	CTNNB1	catenin beta 1	HGNC:2514	3p22.1
CUBN	Approved symbol	CUBN	cubilin	HGNC:2548	10p13
CUL9	Approved symbol	CUL9	cullin 9	HGNC:15982	6p21.1
DAPK1	Approved symbol	DAPK1	death associated protein kinase 1	HGNC:2674	9q21.33
DCC	Approved symbol	DCC	DCC netrin 1 receptor	HGNC:2701	18q21.2
DCLK1	Approved symbol	DCLK1	doublecortin like kinase 1	HGNC:2700	13q13.3
DFFB	Approved symbol	DFFB	DNA fragmentation factor subunit beta	HGNC:2773	1p36.32
DCHS1	Approved symbol	DCHS1	dachsous cadherin-related 1	HGNC:13681	11p15.4
DCHS2	Approved symbol	DCHS2	dachsous cadherin-related 2	HGNC:23111	4q31.3
DIDO1	Approved symbol	DIDO1	death inducer-obliterator 1	HGNC:2680	20q13.33
DIP2C	Approved symbol	DIP2C	disco interacting protein 2 homolog C	HGNC:29150	10p15.3
DLC1	Approved symbol	DLC1	DLC1 Rho GTPase activating protein	HGNC:2897	8p22
DLEC1	Approved symbol	DLEC1	DLEC1 cilia and flagella associated protein	HGNC:2899	3p22.2
DLGAP2	Approved symbol	DLGAP2	DLG associated protein 2	HGNC:2906	8p23.3
DMBT1	Approved symbol	DMBT1	deleted in malignant brain tumors 1	HGNC:2926	10q26.13
DMD	Approved symbol	DMD	dystrophin	HGNC:2928	Xp21.2-p21.1
DMXL2	Approved symbol	DMXL2	Dmx like 2	HGNC:2938	15q21.2
DNAH1	Approved symbol	DNAH1	dynein axonemal heavy chain 1	HGNC:2940	3p21.1
DNAH10	Approved symbol	DNAH10	dynein axonemal heavy chain 10	HGNC:2941	12q24.31
DNAH11	Approved symbol	DNAH11	dynein axonemal heavy chain 11	HGNC:2942	7p15.3
DNAH14	Approved symbol	DNAH14	dynein axonemal heavy chain 14	HGNC:2945	1q42.12
DNAH17	Approved symbol	DNAH17	dynein axonemal heavy chain 17	HGNC:2946	17q25.3
DNAH2	Approved symbol	DNAH2	dynein axonemal heavy chain 2	HGNC:2948	17p13.1
DNAH3	Approved symbol	DNAH3	dynein axonemal heavy chain 3	HGNC:2949	16p12.3
DNAH5	Approved symbol	DNAH5	dynein axonemal heavy chain 5	HGNC:2950	5p15.2
DNAH7	Approved symbol	DNAH7	dynein axonemal heavy chain 7	HGNC:18661	2q32.3
DNAH8	Approved symbol	DNAH8	dynein axonemal heavy chain 8	HGNC:2952	6p21.2
DNAH9	Approved symbol	DNAH9	dynein axonemal heavy chain 9	HGNC:2953	17p12
DOCK2	Approved symbol	DOCK2	dedicator of cytokinesis 2	HGNC:2988	5q35.1
DOCK3	Approved symbol	DOCK3	dedicator of cytokinesis 3	HGNC:2989	3p21.2
DOCK4	Approved symbol	DOCK4	dedicator of cytokinesis 4	HGNC:19192	7q31.1
DOCK5	Approved symbol	DOCK5	dedicator of cytokinesis 5	HGNC:23476	8p21.2
DOCK8	Approved symbol	DOCK8	dedicator of cytokinesis 8	HGNC:19191	9p24.3

DPYD	Approved symbol	DPYD	dihydropyrimidine dehydrogenase	HGNC:3012	1p21.3
DSCAM	Approved symbol	DSCAM	DS cell adhesion molecule	HGNC:3039	21q22.2
DSCAML1	Approved symbol	DSCAML1	DS cell adhesion molecule like 1	HGNC:14656	11q23.3
DSEL	Approved symbol	DSEL	dermatan sulfate epimerase like	HGNC:18144	18q22.1
DSP	Approved symbol	DSP	desmoplakin	HGNC:3052	6p24.3
DST	Approved symbol	DST	dystonin	HGNC:1090	6p12.1
DYNC1H1	Approved symbol	DYNC1H1	dynein cytoplasmic 1 heavy chain 1	HGNC:2961	14q32.31
DYNLL1	Approved symbol	DYNLL1	dynein light chain LC8-type 1	HGNC:15476	12q24.31
DYSF	Approved symbol	DYSF	dysferlin	HGNC:3097	2p13.2
EGFR	Approved symbol	EGFR	epidermal growth factor receptor	HGNC:3236	7p11.2
EP300	Approved symbol	EP300	E1A binding protein p300	HGNC:3373	22q13.2
EP400	Approved symbol	EP400	E1A binding protein p400	HGNC:11958	12q24.33
EPHA3	Approved symbol	EPHA3	EPH receptor A3	HGNC:3387	3p11.1
EPHB1	Approved symbol	EPHB1	EPH receptor B1	HGNC:3392	3q22.2
ERBB4	Approved symbol	ERBB4	erb-b2 receptor tyrosine kinase 4	HGNC:3432	2q34
ERICH3	Approved symbol	ERICH3	glutamate rich 3	HGNC:25346	1p31.1
ESRP1	Approved symbol	ESRP1	epithelial splicing regulatory protein 1	HGNC:25966	8q22.1
EVPL	Approved symbol	EVPL	envoplakin	HGNC:3503	17q25.1
EYS	Approved symbol	EYS	eyes shut homolog	HGNC:21555	6q12
F8	Approved symbol	F8	coagulation factor VIII	HGNC:3546	Xq28
FAM135B	Approved symbol	FAM135B	family with sequence similarity 135 member B	HGNC:28029	8q24.23
FANCM	Approved symbol	FANCM	FA complementation group M	HGNC:23168	14q21.2
FASN	Approved symbol	FASN	fatty acid synthase	HGNC:3594	17q25.3
FAT1	Approved symbol	FAT1	FAT atypical cadherin 1	HGNC:3595	4q35.2
FAT2	Approved symbol	FAT2	FAT atypical cadherin 2	HGNC:3596	5q33.1
FAT3	Approved symbol	FAT3	FAT atypical cadherin 3	HGNC:23112	11q14.3
FAT4	Approved symbol	FAT4	FAT atypical cadherin 4	HGNC:23109	4q28.1
FBN1	Approved symbol	FBN1	fibrillin 1	HGNC:3603	15q21.1
FBN2	Approved symbol	FBN2	fibrillin 2	HGNC:3604	5q23.3
FBN3	Approved symbol	FBN3	fibrillin 3	HGNC:18794	19p13.2
FBXW7	Approved symbol	FBXW7	F-box and WD repeat domain containing 7	HGNC:16712	4q31.3
FER1L6	Approved symbol	FER1L6	fer-1 like family member 6	HGNC:28065	8q24.13
FGFR1	Approved symbol	FGFR1	fibroblast growth factor receptor 1	HGNC:3688	8p11.23
FHOD3	Approved symbol	FHOD3	formin homology 2 domain containing 3	HGNC:26178	18q12.2
FLG	Approved symbol	FLG	filaggrin	HGNC:3748	1q21.3
FLNB	Approved symbol	FLNB	filamin B	HGNC:3755	3p14.3
FLNC	Approved symbol	FLNC	filamin C	HGNC:3756	7q32.1
FLT4	Approved symbol	FLT4	fms related receptor tyrosine kinase 4	HGNC:3767	5q35.3
FMN2	Approved symbol	FMN2	formin 2	HGNC:14074	1q43

FN1	Approved symbol	FN1	fibronectin 1	HGNC:3778	2q35
FNDC1	Approved symbol	FNDC1	fibronectin type III domain containing 1	HGNC:21184	6q25.3
FRAS1	Approved symbol	FRAS1	Fraser extracellular matrix complex subunit 1	HGNC:19185	4q21.21
FREM1	Approved symbol	FREM1	FRAS1 related extracellular matrix 1	HGNC:23399	9p22.3
FREM2	Approved symbol	FREM2	FRAS1 related extracellular matrix 2	HGNC:25396	13q13.3
FRMD4A	Approved symbol	FRMD4A	FERM domain containing 4A	HGNC:25491	10p13
FRY	Approved symbol	FRY	FRY microtubule binding protein	HGNC:20367	13q13.1
GCN1	Approved symbol	GCN1	GCN1 activator of EIF2AK4	HGNC:4199	12q24.23
WRB	Previous symbol	GET1	guided entry of tail-anchored proteins factor 1	HGNC:12790	21q22.2
GLI3	Approved symbol	GLI3	GLI family zinc finger 3	HGNC:4319	7p14.1
GOLGB1	Approved symbol	GOLGB1	golgin B1	HGNC:4429	3q13.33
GREB1	Approved symbol	GREB1	growth regulating estrogen receptor binding 1	HGNC:24885	2p25.1
GRIN2A	Approved symbol	GRIN2A	glutamate ionotropic receptor NMDA type subunit 2A	HGNC:4585	16p13.2
GRM1	Approved symbol	GRM1	glutamate metabotropic receptor 1	HGNC:4593	6q24.3
HCFC1	Approved symbol	HCFC1	host cell factor C1	HGNC:4839	Xq28
HCN1	Approved symbol	HCN1	hyperpolarization activated cyclic nucleotide gated potassium channel 1	HGNC:4845	5p12
HDAC9	Approved symbol	HDAC9	histone deacetylase 9	HGNC:14065	7p21.1
HECTD4	Approved symbol	HECTD4	HECT domain E3 ubiquitin protein ligase 4	HGNC:26611	12q24.13
HECW1	Approved symbol	HECW1	HECT, C2 and WW domain containing E3 ubiquitin protein ligase 1	HGNC:22195	7p14.1-p13
HELZ2	Approved symbol	HELZ2	helicase with zinc finger 2	HGNC:30021	20q13.33
HERC1	Approved symbol	HERC1	HECT and RLD domain containing E3 ubiquitin protein ligase family member 1	HGNC:4867	15q22.31
HERC2	Approved symbol	HERC2	HECT and RLD domain containing E3 ubiquitin protein ligase 2	HGNC:4868	15q13.1
HIVEP2	Approved symbol	HIVEP2	HIVEP zinc finger 2	HGNC:4921	6q24.2
HIVEP3	Approved symbol	HIVEP3	HIVEP zinc finger 3	HGNC:13561	1p34.2
HMCN1	Approved symbol	HMCN1	hemicentin 1	HGNC:19194	1q25.3-q31.1
HRNR	Approved symbol	HRNR	hornerin	HGNC:20846	1q21.3
HSPG2	Approved symbol	HSPG2	heparan sulfate proteoglycan 2	HGNC:5273	1p36.12
HTT	Approved symbol	HTT	huntingtin	HGNC:4851	4p16.3
HUWE1	Approved symbol	HUWE1	HECT, UBA and WWE domain containing E3 ubiquitin protein ligase 1	HGNC:30892	Xp11.22
HYDIN	Approved symbol	HYDIN	HYDIN axonemal central pair apparatus protein	HGNC:19368	16q22.2
CHD3	Approved symbol	CHD3	chromodomain helicase DNA binding protein 3	HGNC:1918	17p13.1
CHD5	Approved symbol	CHD5	chromodomain helicase DNA binding protein 5	HGNC:16816	1p36.31
CHD6	Approved symbol	CHD6	chromodomain helicase DNA binding protein 6	HGNC:19057	20q12
CHD7	Approved symbol	CHD7	chromodomain helicase DNA binding protein 7	HGNC:20626	8q12.2
CHD9	Approved symbol	CHD9	chromodomain helicase DNA binding protein 9	HGNC:25701	16q12.2
ICE1	Approved symbol	ICE1	interactor of little elongation complex ELL subunit 1	HGNC:29154	5p15.32
IGF2R	Approved symbol	IGF2R	insulin like growth factor 2 receptor	HGNC:5467	6q25.3
IGSF10	Approved symbol	IGSF10	immunoglobulin superfamily member 10	HGNC:26384	3q25.1
INF2	Approved symbol	INF2	inverted formin 2	HGNC:23791	14q32.33

INSL6	Approved symbol	INSL6	insulin like 6	HGNC:6089	9p24.1
ITGB4	Approved symbol	ITGB4	integrin subunit beta 4	HGNC:6158	17q25.1
ITPR1	Approved symbol	ITPR1	inositol 1,4,5-trisphosphate receptor type 1	HGNC:6180	3p26.1
ITPR3	Approved symbol	ITPR3	inositol 1,4,5-trisphosphate receptor type 3	HGNC:6182	6p21.31
ITSN1	Approved symbol	ITSN1	intersectin 1	HGNC:6183	21q22.11
JARID2	Approved symbol	JARID2	jumonji and AT-rich interaction domain containing 2	HGNC:6196	6p22.3
JCAD	Approved symbol	JCAD	junctional cadherin 5 associated	HGNC:29283	10p11.23
KALRN	Approved symbol	KALRN	kalirin RhoGEF kinase	HGNC:4814	3q21.1-q21.2
KIAA0556	Previous symbol	KATNIP	katanin interacting protein	HGNC:29068	16p12.1
KCNMA1	Approved symbol	KCNMA1	potassium calcium-activated channel subfamily M alpha 1	HGNC:6284	10q22.3
KDM2B	Approved symbol	KDM2B	lysine demethylase 2B	HGNC:13610	12q24.31
KDM3B	Approved symbol	KDM3B	lysine demethylase 3B	HGNC:1337	5q31.2
KDR	Approved symbol	KDR	kinase insert domain receptor	HGNC:6307	4q12
KIAA1549	Approved symbol	KIAA1549	KIAA1549	HGNC:22219	7q34
KIAA1549L	Approved symbol	KIAA1549L	KIAA1549 like	HGNC:24836	11p13
KIF1A	Approved symbol	KIF1A	kinesin family member 1A	HGNC:888	2q37.3
KIF26B	Approved symbol	KIF26B	kinesin family member 26B	HGNC:25484	1q44
KIF2B	Approved symbol	KIF2B	kinesin family member 2B	HGNC:29443	17q22
KMT2B	Approved symbol	KMT2B	lysine methyltransferase 2B	HGNC:15840	19q13.12
KMT2C	Approved symbol	KMT2C	lysine methyltransferase 2C	HGNC:13726	7q36.1
KMT2D	Approved symbol	KMT2D	lysine methyltransferase 2D	HGNC:7133	12q13.12
KNDC1	Approved symbol	KNDC1	kinase non-catalytic C-lobe domain containing 1	HGNC:29374	10q26.3
KNL1	Approved symbol	KNL1	kinetochore scaffold 1	HGNC:24054	15q15.1
KRAS	Approved symbol	KRAS	KRAS proto-oncogene, GTPase	HGNC:6407	12p12.1
LAMA1	Approved symbol	LAMA1	laminin subunit alpha 1	HGNC:6481	18p11.31
LAMA2	Approved symbol	LAMA2	laminin subunit alpha 2	HGNC:6482	6q22.33
LAMA3	Approved symbol	LAMA3	laminin subunit alpha 3	HGNC:6483	18q11.2
LAMA4	Approved symbol	LAMA4	laminin subunit alpha 4	HGNC:6484	6q21
LAMA5	Approved symbol	LAMA5	laminin subunit alpha 5	HGNC:6485	20q13.33
LAMB2	Approved symbol	LAMB2	laminin subunit beta 2	HGNC:6487	3p21.31
LAMC1	Approved symbol	LAMC1	laminin subunit gamma 1	HGNC:6492	1q25.3
LARP4B	Approved symbol	LARP4B	La ribonucleoprotein 4B	HGNC:28987	10p15.3
LCT	Approved symbol	LCT	lactase	HGNC:6530	2q21.3
LIFR	Approved symbol	LIFR	LIF receptor subunit alpha	HGNC:6597	5p13.1
LRIF1	Approved symbol	LRIF1	ligand dependent nuclear receptor interacting factor 1	HGNC:30299	1p13.3
LRP1	Approved symbol	LRP1	LDL receptor related protein 1	HGNC:6692	12q13.3
LRP1B	Approved symbol	LRP1B	LDL receptor related protein 1B	HGNC:6693	2q22.1-q22.2
LRP2	Approved symbol	LRP2	LDL receptor related protein 2	HGNC:6694	2q31.1
LRRC7	Approved symbol	LRRC7	leucine rich repeat containing 7	HGNC:18531	1p31.1

LRRIQ1	Approved symbol	LRRIQ1	leucine rich repeats and IQ motif containing 1	HGNC:25708	12q21.31
LRRK2	Approved symbol	LRRK2	leucine rich repeat kinase 2	HGNC:18618	12q12
LTBP4	Approved symbol	LTBP4	latent transforming growth factor beta binding protein 4	HGNC:6717	19q13.2
LYST	Approved symbol	LYST	lysosomal trafficking regulator	HGNC:1968	1q42.3
MACF1	Approved symbol	MACF1	microtubule actin crosslinking factor 1	HGNC:13664	1p34.3
MAGEC1	Approved symbol	MAGEC1	MAGE family member C1	HGNC:6812	Xq27.2
MAGI1	Approved symbol	MAGI1	membrane associated guanylate kinase, WW and PDZ domain containing 1	HGNC:946	3p14.1
MAGI2	Approved symbol	MAGI2	membrane associated guanylate kinase, WW and PDZ domain containing 2	HGNC:18957	7q21.11
MAP1A	Approved symbol	MAP1A	microtubule associated protein 1A	HGNC:6835	15q15.3
MAP1B	Approved symbol	MAP1B	microtubule associated protein 1B	HGNC:6836	5q13.2
MAP2	Approved symbol	MAP2	microtubule associated protein 2	HGNC:6839	2q34
MAPRE2	Approved symbol	MAPRE2	microtubule associated protein RP/EB family member 2	HGNC:6891	18q12.1-q12.2
MDGA2	Approved symbol	MDGA2	MAM domain containing glycosylphosphatidylinositol anchor 2	HGNC:19835	14q21.3
MDN1	Approved symbol	MDN1	midasin AAA ATPase 1	HGNC:18302	6q15
MED12	Approved symbol	MED12	mediator complex subunit 12	HGNC:11957	Xq13.1
MED12L	Approved symbol	MED12L	mediator complex subunit 12L	HGNC:16050	3q25.1
MEGF6	Approved symbol	MEGF6	multiple EGF like domains 6	HGNC:3232	1p36.32
METAP1	Approved symbol	METAP1	methionyl aminopeptidase 1	HGNC:15789	4q23
METAP2	Approved symbol	METAP2	methionyl aminopeptidase 2	HGNC:16672	12q22
MGA	Approved symbol	MGA	MAX dimerization protein MGA	HGNC:14010	15q15
MGAM	Approved symbol	MGAM	maltase-glucoamylase	HGNC:7043	7q34
KIAA1024	Previous symbol	MINAR1	membrane integral NOTCH2 associated receptor 1	HGNC:29172	15q25.1
MKI67	Approved symbol	MKI67	marker of proliferation Ki-67	HGNC:7107	10q26.2
MN1	Approved symbol	MN1	MN1 proto-oncogene, transcriptional regulator	HGNC:7180	22q12.1
MSH6	Approved symbol	MSH6	mutS homolog 6	HGNC:7329	2p16.3
MTOR	Approved symbol	MTOR	mechanistic target of rapamycin kinase	HGNC:3942	1p36.22
MUC16	Approved symbol	MUC16	mucin 16, cell surface associated	HGNC:15582	19p13.2
MUC17	Approved symbol	MUC17	mucin 17, cell surface associated	HGNC:16800	7q22.1
MUC5AC	Approved symbol	MUC5AC	mucin 5AC, oligomeric mucus/gel-forming	HGNC:7515	11p15.5
MUC5B	Approved symbol	MUC5B	mucin 5B, oligomeric mucus/gel-forming	HGNC:7516	11p15.5
MUC6	Approved symbol	MUC6	mucin 6, oligomeric mucus/gel-forming	HGNC:7517	11p15.5
MXRA5	Approved symbol	MXRA5	matrix remodeling associated 5	HGNC:7539	Xp22.33
MYCBP2	Approved symbol	MYCBP2	MYC binding protein 2	HGNC:23386	13q22.3
MYH11	Approved symbol	MYH11	myosin heavy chain 11	HGNC:7569	16p13.11
MYH13	Approved symbol	MYH13	myosin heavy chain 13	HGNC:7571	17p13.1
MYH6	Approved symbol	MYH6	myosin heavy chain 6	HGNC:7576	14q11.2
MYH7	Approved symbol	MYH7	myosin heavy chain 7	HGNC:7577	14q11.2
MYH8	Approved symbol	MYH8	myosin heavy chain 8	HGNC:7578	17p13.1
MYO10	Approved symbol	MYO10	myosin X	HGNC:7593	5p15.1

MYO15A	Approved symbol	MYO15A	myosin XVA	HGNC:7594	17p11.2
MYO16	Approved symbol	MYO16	myosin XVI	HGNC:29822	13q33.3
MYO18B	Approved symbol	MYO18B	myosin XVIIIB	HGNC:18150	22q12.1
MYO7B	Approved symbol	MYO7B	myosin VIIB	HGNC:7607	2q14.3
MYT1L	Approved symbol	MYT1L	myelin transcription factor 1 like	HGNC:7623	2p25.3
NALCN	Approved symbol	NALCN	sodium leak channel, non-selective	HGNC:19082	13q32.3-q33.1
NANOS1	Approved symbol	NANOS1	nanos C2HC-type zinc finger 1	HGNC:23044	10q26.11
NAV2	Approved symbol	NAV2	neuron navigator 2	HGNC:15997	11p15.1
NAV3	Approved symbol	NAV3	neuron navigator 3	HGNC:15998	12q21.2
NBEA	Approved symbol	NBEA	neurobeachin	HGNC:7648	13q13.3
NBEAL2	Approved symbol	NBEAL2	neurobeachin like 2	HGNC:31928	3p21.31
NCAN	Approved symbol	NCAN	neurocan	HGNC:2465	19p12
NCKAP5	Approved symbol	NCKAP5	NCK associated protein 5	HGNC:29847	2q21.2
NCOR1	Approved symbol	NCOR1	nuclear receptor corepressor 1	HGNC:7672	17p12-p11.2
NCOR2	Approved symbol	NCOR2	nuclear receptor corepressor 2	HGNC:7673	12q24.31
NEB	Approved symbol	NEB	nebulin	HGNC:7720	2q23.3
NF1	Approved symbol	NF1	neurofibromin 1	HGNC:7765	17q11.2
NFASC	Approved symbol	NFASC	neurofascin	HGNC:29866	1q32.1
NHS	Approved symbol	NHS	NHS actin remodeling regulator	HGNC:7820	Xp22.2-p22.13
NID1	Approved symbol	NID1	nidogen 1	HGNC:7821	1q42.3
NIN	Approved symbol	NIN	ninein	HGNC:14906	14q22.1
NIPBL	Approved symbol	NIPBL	NIPBL cohesin loading factor	HGNC:28862	5p13.2
NLRP12	Approved symbol	NLRP12	NLR family pyrin domain containing 12	HGNC:22938	19q13.42
NLRP4	Approved symbol	NLRP4	NLR family pyrin domain containing 4	HGNC:22943	19q13.43
NLRP5	Approved symbol	NLRP5	NLR family pyrin domain containing 5	HGNC:21269	19q13.43
NLRP7	Approved symbol	NLRP7	NLR family pyrin domain containing 7	HGNC:22947	19q13.42
NOS1	Approved symbol	NOS1	nitric oxide synthase 1	HGNC:7872	12q24.22
NOTCH1	Approved symbol	NOTCH1	notch receptor 1	HGNC:7881	9q34.3
NOTCH2	Approved symbol	NOTCH2	notch receptor 2	HGNC:7882	1p12
NOTCH3	Approved symbol	NOTCH3	notch receptor 3	HGNC:7883	19p13.12
NPAP1	Approved symbol	NPAP1	nuclear pore associated protein 1	HGNC:1190	15q11.2
NRAS	Approved symbol	NRAS	NRAS proto-oncogene, GTPase	HGNC:7989	1p13.2
NRXN1	Approved symbol	NRXN1	neurexin 1	HGNC:8008	2p16.3
NRXN3	Approved symbol	NRXN3	neurexin 3	HGNC:8010	14q24.3-q31.1
NUP205	Approved symbol	NUP205	nucleoporin 205	HGNC:18658	7q33
NWD1	Approved symbol	NWD1	NACHT and WD repeat domain containing 1	HGNC:27619	19p13.11
NYNRIN	Approved symbol	NYNRIN	NYN domain and retroviral integrase containing	HGNC:20165	14q12
OBSCN	Approved symbol	OBSCN	obscurin, cytoskeletal calmodulin and titin-interacting RhoGEF	HGNC:15719	1q42.13
OTOF	Approved symbol	OTOF	otoferlin	HGNC:8515	2p23.3

OTOGL	Approved symbol	OTOGL	otogelin like	HGNC:26901	12q21.31
PAPPA	Approved symbol	PAPPA	pappalysin 1	HGNC:8602	9q33.1
PAPPA2	Approved symbol	PAPPA2	pappalysin 2	HGNC:14615	1q25.2
PCDH10	Approved symbol	PCDH10	protocadherin 10	HGNC:13404	4q28.3
PCDH15	Approved symbol	PCDH15	protocadherin related 15	HGNC:14674	10q21.1
PCDH17	Approved symbol	PCDH17	protocadherin 17	HGNC:14267	13q21.1
PCDH19	Approved symbol	PCDH19	protocadherin 19	HGNC:14270	Xq22.1
PCDHA1	Approved symbol	PCDHA1	protocadherin alpha 1	HGNC:8663	5q31.3
PCDHA11	Approved symbol	PCDHA11	protocadherin alpha 11	HGNC:8665	5q31.3
PCDHA12	Approved symbol	PCDHA12	protocadherin alpha 12	HGNC:8666	5q31.3
PCDHA13	Approved symbol	PCDHA13	protocadherin alpha 13	HGNC:8667	5q31.3
PCDHA2	Approved symbol	PCDHA2	protocadherin alpha 2	HGNC:8668	5q31.3
PCDHA3	Approved symbol	PCDHA3	protocadherin alpha 3	HGNC:8669	5q31.3
PCDHA6	Approved symbol	PCDHA6	protocadherin alpha 6	HGNC:8672	5q31.3
PCDHAC1	Approved symbol	PCDHAC1	protocadherin alpha subfamily C, 1	HGNC:8676	5q31.3
PCDHB3	Approved symbol	PCDHB3	protocadherin beta 3	HGNC:8688	5q31.3
PCDHB7	Approved symbol	PCDHB7	protocadherin beta 7	HGNC:8692	5q31.3
PCDHGA1	Approved symbol	PCDHGA1	protocadherin gamma subfamily A, 1	HGNC:8696	5q31
PCDHGA2	Approved symbol	PCDHGA2	protocadherin gamma subfamily A, 2	HGNC:8700	5q31.3
PCDHGA5	Approved symbol	PCDHGA5	protocadherin gamma subfamily A, 5	HGNC:8703	5q31
PCDHGA8	Approved symbol	PCDHGA8	protocadherin gamma subfamily A, 8	HGNC:8706	5q31.3
PCDHGB1	Approved symbol	PCDHGB1	protocadherin gamma subfamily B, 1	HGNC:8708	5q31
PCDHGB4	Approved symbol	PCDHGB4	protocadherin gamma subfamily B, 4	HGNC:8711	5q31
PCLO	Approved symbol	PCLO	piccolo presynaptic cytomatrix protein	HGNC:13406	7q21.11
PCNT	Approved symbol	PCNT	pericentrin	HGNC:16068	21q22.3
PDE4DIP	Approved symbol	PDE4DIP	phosphodiesterase 4D interacting protein	HGNC:15580	1q21.2
PDZD2	Approved symbol	PDZD2	PDZ domain containing 2	HGNC:18486	5p13.3
PEG3	Approved symbol	PEG3	paternally expressed 3	HGNC:8826	19q13.43
PHF2	Approved symbol	PHF2	PHD finger protein 2	HGNC:8920	9q22.31
PHRF1	Approved symbol	PHRF1	PHD and ring finger domains 1	HGNC:24351	11p15.5
PIK3CA	Approved symbol	PIK3CA	phosphatidylinositol-4,5-bisphosphate 3-kinase catalytic subunit alpha	HGNC:8975	3q26.32
PIK3CG	Approved symbol	PIK3CG	phosphatidylinositol-4,5-bisphosphate 3-kinase catalytic subunit gamma	HGNC:8978	7q22.3
PIK3R1	Approved symbol	PIK3R1	phosphoinositide-3-kinase regulatory subunit 1	HGNC:8979	5q13.1
PKD1	Approved symbol	PKD1	polycystin 1, transient receptor potential channel interacting	HGNC:9008	16p13.3
PKD1L1	Approved symbol	PKD1L1	polycystin 1 like 1, transient receptor potential channel interacting	HGNC:18053	7p12.3
PKDREJ	Approved symbol	PKDREJ	polycystin family receptor for egg jelly	HGNC:9015	22q13.31
PKHD1	Approved symbol	PKHD1	PKHD1 ciliary IPT domain containing fibrocystin/polyductin	HGNC:9016	6p12.3-p12.2
PKHD1L1	Approved symbol	PKHD1L1	PKHD1 like 1	HGNC:20313	8q23.1-q23.2
PLCB1	Approved symbol	PLCB1	phospholipase C beta 1	HGNC:15917	20p12.3

PLCG2	Approved symbol	PLCG2	phospholipase C gamma 2	HGNC:9066	16q24.1
PLEC	Approved symbol	PLEC	plectin	HGNC:9069	8q24.3
PLEKHA6	Approved symbol	PLEKHA6	pleckstrin homology domain containing A6	HGNC:17053	1q32.1
PLEKHG4B	Approved symbol	PLEKHG4B	pleckstrin homology and RhoGEF domain containing G4B	HGNC:29399	5p15.33
PLXNA1	Approved symbol	PLXNA1	plexin A1	HGNC:9099	3q21.3
PLXNA2	Approved symbol	PLXNA2	plexin A2	HGNC:9100	1q32.2
PLXNA3	Approved symbol	PLXNA3	plexin A3	HGNC:9101	Xq28
PLXNA4	Approved symbol	PLXNA4	plexin A4	HGNC:9102	7q32.3
PLXNB2	Approved symbol	PLXNB2	plexin B2	HGNC:9104	22q13.33
PLXNB3	Approved symbol	PLXNB3	plexin B3	HGNC:9105	Xq28
POLE	Approved symbol	POLE	DNA polymerase epsilon, catalytic subunit	HGNC:9177	12q24.33
POLK	Approved symbol	POLK	DNA polymerase kappa	HGNC:9183	5q13.3
POLQ	Approved symbol	POLQ	DNA polymerase theta	HGNC:9186	3q13.33
PRDM2	Approved symbol	PRDM2	PR/SET domain 2	HGNC:9347	1p36.21
PREX2	Approved symbol	PREX2	phosphatidylinositol-3,4,5-trisphosphate dependent Rac exchange factor 2	HGNC:22950	8q13.2
PRRC2B	Approved symbol	PRRC2B	proline rich coiled-coil 2B	HGNC:28121	9q34.13
PRUNE2	Approved symbol	PRUNE2	prune homolog 2 with BCH domain	HGNC:25209	9q21.2
PTEN	Approved symbol	PTEN	phosphatase and tensin homolog	HGNC:9588	10q23.31
PTCH1	Approved symbol	PTCH1	patched 1	HGNC:9585	9q22.32
PTPRK	Approved symbol	PTPRK	protein tyrosine phosphatase receptor type K	HGNC:9674	6q22.33
PTPRS	Approved symbol	PTPRS	protein tyrosine phosphatase receptor type S	HGNC:9681	19p13.3
PTPRT	Approved symbol	PTPRT	protein tyrosine phosphatase receptor type T	HGNC:9682	20q12-q13.11
PTPRU	Approved symbol	PTPRU	protein tyrosine phosphatase receptor type U	HGNC:9683	1p35.3
PTPRZ1	Approved symbol	PTPRZ1	protein tyrosine phosphatase receptor type Z1	HGNC:9685	7q31.32
PXDN	Approved symbol	PXDN	peroxidasin	HGNC:14966	2p25.3
PXDNL	Approved symbol	PXDNL	peroxidasin like	HGNC:26359	8q11.22-q11.23
RAI1	Approved symbol	RAI1	retinoic acid induced 1	HGNC:9834	17p11.2
RALGAPA1	Approved symbol	RALGAPA1	Ral GTPase activating protein catalytic subunit alpha 1	HGNC:17770	14q13.2
RALGAPA2	Approved symbol	RALGAPA2	Ral GTPase activating protein catalytic subunit alpha 2	HGNC:16207	20p11.23
RASGRF1	Approved symbol	RASGRF1	Ras protein specific guanine nucleotide releasing factor 1	HGNC:9875	15q25.1
RELN	Approved symbol	RELN	reelin	HGNC:9957	7q22.1
REV3L	Approved symbol	REV3L	REV3 like, DNA directed polymerase zeta catalytic subunit	HGNC:9968	6q21
RGS12	Approved symbol	RGS12	regulator of G protein signaling 12	HGNC:9994	4p16.3
RGS22	Approved symbol	RGS22	regulator of G protein signaling 22	HGNC:24499	8q22.2
RIF1	Approved symbol	RIF1	replication timing regulatory factor 1	HGNC:23207	2q23.3
RIMBP2	Approved symbol	RIMBP2	RIMS binding protein 2	HGNC:30339	12q24.33
RIMS1	Approved symbol	RIMS1	regulating synaptic membrane exocytosis 1	HGNC:17282	6q13
RIMS2	Approved symbol	RIMS2	regulating synaptic membrane exocytosis 2	HGNC:17283	8q22.3
RNASE12	Approved symbol	RNASE12	ribonuclease A family member 12 (inactive)	HGNC:24211	14q11.2

RNF213	Approved symbol	RNF213	ring finger protein 213	HGNC:14539	17q25.3
RNF43	Approved symbol	RNF43	ring finger protein 43	HGNC:18505	17q22
ROBO1	Approved symbol	ROBO1	roundabout guidance receptor 1	HGNC:10249	3p12.3
ROBO2	Approved symbol	ROBO2	roundabout guidance receptor 2	HGNC:10250	3p12.3
ROBO3	Approved symbol	ROBO3	roundabout guidance receptor 3	HGNC:13433	11q24.2
RP1	Approved symbol	RP1	RP1 axonemal microtubule associated	HGNC:10263	8q11.23-q12.1
RP1L1	Approved symbol	RP1L1	RP1 like 1	HGNC:15946	8p23.1
RREB1	Approved symbol	RREB1	ras responsive element binding protein 1	HGNC:10449	6p24.3
RYR1	Approved symbol	RYR1	ryanodine receptor 1	HGNC:10483	19q13.2
RYR2	Approved symbol	RYR2	ryanodine receptor 2	HGNC:10484	1q43
RYR3	Approved symbol	RYR3	ryanodine receptor 3	HGNC:10485	15q13.3-q14
SACS	Approved symbol	SACS	sacsin molecular chaperone	HGNC:10519	13q12.12
SALL1	Approved symbol	SALL1	spalt like transcription factor 1	HGNC:10524	16q12.1
SBF1	Approved symbol	SBF1	SET binding factor 1	HGNC:10542	22q13.33
SCLT1	Approved symbol	SCLT1	sodium channel and clathrin linker 1	HGNC:26406	4q28.2
SCN10A	Approved symbol	SCN10A	sodium voltage-gated channel alpha subunit 10	HGNC:10582	3p22.2
SCN11A	Approved symbol	SCN11A	sodium voltage-gated channel alpha subunit 11	HGNC:10583	3p22.2
SCN1A	Approved symbol	SCN1A	sodium voltage-gated channel alpha subunit 1	HGNC:10585	2q24.3
SCN2A	Approved symbol	SCN2A	sodium voltage-gated channel alpha subunit 2	HGNC:10588	2q24.3
SCN3A	Approved symbol	SCN3A	sodium voltage-gated channel alpha subunit 3	HGNC:10590	2q24.3
SCN4A	Approved symbol	SCN4A	sodium voltage-gated channel alpha subunit 4	HGNC:10591	17q23.3
SCN5A	Approved symbol	SCN5A	sodium voltage-gated channel alpha subunit 5	HGNC:10593	3p22.2
SCN7A	Approved symbol	SCN7A	sodium voltage-gated channel alpha subunit 7	HGNC:10594	2q24.3
SCN9A	Approved symbol	SCN9A	sodium voltage-gated channel alpha subunit 9	HGNC:10597	2q24.3
SDK1	Approved symbol	SDK1	sidekick cell adhesion molecule 1	HGNC:19307	7p22.2
SDK2	Approved symbol	SDK2	sidekick cell adhesion molecule 2	HGNC:19308	17q25.1
SEC16A	Approved symbol	SEC16A	SEC16 homolog A, endoplasmic reticulum export factor	HGNC:29006	9q34.3
SETBP1	Approved symbol	SETBP1	SET binding protein 1	HGNC:15573	18q12.3
SETX	Approved symbol	SETX	senataxin	HGNC:445	9q34.13
SHANK1	Approved symbol	SHANK1	SH3 and multiple ankyrin repeat domains 1	HGNC:15474	19q13.33
SIN3B	Approved symbol	SIN3B	SIN3 transcription regulator family member B	HGNC:19354	19p13.11
SLC6A4	Approved symbol	SLC6A4	solute carrier family 6 member 4	HGNC:11050	17q11.2
SLIT1	Approved symbol	SLIT1	slit guidance ligand 1	HGNC:11085	10q24.1
SLIT2	Approved symbol	SLIT2	slit guidance ligand 2	HGNC:11086	4p15.31
SLIT3	Approved symbol	SLIT3	slit guidance ligand 3	HGNC:11087	5q34-q35.1
SLITRK1	Approved symbol	SLITRK1	SLIT and NTRK like family member 1	HGNC:20297	13q31.1
SLITRK5	Approved symbol	SLITRK5	SLIT and NTRK like family member 5	HGNC:20295	13q31.2
SLX4	Approved symbol	SLX4	SLX4 structure-specific endonuclease subunit	HGNC:23845	16p13.3

SMARCA4	Approved symbol	SMARCA4	SWI/SNF related, matrix associated, actin dependent regulator of chromatin, subfamily a, member 4	HGNC:11100	19p13.2
SMG1	Approved symbol	SMG1	SMG1 nonsense mediated mRNA decay associated PI3K related kinase	HGNC:30045	16p12.3
SNRNP200	Approved symbol	SNRNP200	small nuclear ribonucleoprotein U5 subunit 200	HGNC:30859	2q11.2
SPATA31D1	Approved symbol	SPATA31D1	SPATA31 subfamily D member 1	HGNC:37283	9q21.32
SPECC1	Approved symbol	SPECC1	sperm antigen with calponin homology and coiled-coil domains 1	HGNC:30615	17p11.2
SPEG	Approved symbol	SPEG	striated muscle enriched protein kinase	HGNC:16901	2q35
SPEN	Approved symbol	SPEN	spen family transcriptional repressor	HGNC:17575	1p36.21-p36.13
SPHKAP	Approved symbol	SPHKAP	SPHK1 interactor, AKAP domain containing	HGNC:30619	2q36.3
SPTA1	Approved symbol	SPTA1	spectrin alpha, erythrocytic 1	HGNC:11272	1q23.1
SPTB	Approved symbol	SPTB	spectrin beta, erythrocytic	HGNC:11274	14q23.3
SPTBN1	Approved symbol	SPTBN1	spectrin beta, non-erythrocytic 1	HGNC:11275	2p16.2
SPTBN2	Approved symbol	SPTBN2	spectrin beta, non-erythrocytic 2	HGNC:11276	11q13.2
SRCAP	Approved symbol	SRCAP	Snf2 related CREBBP activator protein	HGNC:16974	16p11.2
SRRM2	Approved symbol	SRRM2	serine/arginine repetitive matrix 2	HGNC:16639	16p13.3
STAB1	Approved symbol	STAB1	stabilin 1	HGNC:18628	3p21.1
STAB2	Approved symbol	STAB2	stabilin 2	HGNC:18629	12q23.3
STK19	Approved symbol	STK19	serine/threonine kinase 19	HGNC:11398	6p21.33
SVEP1	Approved symbol	SVEP1	sushi, von Willebrand factor type A, EGF and pentraxin domain containing 1	HGNC:15985	9q31.3
SVIL	Approved symbol	SVIL	supervillin	HGNC:11480	10p11.23
SYNE1	Approved symbol	SYNE1	spectrin repeat containing nuclear envelope protein 1	HGNC:17089	6q25.2
SYNE2	Approved symbol	SYNE2	spectrin repeat containing nuclear envelope protein 2	HGNC:17084	14q23.2
SZT2	Approved symbol	SZT2	SZT2 subunit of KICSTOR complex	HGNC:29040	1p34.2
TAF1L	Approved symbol	TAF1L	TATA-box binding protein associated factor 1 like	HGNC:18056	9p21.1
TAOK2	Approved symbol	TAOK2	TAO kinase 2	HGNC:16835	16p11.2
TECTA	Approved symbol	TECTA	tectorin alpha	HGNC:11720	11q23.3
TENM1	Approved symbol	TENM1	teneurin transmembrane protein 1	HGNC:8117	Xq25
TENM2	Approved symbol	TENM2	teneurin transmembrane protein 2	HGNC:29943	5q34
TENM3	Approved symbol	TENM3	teneurin transmembrane protein 3	HGNC:29944	4q34.3-q35.1
TENM4	Approved symbol	TENM4	teneurin transmembrane protein 4	HGNC:29945	11q14.1
PAPD7	Previous symbol	TENT4A	terminal nucleotidyltransferase 4A	HGNC:16705	5p15.31
TEP1	Approved symbol	TEP1	telomerase associated protein 1	HGNC:11726	14q11.2
TEX15	Approved symbol	TEX15	testis expressed 15, meiosis and synapsis associated	HGNC:11738	8p12
TG	Approved symbol	TG	thyroglobulin	HGNC:11764	8q24.22
TCHH	Approved symbol	TCHH	trichohyalin	HGNC:11791	1q21.3
TIAM1	Approved symbol	TIAM1	TIAM Rac1 associated GEF 1	HGNC:11805	21q22.11
TLN2	Approved symbol	TLN2	talin 2	HGNC:15447	15q22.2
TMEM131	Approved symbol	TMEM131	transmembrane protein 131	HGNC:30366	2q11.2
TMEM132D	Approved symbol	TMEM132D	transmembrane protein 132D	HGNC:29411	12q24.33

TNC	Approved symbol	TNC	tenascin C	HGNC:5318	9q33.1
TNN	Approved symbol	TNN	tenascin N	HGNC:22942	1q25.1
TNR	Approved symbol	TNR	tenascin R	HGNC:11953	1q25.1
TNRC18	Approved symbol	TNRC18	trinucleotide repeat containing 18	HGNC:11962	7p22.1
TNRC6A	Approved symbol	TNRC6A	trinucleotide repeat containing adaptor 6A	HGNC:11969	16p12.1
TNRC6B	Approved symbol	TNRC6B	trinucleotide repeat containing adaptor 6B	HGNC:29190	22q13.1
TNXB	Approved symbol	TNXB	tenascin XB	HGNC:11976	6p21.33-p21.32
TP53	Approved symbol	TP53	tumor protein p53	HGNC:11998	17p13.1
TPR	Approved symbol	TPR	translocated promoter region, nuclear basket protein	HGNC:12017	1q31.1
TRIO	Approved symbol	TRIO	trio Rho guanine nucleotide exchange factor	HGNC:12303	5p15.2
TRIOBP	Approved symbol	TRIOBP	TRIO and F-actin binding protein	HGNC:17009	22q13.1
TRPC4	Approved symbol	TRPC4	transient receptor potential cation channel subfamily C member 4	HGNC:12336	13q13.3
TRPM2	Approved symbol	TRPM2	transient receptor potential cation channel subfamily M member 2	HGNC:12339	21q22.3
TRPM3	Approved symbol	TRPM3	transient receptor potential cation channel subfamily M member 3	HGNC:17992	9q21.12-q21.13
TRPS1	Approved symbol	TRPS1	transcriptional repressor GATA binding 1	HGNC:12340	8q23.3
TRRAP	Approved symbol	TRRAP	transformation/transcription domain associated protein	HGNC:12347	7q22.1
TSC2	Approved symbol	TSC2	TSC complex subunit 2	HGNC:12363	16p13.3
TSHZ3	Approved symbol	TSHZ3	teashirt zinc finger homeobox 3	HGNC:30700	19q12
TTK	Approved symbol	TTK	TTK protein kinase	HGNC:12401	6q14.1
TTN	Approved symbol	TTN	titin	HGNC:12403	2q31.2
UBR4	Approved symbol	UBR4	ubiquitin protein ligase E3 component n-recognin 4	HGNC:30313	1p36.13
UBR5	Approved symbol	UBR5	ubiquitin protein ligase E3 component n-recognin 5	HGNC:16806	8q22.3
UNC13A	Approved symbol	UNC13A	unc-13 homolog A	HGNC:23150	19p13.11
UNC13C	Approved symbol	UNC13C	unc-13 homolog C	HGNC:23149	15q21.3
UNC79	Approved symbol	UNC79	unc-79 homolog, NALCN channel complex subunit	HGNC:19966	14q32.12
UNC80	Approved symbol	UNC80	unc-80 homolog, NALCN channel complex subunit	HGNC:26582	2q34
USH2A	Approved symbol	USH2A	usherin	HGNC:12601	1q41
USP34	Approved symbol	USP34	ubiquitin specific peptidase 34	HGNC:20066	2p15
USP35	Approved symbol	USP35	ubiquitin specific peptidase 35	HGNC:20061	11q14.1
UTP20	Approved symbol	UTP20	UTP20 small subunit processome component	HGNC:17897	12q23.2
UTRN	Approved symbol	UTRN	utrophin	HGNC:12635	6q24.2
VCAN	Approved symbol	VCAN	versican	HGNC:2464	5q14.2-q14.3
VPS13A	Approved symbol	VPS13A	vacuolar protein sorting 13 homolog A	HGNC:1908	9q21.2
VPS13B	Approved symbol	VPS13B	vacuolar protein sorting 13 homolog B	HGNC:2183	8q22.2
VWF	Approved symbol	VWF	von Willebrand factor	HGNC:12726	12p13.31
WDFY3	Approved symbol	WDFY3	WD repeat and FYVE domain containing 3	HGNC:20751	4q21.23
WDFY4	Approved symbol	WDFY4	WDFY family member 4	HGNC:29323	10q11.23
WNK1	Approved symbol	WNK1	WNK lysine deficient protein kinase 1	HGNC:14540	12p13.33
WNK2	Approved symbol	WNK2	WNK lysine deficient protein kinase 2	HGNC:14542	9q22.31

XIRP2	Approved symbol	XIRP2	xin actin binding repeat containing 2	HGNC:14303	2q24.3
XYLT2	Approved symbol	XYLT2	xylosyltransferase 2	HGNC:15517	17q21.33
YLPM1	Approved symbol	YLPM1	YLP motif containing 1	HGNC:17798	14q24.3
ZC3H13	Approved symbol	ZC3H13	zinc finger CCCH-type containing 13	HGNC:20368	13q14.13
ZDBF2	Approved symbol	ZDBF2	zinc finger DBF-type containing 2	HGNC:29313	2q33.3
ZFHX3	Approved symbol	ZFHX3	zinc finger homeobox 3	HGNC:777	16q22.2-q22.3
ZFHX4	Approved symbol	ZFHX4	zinc finger homeobox 4	HGNC:30939	8q21.13
ZIM2	Approved symbol	ZIM2	zinc finger imprinted 2	HGNC:12875	19q13.43
ZNF106	Approved symbol	ZNF106	zinc finger protein 106	HGNC:12886	15q15.1
ZNF142	Approved symbol	ZNF142	zinc finger protein 142	HGNC:12927	2q35
ZNF318	Approved symbol	ZNF318	zinc finger protein 318	HGNC:13578	6p21.1
ZNF407	Approved symbol	ZNF407	zinc finger protein 407	HGNC:19904	18q23
ZNF423	Approved symbol	ZNF423	zinc finger protein 423	HGNC:16762	16q12.1
ZNF462	Approved symbol	ZNF462	zinc finger protein 462	HGNC:21684	9q31.2
ZNF536	Approved symbol	ZNF536	zinc finger protein 536	HGNC:29025	19q12
ZNF804A	Approved symbol	ZNF804A	zinc finger protein 804A	HGNC:21711	2q32.1
ZNF831	Approved symbol	ZNF831	zinc finger protein 831	HGNC:16167	20q13.32

Supplementary Table S2: Summary of somatic mutations in individual genes

Hugo_Symbol	Frame_Shift_ Del	Frame_Shift_ Ins	In_Frame_ Del	In_Frame _Ins	Missense_ Mutation	Nonsense_ Mutation	Splice_ Site	total	Amp	Del	CNV_total	Mutated Samples	Percetage	Altered Samples (mutations including CNVs)
APC	23	15	0	0	3	36	6	83	5	10	15	53	64	56
TP53	4	1	0	2	40	4	3	54	3	29	32	49	59	55
KRAS	0	0	0	0	35	0	0	35	7	1	8	35	42	39
FAT4	2	0	0	0	19	0	0	21	1	10	11	19	23	27
FBXW7	0	2	0	0	8	4	1	15	1	8	9	13	16	22
PIK3CA	0	0	1	0	13	0	0	14	5	1	6	13	16	18
CSMD3	1	1	0	0	11	0	0	13	24	2	26	12	14	36
RYR1	2	1	0	0	14	1	0	18	7	6	13	11	13	22
GLI3	2	0	0	0	13	0	0	15	27	0	27	11	13	35
TENM4	0	1	0	0	12	0	0	13	1	3	4	11	13	15
ABCA12	1	0	0	0	10	0	0	11	3	1	4	11	13	15
RYR2	2	0	0	0	12	0	0	14	10	0	10	10	12	18
LRP1B	0	1	0	0	11	1	0	13	4	2	6	10	12	15
XIRP2	2	2	0	0	8	1	0	13	5	0	5	10	12	14
COL7A1	1	0	0	0	11	0	0	12	2	1	3	10	12	13
PCLO	2	0	0	0	7	3	0	12	25	2	27	10	12	34
KIF1A	0	0	0	0	9	0	1	10	3	1	4	10	12	12
FREM2	3	0	0	0	9	0	0	12	30	1	31	9	11	38
KMT2D	6	0	0	0	6	0	0	12	8	1	9	9	11	17
VCAN	0	0	0	0	10	1	1	12	6	7	13	9	11	22
ZFHX4	2	2	0	0	8	0	0	12	20	3	23	9	11	30
DCHS1	2	0	0	0	7	2	0	11	3	3	6	9	11	15
LAMA1	2	0	0	0	9	0	0	11	3	25	28	9	11	37
DNAH5	1	1	0	0	8	0	0	10	11	2	13	9	11	22
MXRA5	0	0	1	0	9	0	0	10	12	33	45	9	11	48
COL4A1	0	0	0	0	8	0	1	9	29	1	30	9	11	35
CSMD2	0	0	0	0	9	0	0	9	0	8	8	9	11	17
EP300	1	3	0	0	6	1	0	11	0	14	14	8	10	22
NRXN1	2	0	0	0	9	0	0	11	6	4	10	8	10	17
ADGRB1	1	0	0	0	9	0	0	10	25	3	28	8	10	33
ARID1A	3	0	0	0	2	5	0	10	0	10	10	8	10	18
PCNT	1	2	0	0	7	0	0	10	1	12	13	8	10	21
SMG1	0	0	0	0	8	1	1	10	7	1	8	8	10	16

ZFHX3	1	1	0	0	8	0	0	10	14	2	16	8	10	24
ANK3	2	0	0	0	5	2	0	9	2	1	3	8	10	11
ATM	1	0	0	0	7	1	0	9	2	4	6	8	10	14
HYDIN	0	0	1	0	7	1	0	9	27	1	28	8	10	34
PKHD1	0	0	0	0	6	3	0	9	8	0	8	8	10	16
ROBO2	1	1	0	0	6	1	0	9	3	0	3	8	10	11
SACS	1	1	0	0	6	1	0	9	31	0	31	8	10	35
ADAMTS16	0	0	0	0	8	0	0	8	11	3	14	8	10	22
COL11A1	2	0	0	0	5	0	1	8	0	1	1	8	10	9
SHANK1	1	0	0	0	6	1	0	8	7	6	13	8	10	19
COL12A1	1	0	0	0	9	1	0	11	4	3	7	7	8	14
CREBBP	3	0	0	0	7	1	0	11	7	3	10	7	8	15
CSMD1	0	0	0	0	9	1	0	10	8	18	26	7	8	32
MDN1	3	0	0	0	6	1	0	10	4	3	7	7	8	14
CELSR3	3	0	0	0	6	0	0	9	2	1	3	7	8	9
DIDO1	2	0	0	0	7	0	0	9	39	5	44	7	8	49
DOCK3	2	1	0	0	3	3	0	9	2	1	3	7	8	8
FAT2	0	2	0	0	7	0	0	9	3	3	6	7	8	12
HECTD4	3	0	0	0	5	0	1	9	8	0	8	7	8	13
PHF2	8	0	0	0	1	0	0	9	8	2	10	7	8	17
DMD	0	0	0	0	7	0	1	8	11	32	43	7	8	49
DNAH7	2	0	0	0	4	2	0	8	6	1	7	7	8	14
FBN1	1	0	0	0	6	1	0	8	0	15	15	7	8	21
FN1	1	0	0	0	7	0	0	8	3	1	4	7	8	11
NAV3	0	0	0	0	8	0	0	8	8	2	10	7	8	15
PXDN	2	0	0	0	4	2	0	8	6	2	8	7	8	14
RYR3	0	0	0	0	8	0	0	8	0	28	28	7	8	35
TENM3	1	1	0	0	6	0	0	8	1	11	12	7	8	19
ZNF831	1	0	0	0	7	0	0	8	39	5	44	7	8	48
ABCA13	0	0	0	0	7	0	0	7	29	0	29	7	8	32
AKAP9	2	0	0	0	4	1	0	7	26	2	28	7	8	33
ANK2	0	0	0	0	7	0	0	7	1	10	11	7	8	18
CACNA1B	0	0	0	0	7	0	0	7	6	4	10	7	8	16
ITPR3	0	0	0	0	7	0	0	7	8	1	9	7	8	16
NOS1	1	3	0	0	2	1	0	7	7	0	7	7	8	11
PAPPA2	0	0	0	0	7	0	0	7	9	0	9	7	8	15
PCDH17	0	0	0	0	7	0	0	7	28	2	30	7	8	32
PCDHA12	0	0	1	0	6	0	0	7	0	0	0	7	8	7
SPEN	0	0	0	0	5	2	0	7	0	8	8	7	8	15

TMEM132D	1	1	0	0	5	0	0	7	8	1	9	7	8	14
TNRC18	1	0	1	0	5	0	0	7	27	1	28	7	8	34
ZNF536	0	0	0	0	7	0	0	7	7	2	9	7	8	14
KIAA1109	1	1	0	0	8	0	0	10	2	9	11	6	7	17
KMT2B	6	2	0	0	2	0	0	10	8	4	12	6	7	18
CACNA1E	0	0	0	0	9	0	0	9	11	0	11	6	7	17
TRRAP	2	0	0	0	7	0	0	9	24	2	26	6	7	30
TTK	5	0	0	0	3	0	1	9	4	2	6	6	7	12
CASZ1	0	0	0	0	7	0	1	8	1	6	7	6	7	12
CDH23	0	0	0	0	7	1	0	8	1	4	5	6	7	10
DCHS2	0	0	0	0	8	0	0	8	2	8	10	6	7	16
DNAH1	1	0	0	0	7	0	0	8	2	1	3	6	7	9
DOCK2	0	0	0	0	8	0	0	8	2	4	6	6	7	11
PREX2	1	0	0	0	6	0	1	8	18	4	22	6	7	27
PTEN	2	1	0	0	5	0	0	8	2	6	8	6	7	12
CACNA1A	0	0	0	0	6	1	0	7	4	3	7	6	7	13
CELSR1	2	0	0	0	4	0	1	7	0	16	16	6	7	22
LRRK2	2	1	0	0	3	1	0	7	8	1	9	6	7	13
MYO10	1	1	1	0	4	0	0	7	11	2	13	6	7	17
MYO15A	0	0	0	0	7	0	0	7	3	28	31	6	7	36
PTPRS	2	0	0	0	4	0	1	7	3	6	9	6	7	15
RIMBP2	1	0	0	0	6	0	0	7	8	1	9	6	7	13
RP1	1	0	0	0	6	0	0	7	11	8	19	6	7	23
SDK2	0	0	0	0	7	0	0	7	4	5	9	6	7	15
SPEG	2	0	0	0	4	0	1	7	3	2	5	6	7	11
VPS13B	1	1	0	0	5	0	0	7	24	2	26	6	7	31
ADGRB3	0	1	0	0	5	0	0	6	4	2	6	6	7	12
ADGRV1	0	2	0	0	3	0	1	6	5	8	13	6	7	19
AFF2	0	0	0	0	6	0	0	6	12	33	45	6	7	48
BIRC6	1	1	0	0	4	0	0	6	6	3	9	6	7	15
CTNNB1	0	0	1	0	4	0	1	6	3	1	4	6	7	10
CUBN	0	0	0	0	6	0	0	6	2	0	2	6	7	8
DNAH9	0	1	0	0	5	0	0	6	3	30	33	6	7	39
EP400	2	0	0	0	3	0	1	6	8	1	9	6	7	13
FAT3	0	0	0	0	6	0	0	6	2	4	6	6	7	12
FLT4	0	1	0	0	4	1	0	6	2	3	5	6	7	11
FNDC1	0	0	0	0	6	0	0	6	5	1	6	6	7	11
MAP1B	3	0	0	0	3	0	0	6	4	9	13	6	7	18
MYH6	0	0	0	0	6	0	0	6	2	6	8	6	7	14

NAV2	0	0	0	0	6	0	0	6	3	3	6	6	7	12
NLRP12	1	0	0	0	4	0	1	6	7	6	13	6	7	17
PCDH10	0	0	0	0	5	1	0	6	1	10	11	6	7	17
PEG3	1	0	0	0	5	0	0	6	0	0	0	6	7	6
RGS12	1	0	0	0	5	0	0	6	2	12	14	6	7	19
RIMS1	0	0	0	0	5	1	0	6	4	2	6	6	7	12
SALL1	0	0	0	0	6	0	0	6	12	0	12	6	7	17
STAB1	1	2	0	0	3	0	0	6	2	1	3	6	7	8
TRPM3	2	0	0	0	3	0	1	6	9	2	11	6	7	15
UNC13C	1	0	1	0	4	0	0	6	0	15	15	6	7	19
UNC80	0	0	0	0	6	0	0	6	3	3	6	6	7	12
ZNF462	0	1	0	0	5	0	0	6	7	3	10	6	7	15
PKD1	2	0	0	0	6	0	0	8	9	0	9	5	6	14
TNRC6B	4	0	0	0	3	1	0	8	0	14	14	5	6	19
UBR4	1	1	0	0	4	1	1	8	0	12	12	5	6	17
DSCAM	0	2	0	0	5	0	0	7	1	11	12	5	6	17
ACAN	1	1	0	0	4	0	0	6	1	14	15	5	6	20
ADAMTSL3	0	0	0	0	6	0	0	6	1	14	15	5	6	19
BCL9	1	3	0	0	1	1	0	6	16	0	16	5	6	20
CACNA1H	0	1	0	0	5	0	0	6	7	2	9	5	6	13
CELSR2	0	0	0	0	4	1	1	6	0	2	2	5	6	7
HTT	1	0	0	0	5	0	0	6	2	12	14	5	6	19
MAP2	0	0	0	0	6	0	0	6	4	3	7	5	6	12
TRIOBP	1	0	0	0	3	2	0	6	0	13	13	5	6	18
UBR5	4	0	0	0	2	0	0	6	24	2	26	5	6	31
ANKRD30A	1	0	0	0	4	0	0	5	11	0	11	5	6	16
BRCA2	0	1	0	0	3	0	1	5	31	0	31	5	6	34
CMYA5	1	0	0	0	4	0	0	5	4	9	13	5	6	18
CNTNAP5	0	0	0	0	5	0	0	5	4	2	6	5	6	11
COL5A1	0	1	0	0	4	0	0	5	7	4	11	5	6	15
COL6A3	0	0	0	0	5	0	0	5	4	1	5	5	6	9
CRMP1	0	0	0	0	5	0	0	5	2	12	14	5	6	19
DOCK4	0	0	0	0	5	0	0	5	22	2	24	5	6	28
DYNC1H1	0	0	0	0	5	0	0	5	3	7	10	5	6	15
FBN2	0	0	0	0	5	0	0	5	5	5	10	5	6	15
FBN3	0	0	0	0	5	0	0	5	3	13	16	5	6	21
FLNC	2	0	0	0	3	0	0	5	23	2	25	5	6	29
FRY	1	0	0	0	4	0	0	5	30	0	30	5	6	33
HECW1	0	1	0	0	3	1	0	5	27	0	27	5	6	28

LARP4B	5	0	0	0	0	0	0	5	2	0	2	5	6	7
LIFR	0	0	0	1	3	1	0	5	11	1	12	5	6	16
LRP2	1	0	0	0	2	0	2	5	4	0	4	5	6	8
MED12L	1	0	0	0	4	0	0	5	4	0	4	5	6	9
MINAR1	3	0	0	0	2	0	0	5	1	15	16	5	6	21
MYO16	1	1	0	0	3	0	0	5	29	1	30	5	6	33
NBEA	0	1	0	0	4	0	0	5	31	0	31	5	6	34
NCOR1	1	1	0	0	3	0	0	5	3	29	32	5	6	37
NIPBL	3	0	0	0	2	0	0	5	11	2	13	5	6	17
PCDHA2	1	0	0	0	4	0	0	5	0	0	0	5	6	5
PKHD1L1	1	0	0	0	4	0	0	5	24	2	26	5	6	31
PRRC2B	1	1	0	0	2	1	0	5	8	3	11	5	6	16
PTCH1	1	1	0	0	3	0	0	5	8	3	11	5	6	16
RELN	0	0	0	0	5	0	0	5	22	2	24	5	6	27
SCN5A	0	1	0	0	4	0	0	5	3	0	3	5	6	8
SRCAP	1	0	0	0	4	0	0	5	12	1	13	5	6	17
TNR	1	1	0	0	3	0	0	5	9	0	9	5	6	14
TRPS1	1	0	0	0	3	1	0	5	24	2	26	5	6	30
UTRN	1	0	0	0	4	0	0	5	3	1	4	5	6	9
WDFY4	0	0	0	0	5	0	0	5	2	1	3	5	6	8
DNAH14	2	1	0	0	5	1	0	9	14	0	14	4	5	17
RNF213	0	1	0	0	7	0	0	8	4	5	9	4	5	13
ABCA2	2	0	0	0	4	1	0	7	6	5	11	4	5	14
ALK	0	0	0	0	5	1	0	6	6	3	9	4	5	13
MYO18B	2	1	0	0	3	0	0	6	0	12	12	4	5	15
SETX	0	1	0	0	5	0	0	6	7	4	11	4	5	15
TG	1	0	0	0	5	0	0	6	25	3	28	4	5	30
ARHGEF17	0	0	0	0	5	0	0	5	1	3	4	4	5	8
BCORL1	2	0	0	0	3	0	0	5	12	32	44	4	5	45
BMPR2	2	0	0	0	3	0	0	5	5	3	8	4	5	12
BOD1L1	1	0	0	0	4	0	0	5	2	12	14	4	5	17
CDH10	1	0	0	0	4	0	0	5	9	2	11	4	5	15
DOCK5	4	0	0	0	1	0	0	5	6	18	24	4	5	28
DSCAML1	1	0	0	0	3	1	0	5	2	3	5	4	5	9
FASN	0	1	0	0	4	0	0	5	4	5	9	4	5	13
HERC1	1	0	0	0	1	2	1	5	0	17	17	4	5	21
HIVEP3	1	0	0	0	4	0	0	5	0	4	4	4	5	8
KIAA1549L	0	0	0	0	5	0	0	5	3	3	6	4	5	9
MDGA2	1	0	0	0	3	1	0	5	3	6	9	4	5	13

MGA	1	1	1	0	1	1	0	5	14	8	22	4	5	25
MYT1L	0	0	0	0	4	1	0	5	6	2	8	4	5	11
NCOR2	0	0	0	0	5	0	0	5	7	3	10	4	5	13
OTOF	0	0	0	0	5	0	0	5	6	3	9	4	5	13
PCDHB7	0	0	0	0	4	1	0	5	4	4	8	4	5	12
PXDNL	0	0	0	0	5	0	0	5	18	1	19	4	5	21
RALGAPA2	1	2	0	0	1	1	0	5	27	9	36	4	5	39
RNF43	5	0	0	0	0	0	0	5	5	4	9	4	5	13
TRIO	1	1	0	0	3	0	0	5	11	2	13	4	5	16
VPS13A	2	0	0	0	3	0	0	5	10	3	13	4	5	17
ZC3H13	1	1	0	0	3	0	0	5	29	2	31	4	5	34
ZNF407	1	0	0	0	4	0	0	5	2	33	35	4	5	38
ABCA1	0	0	0	0	4	0	0	4	11	0	11	4	5	15
ABCA4	2	0	0	0	2	0	0	4	0	2	2	4	5	6
ABCA7	0	0	0	0	4	0	0	4	3	6	9	4	5	12
ADAMTS12	0	0	0	0	4	0	0	4	11	2	13	4	5	17
ADAMTS9	0	0	0	0	4	0	0	4	3	1	4	4	5	8
AKAP13	0	0	0	0	4	0	0	4	1	14	15	4	5	18
APOB	0	0	0	0	4	0	0	4	6	2	8	4	5	12
ATP10A	0	0	0	0	4	0	0	4	0	20	20	4	5	24
BRAF	0	0	0	0	4	0	0	4	23	2	25	4	5	29
CDH9	1	0	0	0	3	0	0	4	10	1	11	4	5	15
DCC	0	0	0	0	4	0	0	4	2	33	35	4	5	37
DCLK1	0	0	0	0	4	0	0	4	30	0	30	4	5	32
ERICH3	0	0	0	0	4	0	0	4	0	2	2	4	5	6
FRMD4A	3	0	0	0	1	0	0	4	2	0	2	4	5	6
GRM1	1	1	0	0	2	0	0	4	3	1	4	4	5	8
HDAC9	0	0	0	0	3	1	0	4	28	1	29	4	5	32
HRNR	0	0	0	0	4	0	0	4	41	0	41	4	5	44
HUWE1	0	0	0	0	3	1	0	4	13	33	46	4	5	49
ITSN1	1	0	0	0	3	0	0	4	4	7	11	4	5	15
KMT2C	2	0	0	0	2	0	0	4	26	3	29	4	5	32
KNDC1	0	0	0	0	3	1	0	4	2	3	5	4	5	7
LTBP4	0	0	0	0	2	2	0	4	7	6	13	4	5	17
MAGEC1	0	0	0	0	3	1	0	4	12	32	44	4	5	47
MAGI1	0	0	0	0	4	0	0	4	3	1	4	4	5	7
MYCBP2	0	0	0	0	4	0	0	4	30	2	32	4	5	36
MYH13	0	0	0	0	4	0	0	4	3	30	33	4	5	37
MYH7	1	0	0	0	3	0	0	4	2	6	8	4	5	12

NOTCH1	0	0	0	0	4	0	0	4	6	4	10	4	5	14
NPAP1	0	0	0	0	4	0	0	4	0	19	19	4	5	23
NRAS	0	0	0	0	4	0	0	4	1	1	2	4	5	6
NRXN3	0	0	0	0	4	0	0	4	3	5	8	4	5	12
OTOGL	1	1	0	0	2	0	0	4	9	2	11	4	5	13
PAPPA	1	0	0	0	3	0	0	4	7	3	10	4	5	12
PCDH15	0	0	0	0	4	0	0	4	2	1	3	4	5	7
PCDH19	0	0	0	0	4	0	0	4	11	34	45	4	5	47
PCDHA13	0	0	0	0	4	0	0	4	0	0	0	4	5	4
PCDHGA1	0	0	0	0	4	0	0	4	4	4	8	4	5	12
PCDHGB1	0	0	0	0	4	0	0	4	0	0	0	4	5	4
PCDHGB4	0	0	0	0	4	0	0	4	0	0	0	4	5	4
PDZD2	1	0	1	0	2	0	0	4	10	1	11	4	5	15
PKD1L1	0	3	0	0	1	0	0	4	27	0	27	4	5	31
PLXNA3	2	0	0	0	2	0	0	4	13	33	46	4	5	48
RAI1	0	0	0	0	4	0	0	4	3	29	32	4	5	36
ROBO1	0	0	0	0	4	0	0	4	3	0	3	4	5	7
RREB1	0	0	0	0	4	0	0	4	9	2	11	4	5	14
SCN1A	1	0	0	0	3	0	0	4	11	1	12	4	5	15
SCN3A	0	0	0	0	3	1	0	4	11	0	11	4	5	14
SLIT1	0	0	0	0	4	0	0	4	1	6	7	4	5	11
SLIT2	0	0	0	0	4	0	0	4	2	12	14	4	5	17
SLITRK1	0	0	0	0	4	0	0	4	29	2	31	4	5	33
SPTA1	0	0	0	0	3	1	0	4	11	0	11	4	5	15
STAB2	0	0	0	0	4	0	0	4	7	3	10	4	5	13
TENM1	1	0	0	0	3	0	0	4	12	32	44	4	5	46
TIAM1	1	0	0	0	3	0	0	4	4	7	11	4	5	15
TRPM2	0	0	0	0	3	1	0	4	1	12	13	4	5	17
WNK2	0	0	0	0	4	0	0	4	8	2	10	4	5	13
LRP1	1	0	0	0	6	0	0	7	8	0	8	3	4	10
ASPM	0	0	0	0	6	0	0	6	13	0	13	3	4	15
FANCM	2	0	0	0	4	0	0	6	3	6	9	3	4	12
PLEKHA6	4	0	0	0	2	0	0	6	13	0	13	3	4	15
AFDN	0	1	0	0	4	0	0	5	5	2	7	3	4	10
ASXL3	0	0	0	0	5	0	0	5	2	29	31	3	4	34
HELZ2	0	0	0	0	5	0	0	5	39	5	44	3	4	46
NCAN	1	0	0	0	4	0	0	5	5	4	9	3	4	12
SZT2	2	0	0	0	3	0	0	5	0	3	3	3	4	6
TNN	0	0	0	0	5	0	0	5	9	0	9	3	4	12

A2ML1	0	0	0	0	4	0	0	4	8	2	10	3	4	13
ANK1	0	0	0	0	4	0	0	4	13	5	18	3	4	21
ATP2B3	1	0	0	0	3	0	0	4	12	33	45	3	4	47
CEP350	2	0	0	0	2	0	0	4	10	0	10	3	4	12
DNAH3	0	0	0	0	3	1	0	4	9	1	10	3	4	13
DYSF	2	0	0	0	2	0	0	4	6	2	8	3	4	11
FAM135B	0	1	0	0	2	1	0	4	25	3	28	3	4	30
FHOD3	1	0	0	0	3	0	0	4	2	31	33	3	4	36
IGF2R	1	1	0	0	2	0	0	4	5	1	6	3	4	9
NFASC	1	0	0	0	3	0	0	4	13	0	13	3	4	16
NUP205	0	1	0	0	2	0	1	4	23	2	25	3	4	28
NYNRIN	0	1	0	0	3	0	0	4	2	6	8	3	4	11
PCDHB3	0	0	0	0	4	0	0	4	4	4	8	3	4	11
SCN9A	0	1	0	0	3	0	0	4	11	0	11	3	4	14
SDK1	0	0	0	0	4	0	0	4	28	0	28	3	4	30
UTP20	1	0	0	0	3	0	0	4	7	2	9	3	4	11
VWF	1	1	0	0	2	0	0	4	8	2	10	3	4	12
XYLT2	2	1	0	0	0	1	0	4	4	4	8	3	4	11
ABCA3	0	0	0	0	3	0	0	3	7	3	10	3	4	12
ABCA6	3	0	0	0	0	0	0	3	4	4	8	3	4	11
ADAMTS20	0	0	0	0	3	0	0	3	8	0	8	3	4	11
ADCY8	0	0	0	0	2	1	0	3	25	3	28	3	4	31
ARID2	0	0	0	0	2	1	0	3	8	0	8	3	4	11
ASXL1	3	0	0	0	0	0	0	3	42	5	47	3	4	48
BCOR	0	1	1	0	1	0	0	3	11	32	43	3	4	44
BSN	0	0	0	0	3	0	0	3	2	1	3	3	4	5
CACNA1G	0	0	0	0	2	0	1	3	4	4	8	3	4	11
CEP250	0	0	0	0	2	0	1	3	42	6	48	3	4	49
CHD3	1	0	1	0	1	0	0	3	3	30	33	3	4	36
CHD6	0	0	0	0	2	1	0	3	43	5	48	3	4	49
CNTNAP4	1	0	0	0	2	0	0	3	10	2	12	3	4	15
COL22A1	1	0	0	0	1	0	1	3	25	3	28	3	4	31
COL6A6	0	0	0	0	3	0	0	3	4	1	5	3	4	8
CPAMD8	0	0	0	0	3	0	0	3	4	4	8	3	4	11
CPLANE1	2	0	0	0	1	0	0	3	11	2	13	3	4	16
CSPG4	0	0	0	0	2	1	0	3	0	18	18	3	4	21
DIP2C	0	0	0	0	3	0	0	3	2	0	2	3	4	5
DLEC1	0	1	0	0	2	0	0	3	3	0	3	3	4	6
DNAH10	0	0	0	0	3	0	0	3	7	3	10	3	4	12

DNAH8	1	0	0	0	1	1	0	3	8	0	8	3	4	11
DSP	0	0	0	0	3	0	0	3	9	2	11	3	4	14
EPHB1	0	0	0	0	3	0	0	3	4	0	4	3	4	6
EYS	0	0	0	0	2	1	0	3	8	1	9	3	4	11
FRAS1	2	0	0	0	1	0	0	3	2	9	11	3	4	14
GOLGB1	0	0	0	0	2	1	0	3	3	0	3	3	4	6
HERC2	0	0	0	0	3	0	0	3	0	33	33	3	4	34
ICE1	1	0	0	0	2	0	0	3	11	3	14	3	4	17
ITGB4	0	1	0	0	2	0	0	3	4	5	9	3	4	11
ITPR1	0	0	0	0	3	0	0	3	3	0	3	3	4	6
JCAD	1	0	0	0	2	0	0	3	3	0	3	3	4	6
KALRN	1	0	0	0	2	0	0	3	3	0	3	3	4	6
KIAA1549	1	0	0	0	2	0	0	3	23	2	25	3	4	27
KIF2B	0	0	0	0	3	0	0	3	4	4	8	3	4	11
LAMA3	0	0	0	0	3	0	0	3	3	24	27	3	4	30
LAMA4	1	0	0	0	2	0	0	3	3	3	6	3	4	9
LRRC7	0	0	0	0	3	0	0	3	0	4	4	3	4	7
LYST	1	0	1	0	1	0	0	3	12	0	12	3	4	15
MGAM	0	0	0	0	3	0	0	3	36	5	41	3	4	42
NALCN	0	0	0	0	3	0	0	3	29	2	31	3	4	33
NID1	0	0	0	0	3	0	0	3	11	0	11	3	4	13
NIN	0	0	0	0	3	0	0	3	2	6	8	3	4	11
PCDHGA2	0	1	0	0	2	0	0	3	0	0	0	3	4	3
PIK3CG	0	0	0	0	3	0	0	3	22	2	24	3	4	27
PIK3R1	0	0	2	0	0	0	1	3	3	11	14	3	4	17
PKDREJ	1	0	0	0	2	0	0	3	0	16	16	3	4	19
PLCB1	1	0	0	0	2	0	0	3	24	10	34	3	4	35
PLXNB2	0	0	0	0	3	0	0	3	0	14	14	3	4	17
PRUNE2	0	0	0	0	3	0	0	3	9	3	12	3	4	15
REV3L	0	2	0	0	1	0	0	3	3	2	5	3	4	8
RGS22	0	1	0	0	2	0	0	3	24	2	26	3	4	28
SCN11A	0	1	0	0	2	0	0	3	3	0	3	3	4	6
SCN2A	0	0	0	0	3	0	0	3	11	1	12	3	4	15
SEC16A	0	0	0	0	3	0	0	3	6	4	10	3	4	12
SLITRK5	0	0	0	0	3	0	0	3	29	2	31	3	4	33
SPTBN1	1	0	0	0	2	0	0	3	6	2	8	3	4	11
SPTBN2	0	0	0	0	3	0	0	3	2	3	5	3	4	8
SVEP1	0	0	0	0	3	0	0	3	7	3	10	3	4	12
TCHH	0	0	1	0	2	0	0	3	31	0	31	3	4	33

TECTA	0	0	0	0	3	0	0	3	2	3	5	3	4	8
TENM2	0	0	0	0	3	0	0	3	3	4	7	3	4	10
TEX15	2	0	0	0	1	0	0	3	9	16	25	3	4	28
TRPC4	0	0	0	0	3	0	0	3	30	1	31	3	4	32
UNC13A	0	0	0	0	3	0	0	3	5	4	9	3	4	12
USP34	0	0	0	0	3	0	0	3	7	1	8	3	4	11
YLPM1	1	1	0	0	0	1	0	3	2	7	9	3	4	12
ZDBF2	1	0	0	0	2	0	0	3	4	3	7	3	4	10
ZNF423	0	0	0	0	3	0	0	3	12	1	13	3	4	15
ACACB	1	0	0	0	4	0	0	5	7	2	9	2	2	10
SVIL	1	0	0	0	4	0	0	5	3	0	3	2	2	5
CDC42BPA	0	0	0	0	4	0	0	4	13	1	14	2	2	15
LAMA2	0	0	0	0	3	1	0	4	3	3	6	2	2	8
CNKSR3	0	0	0	0	3	0	0	3	3	1	4	2	2	6
EGFR	0	1	0	0	2	0	0	3	28	0	28	2	2	30
EVPL	0	0	0	0	3	0	0	3	4	5	9	2	2	11
GREB1	0	0	0	0	3	0	0	3	6	2	8	2	2	10
KDM2B	2	1	0	0	0	0	0	3	7	0	7	2	2	8
LRRIQ1	0	0	0	0	2	1	0	3	11	2	13	2	2	14
MYH8	0	0	0	0	2	0	1	3	3	30	33	2	2	34
MYO7B	0	0	1	0	2	0	0	3	4	4	8	2	2	10
SCN7A	0	0	0	0	3	0	0	3	7	0	7	2	2	9
TPR	1	0	0	0	2	0	0	3	9	2	11	2	2	12
ZNF106	1	0	0	0	2	0	0	3	0	21	21	2	2	23
ABCC8	0	0	0	0	2	0	0	2	3	3	6	2	2	8
ADAMTS18	1	0	0	0	1	0	0	2	11	2	13	2	2	15
ADGRG4	0	0	0	0	2	0	0	2	12	32	44	2	2	45
ADGRL2	0	0	0	0	2	0	0	2	0	2	2	2	2	4
ADGRL3	0	0	0	0	2	0	0	2	2	10	12	2	2	14
AKAP12	1	0	0	0	1	0	0	2	3	1	4	2	2	6
ANKRD11	1	0	0	0	1	0	0	2	10	2	12	2	2	14
APBA1	0	0	0	0	2	0	0	2	9	2	11	2	2	13
ASTN2	0	0	0	0	2	0	0	2	7	4	11	2	2	13
ATP8A2	0	0	0	0	1	0	1	2	31	0	31	2	2	31
C3	0	0	0	0	2	0	0	2	7	3	10	2	2	12
CAMSAP1	0	0	0	0	1	1	0	2	6	3	9	2	2	11
CARD11	0	0	0	0	2	0	0	2	28	0	28	2	2	30
CDH18	1	0	0	0	1	0	0	2	10	2	12	2	2	14
CDH2	0	0	0	0	1	1	0	2	2	27	29	2	2	31

CDH8	0	0	0	0	2	0	0	2	16	0	16	2	2	18
CHD7	0	0	0	0	2	0	0	2	19	3	22	2	2	24
CNTN6	0	0	0	0	2	0	0	2	3	0	3	2	2	4
CNTRL	0	0	0	0	2	0	0	2	7	4	11	2	2	13
COL27A1	1	0	0	0	1	0	0	2	7	3	10	2	2	12
COL5A2	0	0	0	0	1	1	0	2	6	2	8	2	2	9
CR1	0	0	0	0	1	1	0	2	22	0	22	2	2	24
CUL9	0	0	0	0	2	0	0	2	9	0	9	2	2	11
DAPK1	1	0	0	0	1	0	0	2	8	2	10	2	2	12
DLC1	1	0	0	0	1	0	0	2	7	17	24	2	2	26
DNAH2	1	0	0	0	1	0	0	2	3	29	32	2	2	33
ERBB4	0	0	0	0	1	1	0	2	3	2	5	2	2	7
FMN2	0	0	0	0	2	0	0	2	10	0	10	2	2	12
GRIN2A	0	0	0	0	2	0	0	2	8	6	14	2	2	16
HIVEP2	0	1	0	0	1	0	0	2	3	1	4	2	2	6
INF2	0	0	0	0	2	0	0	2	6	7	13	2	2	14
LRIF1	0	0	0	0	1	1	0	2	1	1	2	2	2	4
MAGI2	0	0	0	0	2	0	0	2	26	2	28	2	2	29
MAP1A	0	0	0	0	2	0	0	2	0	20	20	2	2	22
MED12	0	0	0	0	2	0	0	2	13	33	46	2	2	47
MEGF6	0	1	0	0	1	0	0	2	1	29	30	2	2	32
MKI67	1	0	0	0	1	0	0	2	3	3	6	2	2	8
MN1	0	0	0	0	2	0	0	2	3	12	15	2	2	17
MSH6	1	0	1	0	0	0	0	2	6	4	10	2	2	12
MTOR	0	0	0	0	2	0	0	2	0	6	6	2	2	8
NANOS1	0	0	0	0	2	0	0	2	1	0	1	2	2	3
NBEAL2	0	0	0	0	2	0	0	2	2	2	4	2	2	5
NF1	0	0	0	0	2	0	0	2	8	5	13	2	2	14
NHS	0	0	0	0	2	0	0	2	11	33	44	2	2	45
NLRP4	0	0	0	0	2	0	0	2	7	5	12	2	2	14
NOTCH3	0	0	0	0	2	0	0	2	4	5	9	2	2	11
PCDHA1	0	0	0	0	2	0	0	2	4	6	10	2	2	12
PCDHA6	0	0	0	0	2	0	0	2	0	0	0	2	2	2
PCDHAC1	0	0	0	0	2	0	0	2	0	0	0	2	2	2
PLXNA1	1	0	0	0	1	0	0	2	3	0	3	2	2	5
PLXNA2	0	1	0	0	1	0	0	2	12	0	12	2	2	14
PLXNA4	0	0	0	0	1	1	0	2	23	2	25	2	2	27
POLE	0	0	0	0	2	0	0	2	7	2	9	2	2	9
PRDM2	2	0	0	0	0	0	0	2	0	7	7	2	2	9

PTPRT	0	0	0	0	2	0	0	2	43	5	48	2	2	48
PTPRU	1	0	0	0	1	0	0	2	0	10	10	2	2	12
RASGRF1	0	0	0	0	2	0	0	2	0	17	17	2	2	18
ROBO3	1	0	0	0	1	0	0	2	3	3	6	2	2	8
SBF1	0	1	0	0	1	0	0	2	0	14	14	2	2	16
SCN10A	0	0	0	0	2	0	0	2	3	0	3	2	2	5
SCN4A	0	0	0	0	2	0	0	2	4	4	8	2	2	10
SETBP1	1	0	0	0	1	0	0	2	2	31	33	2	2	35
SLIT3	0	0	0	0	2	0	0	2	2	4	6	2	2	8
SMARCA4	0	0	0	0	2	0	0	2	4	2	6	2	2	8
SNRNP200	0	0	0	0	1	1	0	2	5	0	5	2	2	7
SPATA31D1	0	0	0	0	2	0	0	2	8	3	11	2	2	13
SPECC1	2	0	0	0	0	0	0	2	2	26	28	2	2	30
SRRM2	0	0	0	0	2	0	0	2	7	3	10	2	2	11
TAF1L	1	0	0	0	1	0	0	2	10	3	13	2	2	14
TNC	0	0	0	0	1	1	0	2	7	3	10	2	2	12
TSC2	0	0	1	0	1	0	0	2	7	2	9	2	2	11
USP35	2	0	0	0	0	0	0	2	1	3	4	2	2	6
WDFY3	0	0	0	0	2	0	0	2	1	10	11	2	2	13
WNK1	0	0	0	0	2	0	0	2	11	2	13	2	2	14
ZIM2	0	0	0	0	2	0	0	2	7	4	11	2	2	13
ZNF142	0	0	0	0	2	0	0	2	3	1	4	2	2	6
FLNB	2	0	0	0	0	0	0	2	3	1	4	1	1	5
FREM1	0	0	0	0	2	0	0	2	10	4	14	1	1	15
HCN1	0	1	0	0	1	0	0	2	11	1	12	1	1	13
JARID2	0	0	0	0	2	0	0	2	8	2	10	1	1	10
LCT	0	0	0	0	2	0	0	2	3	2	5	1	1	6
TEP1	0	0	0	0	2	0	0	2	2	6	8	1	1	9
TSHZ3	0	1	0	0	1	0	0	2	8	2	10	1	1	11
ABCC5	0	0	0	0	1	0	0	1	5	1	6	1	1	7
ACOD1	0	0	0	0	1	0	0	1	30	1	31	1	1	31
B4GALNT2	0	0	0	0	0	1	0	1	4	4	8	1	1	9
BRD4	0	0	0	0	1	0	0	1	4	5	9	1	1	10
CAD	0	0	0	0	1	0	0	1	6	3	9	1	1	10
CHD5	0	0	0	0	1	0	0	1	1	9	10	1	1	11
CNOT1	0	0	0	0	1	0	0	1	11	0	11	1	1	12
COL4A2	0	0	0	0	1	0	0	1	29	1	30	1	1	31
DMBT1	0	0	0	0	1	0	0	1	52	4	56	1	1	57
DPYD	0	0	0	0	1	0	0	1	0	1	1	1	1	2

DYNLL1	0	0	0	0	1	0	0	1	7	0	7	1	1	8
EPHA3	0	0	0	0	1	0	0	1	3	0	3	1	1	4
ESRP1	1	0	0	0	0	0	0	1	24	3	27	1	1	28
F8	0	0	0	0	1	0	0	1	22	23	45	1	1	45
FAT1	0	0	0	0	1	0	0	1	1	11	12	1	1	13
FER1L6	1	0	0	0	0	0	0	1	25	3	28	1	1	29
FGFR1	0	0	0	0	1	0	0	1	14	10	24	1	1	25
GCN1	0	0	0	0	1	0	0	1	7	0	7	1	1	8
HCFC1	0	0	0	0	1	0	0	1	13	30	43	1	1	43
IGSF10	0	0	0	0	1	0	0	1	4	0	4	1	1	5
KATNIP	0	0	0	0	1	0	0	1	11	1	12	1	1	12
KCNMA1	1	0	0	0	0	0	0	1	2	4	6	1	1	7
KDM3B	1	0	0	0	0	0	0	1	4	5	9	1	1	10
KDR	0	1	0	0	0	0	0	1	2	9	11	1	1	12
KNL1	0	0	0	0	1	0	0	1	0	22	22	1	1	23
LAMB2	0	0	0	0	1	0	0	1	2	1	3	1	1	4
LAMC1	0	0	0	0	1	0	0	1	11	0	11	1	1	12
NCKAP5	0	0	0	0	1	0	0	1	4	2	6	1	1	7
NLRP5	0	0	0	0	1	0	0	1	7	5	12	1	1	13
NWD1	0	0	0	0	1	0	0	1	4	4	8	1	1	9
PCDHA11	0	0	0	0	1	0	0	1	0	0	0	1	1	1
PCDHA3	1	0	0	0	0	0	0	1	0	0	0	1	1	1
PCDHGA5	0	0	0	0	1	0	0	1	0	0	0	1	1	1
PDE4DIP	0	1	0	0	0	0	0	1	16	0	16	1	1	16
PLCG2	0	0	0	0	1	0	0	1	10	2	12	1	1	13
PLEKHG4B	0	0	0	0	1	0	0	1	12	1	13	1	1	14
PLXNB3	0	0	0	0	1	0	0	1	13	33	46	1	1	47
POLQ	0	0	0	0	1	0	0	1	3	0	3	1	1	4
PTPRZ1	0	0	0	0	1	0	0	1	22	2	24	1	1	25
RALGAPA1	0	0	0	0	0	1	0	1	3	6	9	1	1	9
RIMS2	0	0	0	0	1	0	0	1	24	2	26	1	1	26
SCLT1	0	1	0	0	0	0	0	1	1	10	11	1	1	12
SIN3B	0	0	0	0	1	0	0	1	4	4	8	1	1	9
SLC6A4	0	0	0	0	1	0	0	1	11	9	20	1	1	21
SPEGNB	0	0	0	0	1	0	0	1	3	1	4	1	1	5
SPHKAP	0	0	0	0	1	0	0	1	3	1	4	1	1	5
SPTB	0	0	0	0	1	0	0	1	2	8	10	1	1	11
TAOK2	0	0	0	0	1	0	0	1	12	1	13	1	1	14
TLN2	0	0	0	0	0	0	1	1	0	18	18	1	1	19

TMEM131	0	0	0	0	1	0	0	1	6	0	6	1	1	7
TNRC6A	0	0	1	0	0	0	0	1	11	1	12	1	1	13
ZNF804A	0	0	0	0	1	0	0	1	6	1	7	1	1	8
ABCC1	0	0	0	0	0	0	0	0	8	12	20	0	0	20
AHNAK	0	0	0	0	0	0	0	0	10	3	13	0	0	13
AHNAK2	0	0	0	0	0	0	0	0	32	6	38	0	0	38
ATRX	0	0	0	0	0	0	0	0	10	35	45	0	0	45
BRINP3	0	0	0	0	0	0	0	0	11	0	11	0	0	11
C1D	0	0	0	0	0	0	0	0	6	3	9	0	0	9
CD36	0	0	0	0	0	0	0	0	26	2	28	0	0	28
CHD9	0	0	0	0	0	0	0	0	11	0	11	0	0	11
DFFB	0	0	0	0	0	0	0	0	1	25	26	0	0	26
DLGAP2	0	0	0	0	0	0	0	0	8	20	28	0	0	28
DMXL2	0	0	0	0	0	0	0	0	0	15	15	0	0	15
DNAH11	0	0	0	0	0	0	0	0	28	0	28	0	0	28
DNAH17	0	0	0	0	0	0	0	0	4	5	9	0	0	9
DOCK8	0	0	0	0	0	0	0	0	11	4	15	0	0	15
DSEL	0	0	0	0	0	0	0	0	2	33	35	0	0	35
DST	0	0	0	0	0	0	0	0	8	0	8	0	0	8
FLG	0	0	0	0	0	0	0	0	52	0	52	0	0	52
GET1	0	0	0	0	0	0	0	0	1	8	9	0	0	9
HMCN1	0	0	0	0	0	0	0	0	11	0	11	0	0	11
HSPG2	0	0	0	0	0	0	0	0	0	21	21	0	0	21
INSL6	0	0	0	0	0	0	0	0	11	3	14	0	0	14
KIF26B	0	0	0	0	0	0	0	0	10	7	17	0	0	17
LAMA5	0	0	0	0	0	0	0	0	39	5	44	0	0	44
MACF1	0	0	0	0	0	0	0	0	0	3	3	0	0	3
MAPRE2	0	0	0	0	0	0	0	0	2	29	31	0	0	31
METAP1	0	0	0	0	0	0	0	0	1	10	11	0	0	11
METAP2	0	0	0	0	0	0	0	0	8	2	10	0	0	10
MUC16	0	0	0	0	0	0	0	0	12	11	23	0	0	23
MUC17	0	0	0	0	0	0	0	0	30	3	33	0	0	33
MUC5AC	0	0	0	0	0	0	0	0	3	4	7	0	0	7
MUC5B	0	0	0	0	0	0	0	0	4	4	8	0	0	8
MUC6	0	0	0	0	0	0	0	0	3	4	7	0	0	7
MYH11	0	0	0	0	0	0	0	0	8	12	20	0	0	20
NEB	0	0	0	0	0	0	0	0	36	2	38	0	0	38
NOTCH2	0	0	0	0	0	0	0	0	2	1	3	0	0	3
OBSCN	0	0	0	0	0	0	0	0	18	2	20	0	0	20

PHRF1	0	0	0	0	0	0	0	0	3	4	7	0	0	7
PLEC	0	0	0	0	0	0	0	0	25	8	33	0	0	33
POLK	0	0	0	0	0	0	0	0	4	9	13	0	0	13
PTPRK	0	0	0	0	0	0	0	0	3	4	7	0	0	7
RIF1	0	0	0	0	0	0	0	0	4	2	6	0	0	6
RNASE12	0	0	0	0	0	0	0	0	2	6	8	0	0	8
RP1L1	0	0	0	0	0	0	0	0	8	17	25	0	0	25
SLX4	0	0	0	0	0	0	0	0	7	3	10	0	0	10
STK19	0	0	0	0	0	0	0	0	1	1	2	0	0	2
SYNE1	0	0	0	0	0	0	0	0	3	2	5	0	0	5
SYNE2	0	0	0	0	0	0	0	0	2	7	9	0	0	9
TENT4A	0	0	0	0	0	0	0	0	11	2	13	0	0	13
TNXB	0	0	0	0	0	0	0	0	8	2	10	0	0	10
TTN	0	0	0	0	0	0	0	0	56	0	56	0	0	56
UNC79	0	0	0	0	0	0	0	0	3	7	10	0	0	10
USH2A	0	0	0	0	0	0	0	0	16	0	16	0	0	16
ZNF318	0	0	0	0	0	0	0	0	9	0	9	0	0	9

Supplementary Table S3: Summary of somatic mutational profile

**Tumor_Sample_ Barcode**	**Mutations with HIGH predicted effect on the protein**				**HIGH total**	**Mutations with MODERATE predicted effect on the protein**			**MODERATE total**	**All non-silent mutations**	**TMB**	**Large alterations**		**CNV characteristics**						**MSIsensor2**			**MSIsensor-pro (pairs tumor-blood)**			**jvarkit**		**MSI-high**
	Frame_Shift_ Del	Frame_Shift_I ns	Nonsense_ Mutation	Splice_S ite		In_Frame_ Del	In_Frame_I ns	Missense_ Mutation				Amplifications	Deletions	CNV_0	CNV_1	CNV_2	CNV_3	CNV_>3	Mean_length_ bp	Total_Number_ of_Sites	Number_of_Somatic_Si tes	%	Total_Number_of_S ites	Number_of_Somatic_S ites	%	No_of_indels_in_h omopolymers	indels_per_Mbp	
C3T	55	10	7	0	72	1	0	114	115	187	36.67	30	27	0	5	0	4	0	25731537	181	137	75.69	1324	512	38.67	237	46.47	yes
C4T	0	0	2	0	2	0	0	17	17	19	3.73	64	75	0	16	0	12	0	18771800	189	3	1.59	1611	112	6.95	2	0.39	no
C7T	0	0	0	0	0	0	0	0	0	0	0	12	23	0	6	0	3	1	8666730	190	3	1.58	1076	128	11.9	0	0.00	no
C9T	0	0	0	0	0	0	0	0	0	0	0	4	21	0	4	0	3	1	14254400	188	1	0.53	1604	124	7.73	0	0	no
C10T	0	0	0	0	0	0	0	0	0	0	0	4	20	0	3	0	3	1	16000800	188	4	2.13	1505	108	7.18	0	0.00	no
C13T	0	0	0	0	0	0	0	0	0	0	0	4	24	0	7	0	3	0	11172800	186	0	0	1726	77	4.46	1	0.20	no
C19T	0	0	0	0	0	0	0	0	0	0	0	3	20	0	8	0	3	1	20711700	179	0	0	1341	79	5.89	0	0.00	no
C15T	0	0	0	0	0	0	0	0	0	0	0	18	49	0	3	0	2	0	21319000	184	1	0.54	1169	79	6.76	0	0.00	no
C20T	0	0	0	0	0	0	0	0	0	0	0	3	0	0	0	0	2	0	53894	174	1	0.57	1341	52	3.88	1	0.20	no
C24T	0	0	0	0	0	0	0	0	0	0	0	19	30	0	7	0	5	1	12127800	180	3	1.67	1091	96	8.8	1	0.20	no
C29T	0	2	1	0	3	0	0	5	5	8	1.57	5	26	0	3	0	3	0	25550900	193	3	1.55	706	43	6.09	1	0.20	no
C33T	1	1	3	0	5	1	0	34	35	40	7.84	17	5	0	3	0	4	5	1685830	186	2	1.08	1054	52	4.93	5	0.98	no
C36T	1	1	3	0	5	0	0	17	17	22	4.31	56	27	0	8	0	14	3	11013100	185	0	0	924	54	5.84	4	0.78	no
C65T	1	1	0	0	2	0	0	18	18	20	3.92	77	38	0	7	0	13	2	20093700	189	3	1.59	567	33	5.82	1	0.20	no
C77T	1	1	1	0	3	1	0	11	12	15	2.94	84	17	0	2	0	9	0	36237200	179	3	1.68	777	19	2.45	0	0.00	no
C78T	0	0	2	0	2	1	0	11	12	14	2.75	57	72	1	10	0	4	4	22508100	174	7	4.02	1187	13	1.1	0	0.00	no
C80T	2	1	2	1	6	2	0	30	32	38	7.45	72	66	1	20	0	17	2	10707600	190	2	1.05	1678	133	7.93	6	1.18	no
C87T	0	0	0	0	0	0	0	9	9	9	1.76	21	14	0	9	0	11	3	1329730	191	1	0.52	1855	140	7.55	0	0.00	no
C100T	0	0	0	0	0	0	0	6	6	6	1.18	2	28	0	6	0	2	0	15865600	180	0	0	1122	110	9.8	1	0.20	no
C108T	0	0	3	1	4	0	0	11	11	15	2.94	47	47	0	9	0	11	2	16908200	188	3	1.6	1546	101	6.53	1	0.20	no
C111T	2	0	3	0	5	0	0	9	9	14	2.75	44	4	0	2	0	11	1	9528470	185	1	0.54	1402	90	6.42	1	0.20	no
C112T	0	0	0	0	0	0	0	0	0	0	0	8	25	0	8	0	8	1	7157820	189	1	0.53	1820	80	4.4	3	0.59	no
C117T	0	1	3	0	4	0	0	17	17	21	4.12	137	31	1	9	0	22	5	13763700	185	5	2.7	1479	94	6.36	3	0.59	no
C123T	0	0	1	2	3	0	0	7	7	10	1.96	51	67	0	15	0	10	0	14301200	185	2	1.08	1256	53	4.22	1	0.20	no
C143T	2	0	1	1	4	0	0	24	24	28	5.49	121	23	0	6	0	15	4	18872800	187	1	0.53	1208	96	7.95	5	0.98	no
C146T	3	0	0	0	3	0	0	16	16	19	3.73	71	50	0	16	0	7	8	14753800	179	3	1.68	1612	81	5.02	3	0.59	no
C150T	2	2	2	1	7	0	0	23	23	30	5.88	191	21	0	5	0	15	5	33050200	179	1	0.56	1761	51	2.9	4	0.78	no
C154T	2	1	2	0	5	0	0	17	17	22	4.31	61	55	0	11	0	10	3	11955700	190	2	1.05	1870	56	2.99	2	0.39	no
C155T	55	7	8	3	73	1	0	116	117	190	37.25	43	36	0	7	0	7	1	21370600	189	132	69.84	1904	738	38.76	261	51.18	yes
C158T	1	0	2	1	4	2	0	25	27	31	6.08	79	57	0	15	0	13	1	12788470	184	3	1.63	1292	67	5.19	4	0.78	no
C163T	2	0	1	0	3	0	0	13	13	16	3.14	123	97	0	15	0	18	1	27803300	189	4	2.12	1709	72	4.21	2	0.39	no
C170T	0	0	0	0	0	0	0	0	0	0	0	8	41	0	14	0	4	0	3872500	192	2	1.04	1570	88	5.61	0	0.00	no
C171T	0	2	1	1	4	0	0	28	28	32	6.27	125	89	0	12	0	14	5	24051900	189	5	2.65	1591	91	5.72	4	0.78	no
C177T	48	18	11	2	79	2	0	237	239	318	62.35	8	48	0	12	0	2	1	11098400	191	162	84.82	1706	711	41.68	383	75.10	yes
C178T	0	0	1	0	1	0	0	11	11	12	2.35	69	56	0	13	0	12	4	13444400	188	1	0.53	1612	126	7.82	6	1.18	no
C181T	0	1	2	0	3	0	0	17	17	20	3.92	50	48	1	12	0	10	0	13795100	192	0	0	1540	336	21.82	1	0.20	no
C183T	1	1	3	0	5	0	0	20	20	25	4.90	50	26	0	4	0	6	1	28041300	188	1	0.53	2002	77	3.85	9	1.76	no
C184T	2	0	2	0	4	0	0	15	15	19	3.73	47	63	1	13	0	7	1	19236100	191	1	0.52	1415	198	13.99	0	0.00	no
C188T	1	0	0	0	1	0	0	18	18	19	3.73	96	46	1	11	0	13	5	19122200	194	3	1.55	1850	157	8.49	2	0.39	no
C189T	0	2	0	0	2	1	0	8	9	11	2.16	5	8	0	3	0	3	2	1708780	193	1	0.52	1834	112	6.11	1	0.20	no
C191T	1	0	0	2	3	0	0	12	12	15	2.94	127	68	0	18	0	13	5	19084400	191	1	0.52	1894	157	8.29	1	0.20	no
C197T	1	0	0	2	3	0	0	15	15	18	3.53	190	94	0	15	0	17	1	32381200	185	1	0.54	1323	95	7.18	1	0.20	no
C198T	16	6	3	2	27	2	0	42	44	71	13.92	48	2	0	1	0	4	4	20635000	184	91	49.46	591	122	20.64	65	12.75	yes
C199T	0	0	1	0	1	0	0	13	13	14	2.75	112	165	0	23	0	13	3	26976000	189	1	0.53	789	54	6.84	3	0.59	no
C203T	1	1	3	0	5	0	0	16	16	21	4.12	52	56	0	10	0	11	1	14898955	190	1	0.53	728	56	7.69	5	0.98	no
C209T	0	0	3	0	3	0	0	21	21	24	4.71	138	0	0	0	0	14	2	32284800	190	2	1.05	872	66	7.57	3	0.59	no
C212T	0	0	0	0	0	1	0	11	12	12	2.35	112	41	0	6	0	12	3	28262500	192	1	0.52	760	59	7.76	1	0.20	no
C218T	0	0	1	0	1	0	0	16	16	17	3.33	14	45	0	7	0	6	1	12872300	192	3	1.56	804	85	10.57	2	0.39	no
C220T	1	2	3	0	6	0	0	13	13	19	3.73	42	37	0	10	0	8	2	12780800	193	1	0.52	926	80	8.64	3	0.59	no
C222T	0	0	3	0	3	0	0	11	11	14	2.75	210	70	0	13	0	25	9	24676700	183	4	2.19	822	64	7.79	4	0.78	no
C225T	0	0	2	0	2	0	0	12	12	14	2.75	42	16	0	3	0	9	0	11118700	187	1	0.53	694	54	7.78	4	0.78	no
C233T	0	1	2	0	3	0	0	13	13	16	3.14	22	20	0	5	0	4	0	18722400	185	4	2.16	659	49	7.44	1	0.20	no
C284T	1	1	3	1	6	0	0	10	10	16	3.14	53	18	0	4	0	15	0	13217000	187	0	0	694	45	6.48	0	0.00	no
C302T	0	0	1	0	1	0	0	13	13	14	2.75	81	118	0	19	0	9	4	24313700	190	1	0.53	605	47	7.77	1	0.20	no
C308T	0	1	1	0	2	0	0	17	17	19	3.73	49	174	1	21	0	6	2	28765200	185	5	2.7	702	61	8.69	0	0.00	no
C340T	2	2	2	0	6	0	0	20	20	26	5.10	174	32	0	5	0	20	3	25557900	186	4	2.15	766	51	6.66	5	0.98	no
C348T	1	0	2	0	3	0	0	15	15	18	3.53	7	0	0	0	0	1	1	11508300	185	4	2.16	753	39	5.18	0	0.00	no
C355T	51	5	1	2	59	1	0	53	54	113	22.16	47	0	0	0	0	5	0	30253700	189	156	82.54	478	109	22.8	177	34.71	yes
C362T	0	0	2	0	2	0	0	8	8	10	1.96	24	0	0	0	0	2	2	17561400	194	1	0.52	671	49	7.3	8	1.57	no
C378T	0	0	3	1	4	0	1	13	14	18	3.53	39	80	0	11	0	10	2	19568100	192	3	1.56	861	68	7.9	3	0.59	no
C384T	4	2	1	0	7	1	1	10	12	19	3.73	91	65	0	14	0	18	6	12992700	194	2	1.03	852	74	8.69	2	0.39	no
C390T	1	1	2	1	5	0	0	21	21	26	5.10	67	37	0	12	0	12	2	14958100	188	5	2.66	711	56	7.88	1	0.20	no
C401T	0	1	1	0	2	0	0	12	12	14	2.75	30	1	0	1	0	4	1	18099100	193	2	1.04	798	88	11.03	3	0.59	no
C410T	1	0	0	0	1	0	0	3	3	4	0.78	6	1	0	1	0	5	2	47759.3	193	1	0.52	835	62	7.43	3	0.59	no
C411T	0	0	0	0	0	0	0	0	0	0	0	8	18	0	2	0	4	1	16614300	190	2	1.05	1084	89	8.21	1	0.20	no
C415T	10	2	7	3	22	0	0	131	131	153	30.00	4	20	0	3	0	3	1	16001700	185	61	32.97	1115	259	23.23	53	10.39	yes
C416T	1	0	5	0	6	0	0	10	10	16	3.14	69	77	0	12	0	15	2	22235100	189	3	1.59	1037	107	10.32	3	0.59	no
C418T	3	0	2	0	5	0	0	24	24	29	5.69	202	0	0	0	0	17	6	35618900	186	3	1.61	1000	76	7.6	3	0.59	no
C432T	1	0	2	1	4	0	0	17	17	21	4.12	194	11	0	4	0	18	4	31670000	187	3	1.6	859	79	9.2	3	0.59	no
C433T	6	5	1	1	13	1	0	28	29	42	8.24	6	3	0	2	0	5	0	88040	193	10	5.18	1061	46	4.34	43	8.43	no
C442T	0	1	2	2	5	0	0	19	19	24	4.71	45	8	0	2	0	11	2	10429500	192	2	1.04	1518	74	4.87	5	0.98	no
C443T	0	2	2	0	4	0	0	10	10	14	2.75	8	34	0	6	0	7	1	7965820	191	0	0	1274	94	7.38	6	1.18	no
C458T	1	0	1	1	3	0	1	14	15	18	3.53	22	87	0	15	0	7	2	13873100	181	4	2.21	1007	70	6.95	4	0.78	no
C464T	1	1	1	1	4	0	0	15	15	19	3.73	51	83	0	15	0	10	4	16777300	187	2	1.07	1027	72	7.01	6	1.18	no
C478T	1	0	2	0	3	0	0	16	16	19	3.73	126	0	0	0	0	16	4	19278400	187	1	0.53	897	73	8.14	2	0.39	no
C479T	0	0	5	0	5	0	0	9	9	14	2.75	65	61	0	12	0	11	1	21661300	188	0	0	882	91	10.32	5	0.98	no
C485T	40	14	4	2	60	1	0	122	123	183	35.88	7	2	0	1	0	5	1	993673	192	83	43.23	894	182	20.36	158	30.98	yes
C487T	0	0	0	0	0	0	0	0	0	0	0	11	0	0	0	0	5	0	3369950	184	1	0.54	1114	93	8.35	0	0.00	no
C495T	5	19	1	5	30	0	0	56	56	86	16.86	20	18	0	2	0	9	1	13321800	191	28	14.66	721	60	8.32	59	11.57	no
C500T	0	0	0	0	0	0	0	0	0	0	0	7	25	0	7	0	5	1	10851800	192	1	0.52	896	79	8.82	1	0.20	no
C507T	2	0	3	2	7	0	0	23	23	30	5.88	212	56	0	10	0	31	9	21862400	193	2	1.04	888	68	7.66	7	1.37	no
C509T	0	1	1	0	2	1	0	10	11	13	2.55	35	48	0	8	0	14	3	10576800	195	1	0.51	625	48	7.68	4	0.78	no
C515T	0	0	0	0	0	0	0	0	0	0	0	61	48	0	9	0	9	1	26453200	188	4	2.13	909	125	13.75	1	0.20	no
Median	1.0	0.0	1.5	0.0	4.0	0.0	0.0	13.5	14.5	18.5	3.6	37.0	22.5	0.0	5.0	0.0	9.5	2.0	15479900.0	190.5	2.0	1.1	1.1	1.1	1.1	1.1	1.1	

Supplementary Table S4: Summary of functionally important germline mutations in individual genes

<table>
<tr><th>Chr</th><th>Gene.refGene</th><th>Start</th><th>End</th><th>Ref</th><th>Alt</th><th>Func.refGene</th><th>avsnp150</th><th>SIFT_pred</th><th>Polyphen2_HDIV_pred</th><th>CLNDN</th><th>CLNREVSTAT</th><th>CLNSIG</th><th>InterVar_automated</th></tr>
<tr><td>chr2</td><td>*SCN1A*</td><td>166053034</td><td>166053034</td><td>C</td><td>T</td><td>intronic</td><td>rs3812718</td><td>.</td><td>.</td><td>Early_infantile_epileptic_encephalopathy_with_suppression_bursts|FEBRILE_SEIZURES,_FAMILIAL,_3A,_SUSCEPTIBILITY_TO|carbamazepine_response_-_Dosage</td><td>reviewed_by_expert_panel</td><td>**drug_response**</td><td>.</td></tr>
<tr><td>chr1</td><td>*DPYD*</td><td>97883329</td><td>97883329</td><td>A</td><td>G</td><td>exonic</td><td>rs1801265</td><td>.</td><td>.</td><td>capecitabine_response_-_Toxicity|fluorouracil_response_-_Toxicity|Dihydropyrimidine_dehydrogenase_deficiency|not_specified|not_provided</td><td>reviewed_by_expert_panel</td><td>drug_response</td><td>**Likely pathogenic**</td></tr>
<tr><td>chr1</td><td>*DPYD*</td><td>97515839</td><td>97515839</td><td>T</td><td>C</td><td>exonic</td><td>rs1801159</td><td>Tolerated</td><td>Benign</td><td>capecitabine_response_-_Toxicity|fluorouracil_response_-_Toxicity|Dihydropyrimidine_dehydrogenase_deficiency|not_specified|not_provided</td><td>reviewed_by_expert_panel</td><td>**drug_response**</td><td>Benign</td></tr>
<tr><td>chr1</td><td>*DPYD*</td><td>97699535</td><td>97699535</td><td>T</td><td>C</td><td>exonic</td><td>rs2297595</td><td>Deleterious</td><td>Deleterious</td><td>capecitabine_response_-_Toxicity|fluorouracil_response_-_Toxicity|Dihydropyrimidine_dehydrogenase_deficiency|not_specified|not_provided</td><td>reviewed_by_expert_panel</td><td>**drug_response**</td><td>Benign</td></tr>
<tr><td>chr1</td><td>*DPYD*</td><td>97305364</td><td>97305364</td><td>C</td><td>T</td><td>exonic</td><td>rs1801160</td><td>Tolerated</td><td>Deleterious</td><td>capecitabine_response_-_Toxicity|fluorouracil_response_-_Other|fluorouracil_response_-_Toxicity|Dihydropyrimidine_dehydrogenase_deficiency|not_specified|not_provided</td><td>reviewed_by_expert_panel</td><td>**drug_response**</td><td>Likely benign</td></tr>
<tr><td>chr16</td><td>*ZFHX3*</td><td>72797459</td><td>72797461</td><td>TTG</td><td>-</td><td>exonic</td><td>rs372909378</td><td>.</td><td>.</td><td>Lung_cancer</td><td>no_assertion_criteria_provided</td><td>**Pathogenic**</td><td>.</td></tr>
<tr><td>chr7</td><td>*KMT2C*</td><td>152235860</td><td>152235860</td><td>C</td><td>T</td><td>exonic</td><td>rs199504848</td><td>Tolerated</td><td>Deleterious</td><td>Lung_cancer</td><td>no_assertion_criteria_provided</td><td>**Pathogenic**</td><td>Benign</td></tr>
<tr><td>chr7</td><td>*KMT2C*</td><td>152265209</td><td>152265209</td><td>G</td><td>A</td><td>exonic</td><td>rs763762478</td><td>Tolerated</td><td>Deleterious</td><td>Small_cell_lung_carcinoma</td><td>no_assertion_criteria_provided</td><td>**Pathogenic**</td><td>.</td></tr>
<tr><td>chr2</td><td>*CNTNAP5*</td><td>124524333</td><td>124524333</td><td>C</td><td>T</td><td>exonic</td><td>rs17727261</td><td>Deleterious</td><td>Benign</td><td>.</td><td>.</td><td>.</td><td>**Likely pathogenic**</td></tr>
<tr><td>chr2</td><td>*LRP2*</td><td>169140514</td><td>169140514</td><td>-</td><td>G</td><td>exonic</td><td>rs80338754</td><td>.</td><td>.</td><td>Donnai-Barrow_syndrome|not_provided</td><td>criteria_provided,_multiple_submitters,_no_conflicts</td><td>**Pathogenic/Likely_pathogenic**</td><td>.</td></tr>
<tr><td>chr16</td><td>*SMG1*</td><td>18925988</td><td>18925990</td><td>GCC</td><td>-</td><td>exonic</td><td>rs781029159</td><td>.</td><td>.</td><td>Hepatocellular_carcinoma</td><td>no_assertion_criteria_provided</td><td>**Pathogenic**</td><td>.</td></tr>
</table>

Supplementary Table S5: List of genes used for evaluation of somatic variants in selected oncodriver pathways

For pathway analysis, gene lists from Sanchez-Vega et al. 2018 (doi: 10.1016/j.cell.2018.03.035) and
Kim & Bodmer 2022 (doi: 10.1007/s00432-021-03888-w), supplemented by pathway definitions in KEGGs GSEA database v7.5.1, used.
HRD = homologou recombinantion deficiency according to Norquist et al. Clin Cancer Res 2018 (doi: 10.1158/1078-0432.CCR-17-1327)
ICB = immune checkpoint blockade (1 - four gene panel, 2 - gene panel extended by genes from seven pathway included in the
CIRCLE = the Cancer Immunotherapy Response CLassifiEr) according to Gajic et al. Nat Commun. 2022 (doi: 10.1038/s41467-022-31055-3)

Pathways - genes used in combination for each pathway analysis

**Hippo**	**MYC**	**NOTCH**	**PI3K**	**TGF-Beta**	**RTK-RAS**	**HRD (Norquist et al. 2018)**	**ICB1 (Gajic et al. 2022)***	**ICB2 (Gajic et al. 2022)***
STK4	MAX	ARRDC1	EIF4EBP1	TGFBR1	ABL1	ATM	KRAS	KRAS
STK3	MGA	CNTN6	AKT1	TGFBR2	EGFR	ATR	BRAF	BRAF
SAV1	MLX	CREBBP	AKT2	ACVR2A	ERBB2	BARD1	TP53	TP53
LATS1	MLXIP	EP300	AKT3	ACVR1B	ERBB3	BLM	BCLAF1	BCLAF1
LATS2	MLXIPL	HES1	AKT1S1	SMAD2	ERBB4	BRCA1		PSMB5
MOB1A	MNT	HES2	DEPDC5	SMAD3	PDGFRA	BRCA2		PSMA6
MOB1B	MXD1	HES3	DEPTOR	SMAD4	PDGFRB	BRIP1		PSMC2
YAP1	MXD3	HES4	INPP4B		MET	CHEK2		PSMD7
WWTR1	MXD4	HES5	MAPKAP1		FGFR1	MRE11A		PSMA5
TEAD1	MXI1	HEY1	MLST8		FGFR2	NBN		UBA52
TEAD2	MYC	HEY2	MTOR		FGFR3	PALB2		PSMB2
TEAD3	MYCL	HEYL	NPRL2		FGFR4	RAD51C		PSMA7
TEAD4	MYCN	KAT2B	NPRL3		FLT3	RAD51D		PSMB4
PTPN14		KDM5A	PDK1		ALK	RBBP8		PMAIP1
NF2		NOTCH1	PIK3CA		RET	SLX4		E2F1
WWC1		NOTCH2	PIK3CB		ROS1	XRCC2		CALR
TAOK1		NOTCH3	PIK3R1		KIT			APOE
TAOK2		NOTCH4	PIK3R2		IGF1R			COLEC12
TAOK3		NOV	PIK3R3		NTRK1			ICAM1
CRB1		NRARP	PPP2R1A		NTRK2			ITGA2B
CRB2		PSEN2	PTEN		NTRK3			FGG
CRB3		LFNG	RHEB		SOS1			ITGB1
LLGL1		ITCH	RICTOR		GRB2			COL5A1
LLGL2		NCSTN	RPTOR		PTPN11			COL18A1
HMCN1		SPEN	RPS6		KRAS			VTN
SCRIB		JAG1	RPS6KB1		HRAS			GNB3
HIPK2		APH1A	STK11		NRAS			CSNK2B
FAT1		FBXW7	TSC1		RIT1			TUBA1C
FAT2		FHL1	TSC2		ARAF			GNAT2
FAT3		THBS2			BRAF			CCNE1
FAT4		HDAC2			RAF1			NOP56
DCHS1		MFAP2			RAC1			TUBB2B
DCHS2		CUL1			MAP2K1			ZFHX3
CSNK1E		RFNG			MAP2K2			HIST1H2BO
CSNK1D		NCOR1			MAPK1			HIST1H2BK
AJUBA		NCOR2			NF1			H2AFV
LIMD1		MFAP5			RASA1			AKT3
WTIP		HDAC1			CBL			CRKL
		NUMB			ERRFI1			PMAIP1
		JAG2			CBLB			CASP2
		MAML3			CBLC			FGF4
		MFNG			INSR			STAT1
		CIR1			INSRR			CD58
		CNTN1			IRS1			TEK
		MAML1			SOS2			
		MAML2			SHC1			
		NUMBL			SHC2			
		PSEN1			SHC3			
		PSENEN			SHC4			
		RBPJ			RASGRP1			
		RBPJL			RASGRP2			
		RBX1			RASGRP3			
		SAP30			RASGRP4			
		SKP1			RAPGEF1			
		SNW1			RAPGEF2			
		CTBP1			RASGRF1			
		CTBP2			RASGRF2			
		ADAM10			FNTA			
		APH1B			FNTB			
		ADAM17			RCE1			
		DLK1			ICMT			
		DLL1			MRAS			
		DLL3			PLXNB1			
		DLL4			MAPK3			
		DNER			ARHGAP35			
		DTX1			RASA2			
		DTX2			RASA3			
		DTX3			RASAL1			
		DTX3L			RASAL2			
		DTX4			RASAL3			
		EGFL7			SPRED1			
					SPRED2			
					SPRED3			
					DAB2IP			
					SHOC2			
					PPP1CA			
					SCRIB			
					PIN1			
					KSR1			
					KSR2			
					PEBP1			
					ERF			
					PEA15			
					JAK2			
					IRS2			

Supplementary Table S6: List of prognostically significant genes

Gene	Approved name	HGNC ID	Location	Pharmacogenomics	Oncogenomics
**Somatic**					
ABCA13	ATP binding cassette subfamily A member 13	HGNC:14638	7p12.3	mutated in 5-FU	NOT in CGC & NOT Actionable
ANK2	ankyrin 2	HGNC:493	4q25-q26	mutated in platinum	NOT in CGC & NOT Actionable
COL6A3	collagen type VI alpha 3 chain	HGNC:2213	2q37.3	mutated in platinum	NOT in CGC & NOT Actionable
COL7A1	collagen type VII alpha 1 chain	HGNC:2214	3p21.31	mutated in 5-FU	NOT in CGC & NOT Actionable
FLG	filaggrin	HGNC:3748	1q21.3	mutated in platinum	NOT in CGC & NOT Actionable
GLI3	GLI family zinc finger 3	HGNC:4319	7p14.1	mutated in platinum	NOT in CGC & NOT Actionable
LRP1B	LDL receptor related protein 1B	HGNC:6693	2q22.1-q22.2	mutated in 5-FU	CGC – TSG for ovarian ca, oesophageal sqamous ca, urothelial ca; NOT Actionable
NAV3	neuron navigator 3	HGNC:15998	12q21.2	mutated in 5-FU	NOT in CGC & NOT Actionable
RYR1	ryanodine receptor 1	HGNC:10483	19q13.2	mutated in 5-FU	NOT in CGC & NOT Actionable
RYR3	ryanodine receptor 3	HGNC:10485	15q13.3-q14	mutated in 5-FU	NOT in CGC & NOT Actionable
TENM4	teneurin transmembrane protein 4	HGNC:29945	11q14.1	mutated in platinum	NOT in CGC & NOT Actionable
TCHH	trichohyalin	HGNC:11791	1q21.3	mutated in 5-FU	NOT in CGC & NOT Actionable
UNC80	unc-80 homolog, NALCN channel complex subunit	HGNC:26582	2q34	mutated in platinum	NOT in CGC & NOT Actionable
**Germline**					
ERBB4	erb-b2 receptor tyrosine kinase 4	HGNC:3432	2q34	mutated in platinum	CGC – TSG or oncogene for melanoma, gastric ca, non small cell lung ca; Actionable for lapatinib, neratinib, trametinib, palbociclib, everolimus (and their combinations)
NFASC	neurofascin	HGNC:29866	1q32.1	mutated in 5-FU	NOT in CGC & NOT Actionable
RIF1	replication timing regulatory factor 1	HGNC:23207	2q23.3	mutated in platinum	NOT in CGC & NOT Actionable
